# Crystalline boron-linked tetraaminoethylene radical cations†
†Electronic supplementary information (ESI) available: NMR spectra, crystallographic data, crystal structures, UV-vis spectra, cyclic voltammograms, EPR spectra, and theoretical calculation. CCDC 1548460–1548466. For ESI and crystallographic data in CIF or other electronic format see DOI: 10.1039/c7sc03528d



**DOI:** 10.1039/c7sc03528d

**Published:** 2017-09-12

**Authors:** Yuanting Su, Yongxin Li, Rakesh Ganguly, Rei Kinjo

**Affiliations:** a Division of Chemistry and Biological Chemistry , School of Physical and Mathematical Sciences , Nanyang Technological University , Nanyang Link 21 , Singapore 637371 . Email: rkinjo@ntu.edu.sg

## Abstract

Single-electron oxidation of neutral boryl-linked tetraaminoethylene derivatives **4** led to the formation of radical cations **4˙^+^**, which have been isolated and fully characterized. X-ray diffraction analysis, EPR spectroscopy, and computational studies revealed that the unpaired electron is delocalized over the B_2_N_4_C_2_ skeleton and the spin density mainly resides on the carbon and boron atoms.

## Abstract 2

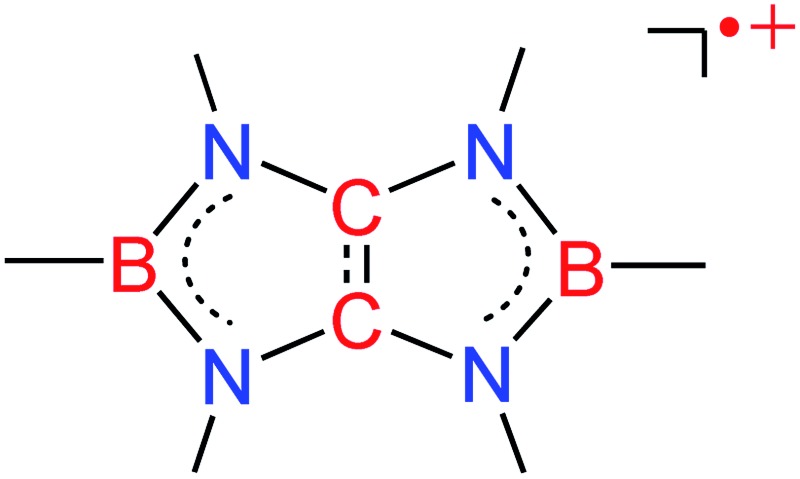
Boron-linked tetraaminoethylene radical cations has been isolated.

## Introduction

**Fig. 1:**
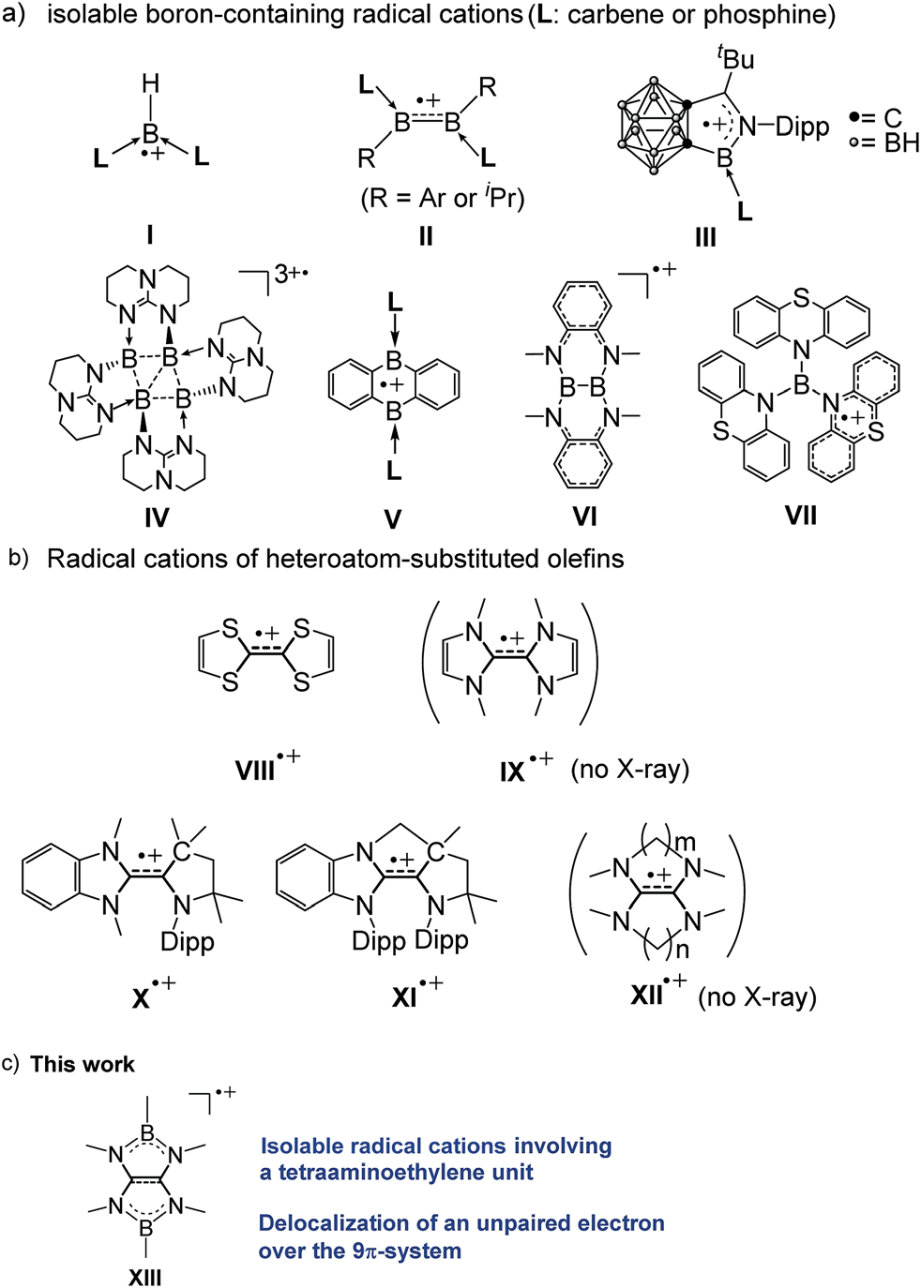
(a) Structurally characterized boron-containing radical cations. (b) Examples of radical cations of electron-rich olefins (Dipp = 2,6-diisopropylphenyl). (c) Present work.

Boron-containing radicals have been extensively studied due to their importance in fundamental chemistry and promising applications in various organic syntheses involving radical reactions.^1^ Thus far, a number of stable anionic^2^ and neutral^3^ boron radicals have been reported. By stark contrast, isolable boron radical cations are extremely rare probably due to the intrinsic electron deficient nature of boron. Indeed, only a handful of boron radical cations supported by the strong electron donors L have been isolated and structurally characterized by the Bertrand (**I**),^4^ Braunschweig (**II**),^5^ Xie (**III**),^6^ Himmel (**IV**),^7^ and Harman (**V**)^8^ groups (Fig. 1a). While two boron-containing radical cations (**VI** ^9^ and **VII** ^10^) without Lewis base stabilization have also been reported, the unpaired electron was most likely localized in the substituents rather than the boron atoms.

Owing to their peculiar ability to serve as powerful organic reducing reagents, electron-rich olefins (EROs) have received a surging interest more than two decades.^11,12^ During the redox reactions with EROs, the corresponding oxidized forms of EROs, especially the radical cations are concomitantly generated and therefore have been considered as key species (Fig. 1b). Among them, the radical cation of tetrathiafulvalene (TTF) **VIII** ^13^ has been extensively studied owing to its diverse utilities in various applications.^14–17^ Compared to TTF, tetraazafulvalenes (TAFs) **IX** feature more negative potentials^18^ which makes them attractive organic super-electron donors^11d–f,19^ and organocatalysts.^12^ However, their corresponding radical cations **IX˙^+^** have never been isolated thus far, mainly due to the facility of two-electron oxidation process attributed to the small electronic coupling. Recently, the Bertrand group reported two isolable triazaolefin radical cations (**X˙^+^** and **XI˙^+^**) and demonstrated the methylene-tethered **XI˙^+^** exhibits a larger electronic coupling.^20^ Similarly, it has been reported that aliphatic tetraaminoethylene (TAEs) **XII** linked by (poly)methylene chains increases the electronic coupling, which is, however, still not large enough to isolate the cation radical species **XII˙^+^**, and indeed no structural authentication of such species has been done to date.^11d–f,19^ Because of the isoelectronic and isosteric relationships between the C=C and B–N units, boron atoms binding to nitrogen atoms have readily been incorporated into π-conjugation system.^21,22^ Accordingly, we envisaged that linking the nitrogen atoms of tetraaminoethylene by two boryl groups may effectively increase the electron coupling, and the corresponding radical cation could be stabilized through delocalization of both the positive charge and the unpaired electron over the B_2_C_2_N_4_ framework as it should mitigate the electron deficiency of the boron centers. Consequently, synthetically challenging Lewis bases-free boron radical cations would be accessible. Herein, we show that in fact radical cations **XIII** involving a tetraaminoalkene unit (Fig. 1c) can be isolated. Their spectroscopic properties and structures are also presented.

## Results and discussion

**Scheme 1:**
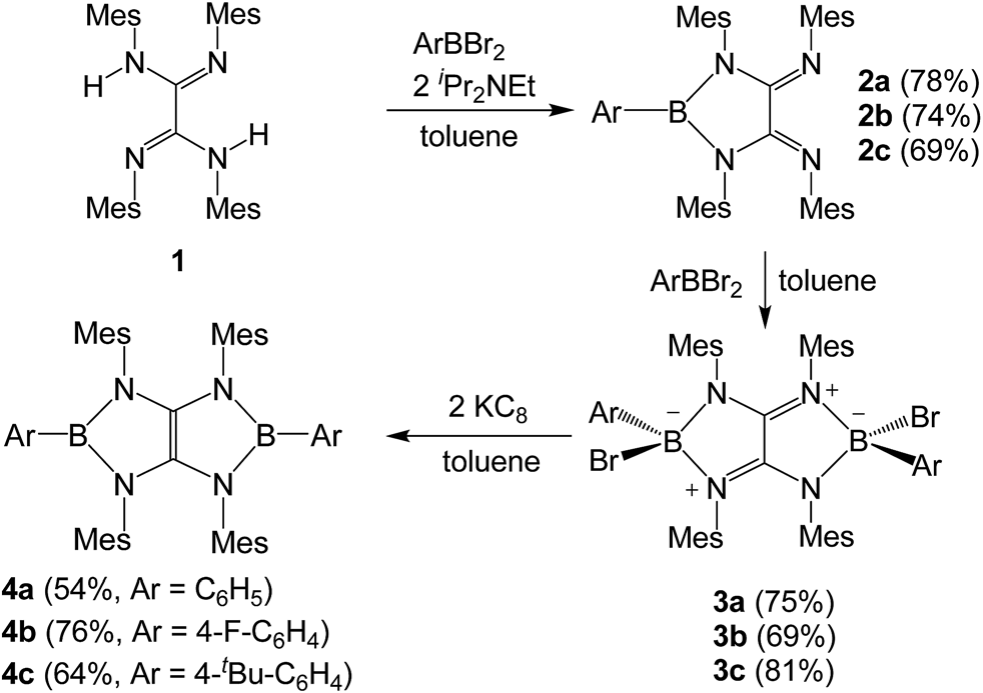
Synthesis of compound **4** (Mes = 2,4,6-trimethylphenyl).

Treatment of tetraaryloxalamidine **1** (ref. 23) with one equivalent of dibromophenylborane in the presence of two equivalents of diisopropylethylamine in toluene afforded **2a** as a yellow powder in 78% yield. A subsequent reaction between **2a** and a stoichiometric amount of dibromophenylborane in toluene immediately afforded a brown precipitate, which was collected by filtration and then washed with hexane to give **3a** as a white solid in 75% yield. A toluene solution of **3a** with two equivalents of potassium graphite (KC_8_) was stirred overnight under ambient condition, and after work up, boryl-linked tetraazaolefin derivative **4a** was obtained as a yellowish green powder in 54% yield (Scheme 1). The ^11^B NMR spectrum of **4a** displays a broad singlet at 25.4 ppm, which is shifted downfield with respect to that (9.8 ppm) of **3a**. Compound **4a** is thermally stable both in the solid state and in solutions, and it melts at 339 °C without decomposition. When *para*-substituted dibromophenylboranes with F and ^*t*^Bu groups were employed under the same reaction procedures, the corresponding derivatives **4b** and **4c** were obtained in moderate yields. In the ^11^B NMR spectra, a broad singlet appears at *δ* = 26.4 ppm (**4b**) and *δ* = 26.1 ppm (**4c**), respectively. Compounds **4a–c** can be deemed inorganic/organic hybrid versions of pentalene dianion,^24^ which has been widely utilized as an ancillary ligand of metal complexes.^25^

**Fig. 2:**
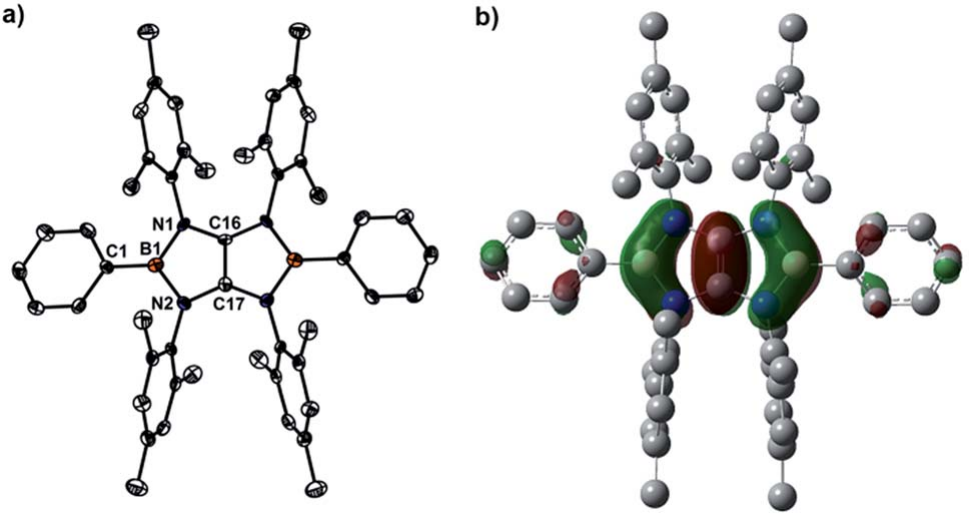
(a) Solid-state structures of **4a** (hydrogen atoms are omitted for clarity). Thermal ellipsoids are set at the 50% probability level. Selected bond lengths [Å] and angles [°]: B1–C1 1.551(6), B1–N1 1.460(5), B1–N2 1.460(5), C16–N1 1.396(3), C17–N2 1.397(4), C16–C17 1.323(7), C1–B1–N1 127.1(4), C1–B1–N2 127.1(4), N1–B1–N2 105.8(4). (b) The plot of the HOMO of **4a**.

X-ray diffraction analysis of **4a** confirmed the essentially planar B_2_N_4_C_2_ framework with a propeller-like orientation of the six aryl groups (Fig. 2a). Two boron atoms adopt perfectly trigonal-planar geometry (*Σ*_B_ = 360°) with the N1–B1–N2 bond angle of 105.8(4)°. The equal B–N (1.460(5) Å) bond distances are significantly shorter than the corresponding bonds (1.581(4) Å and 1.585(4) Å) in **3a** (Fig. S71†) and in the range between typical B–N single bond (1.50 Å) and B=N double bond (1.31 Å), indicating the partial multiple bond property. Compared to those of **3a**, there are lengthening of the C16–N1 bond (1.396(3) Å) and the C17–N2 bond (1.397(4) Å) and a markedly shortening of the C16–C17 bond (1.323(7) Å), which is close to those found in tetrakis(dimethylamino)ethylene (TDAE) (1.350(2) Å),^26^ **X** (1.3459(16) Å) and **XI** (1.344(2) Å).^20^ Compound **4b** (Fig. S72†) exhibits metric parameters similar to those of **4a**. DFT calculation performed on **4a** at the M062X/6-31G(d,p) level of theory shows that the HOMO of **4a** corresponds to the π-system over the B_2_C_2_N_4_ skeleton featuring a node between each NBN and the central CC π-unit (Fig. 2b). Natural bond orbital (NBO) analysis gave Wiberg bond index values of the C–C bond (1.51), the B–N bonds (0.94 and 0.93) and the C–N bonds (1.05 and 1.05) of the B_2_C_2_N_4_ skeleton.

Cyclic voltammetry of **4a** in CH_2_Cl_2_ (0.1 M ^*n*^Bu_4_NPF_6_ as the electrolyte) showed two well-separated oxidation waves (Fig. S79†), which is in sharp contrast to TAFs **IX** exhibiting very close redox waves (Δ*E*_1/2_ < 0.3 V).^11d–f,18^ The first oxidation of **4a** at *E*_1/2_ = –0.44 V (referenced against the ferrocene/ferrocenium (Fc/Fc^+^) couple) was found to be a reversible process associated with the formation of the radical cation species whereas the second oxidation centered at *E*_1/2_ = 0.43 V was irreversible. Cyclic voltammograms of **4b** and **4c** show similar oxidation events with the first reversible (–0.44 V for **4b** and –0.56 V for **4c**) and second irreversible (0.41 V for **4b** and 0.33 V for **4c**) waves, respectively (Fig. S80 and 81†).

The first oxidation potentials of **4a–c** are less negative than those of tetraazafuvalenes **IX** (around –1.00 V *vs.* SCE, Fc/Fc^+^: *E*_1/2_ = 0.46 V *vs.* SCE). Thus, upon incorporation of the boryl groups, the reducing power of compounds **4a–c** becomes weaker than that of tetraazafuvalenes **IX**.

**Scheme 2:**
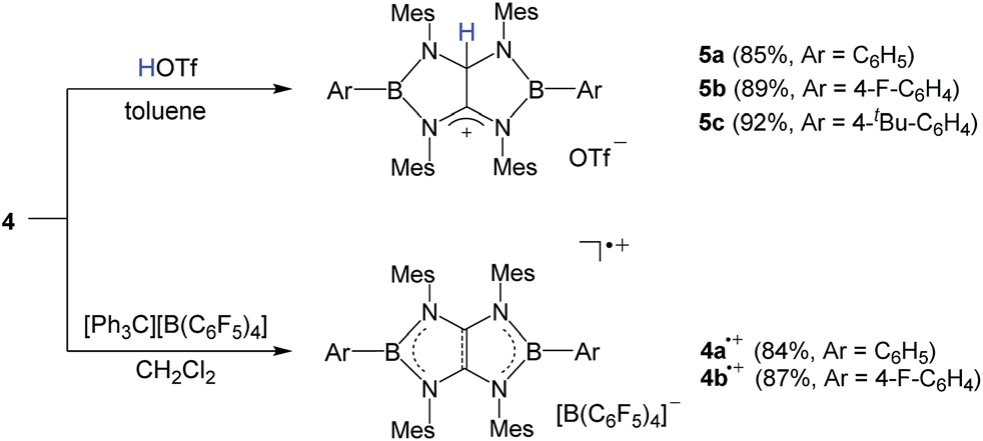
Synthesis of **4˙^+^** and **5**.

**Fig. 3:**
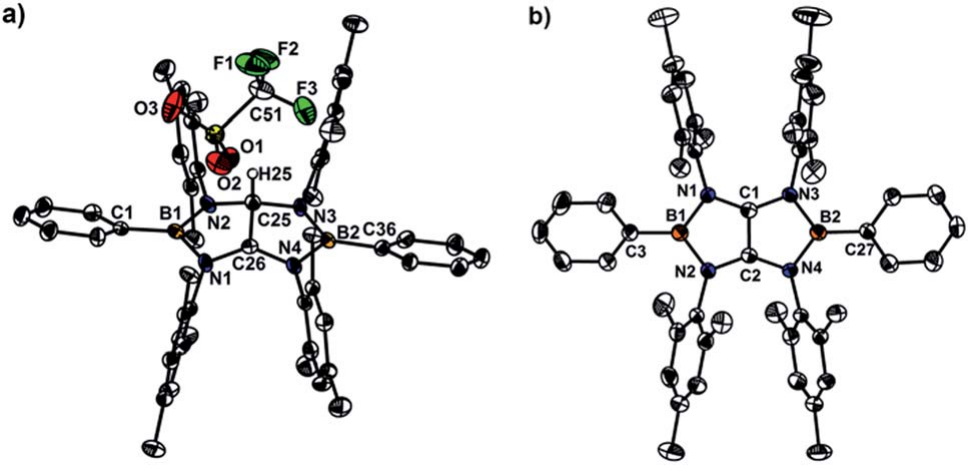
(a) Solid-state structures of **5a** (hydrogen atoms are omitted for clarity). Thermal ellipsoids are set at the 50% probability level. Selected bond lengths [Å] and angles [°]: B1–N1 1.514(6), B1–N2 1.422(6), B1–C1 1.546(7), B2–N3 1.430(6), B2–N4 1.489(6), B2–C36 1.542(7), N1–C26 1.330(5), N2–C25 1.469(5), N3–C25 1.484(5), N4–C26 1.365(5), C25–C26 1.490(5), N1–B1–N2 107.3(4), N1–B1–C1 123.3(4), N2–B1–C1 129.2(4), N3–B2–N4 107.0(4), N3–B2–C36 126.7(4), N4–B2–C36 1276.3(4). (b) Solid-state structures of **4a˙^+^** (hydrogen atoms are omitted for clarity). Thermal ellipsoids are set at the 50% probability level. Selected bond lengths [Å] and angles [°]: B1–C3 1.545(3), B1–N1 1.458(3), B1–N2 1.474(3), B2–N3 1.464(2), B2–N4 1.472(2), C1–N1 1.356(3), C1–N3 1.365(2), C2–N2 1.356(2), C2–N4 1.352(2), C1–C2 1.414(3), C3–B1–N1 126.64(17), C3–B1–N2 126.69 (17), N1–B1–N2 106.66(16), C27–B2–N3 125.82(16), C27–B2–N4 127.73(17), N3–B2–N4 106.40(15).

It has been reported that tetraaminoethylene derivative typically undergoes the addition reaction with an electrophile at the central C=C moiety.^27^ To investigate the chemical behavior of **4**, we performed their reactions with electrophiles. Treatment of a toluene solution of **4a** with an equimolar amount of trifluoromethane sulfonic acid (HOTf) at room temperature afforded the corresponding conjugated acid **5a** as a white powder in 85% yield (Scheme 2). The ^11^B NMR spectrum displays a broad singlet at 38.1 ppm which is shifted downfield with respect to that (25.4 ppm) of **4a**. In the ^1^H and ^13^C NMR spectra, a singlet at 7.71 ppm for NC*H*N proton and 82.2 ppm and 187.4 ppm for the corresponding N*C*HN and N*C*N carbons were observed, respectively. Under the same conditions, **4b** and **4c** underwent protonation with HOTf to afford the respective products **5b** and **5c** in good yields. An X-ray diffraction studies of **5a** revealed that both boron centers feature the trigonal-planar geometry. One of the central carbon atoms in the B_2_C_2_N_4_ ring is in the tetragonal configuration with a hydrogen atom whereas its adjacent cationic carbon exhibits the trigonal-planar geometry with a formal charge of +1 (Fig. 3a). The distance of the C25–C26 (1.490(5) Å) bond is significantly longer than the corresponding bond (1.323(7) Å) in **4a**, and comparable to typical single C–C bond length. These results demonstrate the tetraaminoethylene-type nature of **4**.

In line with the oxidation potentials, chemical single-electron oxidations of **4a** and **4b** were readily accomplished by using of [Ph_3_C][B(C_6_F_5_)_4_] as an oxidizing agent. Reactions of **4a** and **4b** with [Ph_3_C][B(C_6_F_5_)_4_] in CH_2_Cl_2_ at ambient temperature smoothly generated **4a˙^+^** and **4b˙^+^** as NMR-silent red (**4a˙^+^**: 84%) and reddish brown powder (**4b˙^+^**: 87%), respectively (Scheme 2). Crystals suitable for X-ray crystallographic studies were obtained by recrystallization from a mixture of CH_2_Cl_2_ and toluene solution. Like the neutral **4a** and **4b**, **4a˙^+^** and **4b˙^+^** bearing the 9π-system retain the planar B_2_N_4_C_2_ skeletons with trigonal planar sp^2^ boron atoms (Fig. 3b and S73†). Upon oxidation of **4a** to **4a˙^+^**, the B–N bonds (1.458(3)–1.474(3) Å) and the C=C (1.414(3) Å) bond are very slightly lengthened, while the endocyclic C–N bonds (1.352(2)–1.365(2) Å) are shortened, which is in good agreement with the electronic structure of **4**. Thus, upon removal of one electron from the HOMO of **4** (Fig. 2b), the electron density in the C=C and N–B–N π-bonding orbitals decreases, leading to the elongation of the C=C and B–N distances, while the repulsion between the bonding (N–B–N and C=C) electron pairs decreases resulting in the shortening of the C–N bond distances. Note that **4˙^+^** represent the first example of radical cations derived from BN heterocycles featuring some aromatic nature (Fig. S84, Table S2†).^21,28^ Moreover, **4˙^+^** correspond to the anion radical of organic pentalene, among which only the derivatives featuring thermodynamically highly stabilizing benzo substituents have been structurally characterized.^29^

**Fig. 4:**
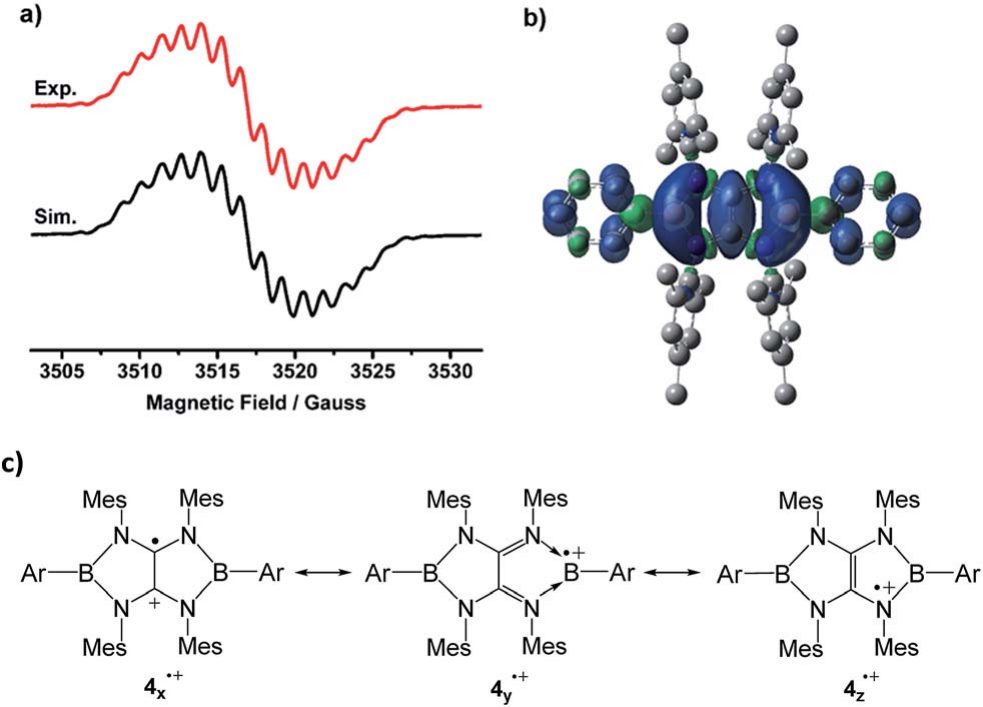
(a) Experimental (red) and simulated (black) EPR spectra of **4a˙^+^** in fluorobenzene at room temperature. (b) The plot of the spin density of **4a˙^+^**. (c) The selected resonance forms of **4˙^+^**.

The EPR spectrum of **4a˙^+^** measured in fluorobenzene at room temperature displays a complex system (*g* = 2.0050) arising from couplings with two boron (*a*(^11^B) = 1.16 G, *a*(^10^B) = 0.63 G), four nitrogen (*a*(^14^N) = 1.53 G), and four hydrogen atoms (*a*(^1^H) = 1.32 G) at the *ortho*-positions of phenyl groups (Fig. 4a). The ^11^B hyperfine coupling constant is smaller than those of **I** (6.43 G)^4^ and isolable boron radical anions,^2^ but comparable to those of **II** (≤1.18 G)^5^ and reported stable neutral boron radicals (0.96–8.5 G).^3^ The moderate *a*(^11^B) coupling constant together with relative small *a*(^14^N) values suggests delocalization of the unpaired electron over the B_2_N_4_C_2_ core. To gain more insight into the electronic structure of **4a˙^+^**, unrestricted DFT calculations were performed at the UM062X/6-31G(d,p) level. NBO method confirmed that the spin density is entirely delocalized over the B_2_N_4_C_2_ framework with some extension to the two Ph groups on the B atoms (Fig. 4b). The spin density is estimated to be present mainly on the carbon (0.26*e* × 2) and the boron (0.19*e* × 2) atoms with a relatively small contribution of the nitrogen atoms (0.02*e* × 4). While the several resonance forms including **4*_x_* ˙^+^**, **4*_y_* ˙^+^**, **4*_z_* ˙^+^** can be drawn for **4˙^+^** (Fig. 4c), the X-ray diffraction analysis, ESR spectra in combination with DFT results indicate that compared with the N-centered radical form **4*_z_* ˙^+^**, contribution of others including the tetraaminoalkene radical cation **4*_x_* ˙^+^** and boron radical cation **4*_y_* ˙^+^** is more significant to the actual electronic structure of **4˙^+^**.

## Conclusions

In summary, we have shown that boryl-tethered tetraaminoalkene derivatives **4** can be readily synthesized and they undergo protonation reaction and one-electron oxidation reaction. The former afforded the corresponding salts **5** whereas the latter led to the formation of isolable radical cations **4˙^+^**. X-ray diffraction analysis, EPR spectra as well as computational studies of **4˙^+^** revealed the unpaired electron delocalized over the B_2_N_4_C_2_ framework mainly resides on the carbon and boron atoms.

## Conflicts of interest

There are no conflicts to declare.
