# Supplementary material for: Crystalline boron-linked tetraaminoethylene radical cations
Source: Chem Sci. 2017 Sep 12;8(11):7419–23. doi: 10.1039/c7sc03528d (PMC5674142; doi:10.1039/c7sc03528d)
Supplement: Supplementary file 1 [file SC-008-C7SC03528D-s001.pdf]

Electronic Supplementary Material (ESI) for Chemical Science.
This journal is © The Royal Society of Chemistry 2017

Crystalline Boron-linked Tetraaminoethylene Radical Cations

Yuanting Su Yongxin Li, Rakesh Ganguly, and Rei Kinjo

Table of Contents

1. Synthesis, physical and spectroscopic data for all new compounds
2. Crystal structural parameters and solid structures
3. UV-Vis absorption and photoluminescence spectroscopies
4. Cyclic voltammograms
5. EPR spectra
6. Theoretical calculations
7. References

1. Synthesis, physical and spectroscopic data for all new compounds

General Procedures. All experiments were carried out under an argon or nitrogen atmosphere using standard Schlenk or dry glovebox techniques. Solvents were dried over Na metal, K metal, or CaH_2, and were distilled under nitrogen prior to use. Reagents were of analytical grade, obtained from commercial suppliers and used without further purification. ^{1}H, ^{11}B, ^{13}C and ^{19}F NMR were obtained with a Bruker AVIII 400 MHz BBFO1 spectrometer at 298 K unless otherwise stated. Chemical shifts (δ) are given in p.p.m. Coupling constants *J* are given in Hz. NMR multiplicities are abbreviated as follows: s = singlet, d = doublet, t = triplet, m = multiplet, br = broad signal. Most of signals for the quaternary carbon atoms bonding to boron atom could not be detected, presumably due to coupling with the boron atom. Electrospray ionization (ESI) mass spectra were obtained at the Mass Spectrometry Laboratory at the Division of Chemistry and Biological Chemistry, Nanyang Technological University. Melting points were measured with an OpticMelt (Stanford Research System). UV-Vis absorption and fluorescence emission spectra were carried out with Cary 300 UV-Vis and Cary Eclipse spectrometer, respectively. Cyclic voltammetry (CV) was performed on a Biologic SP-50 electrochemical analyzer in anhydrous dichloromethane containing recrystallized tetra-*n*-butyl-ammoniumhexafluorophosphate ($TBAPF_6$, 0.1M) as supporting electrolyte at 298 K. A conventional three electrode cell was used with a glassy carbon electrode as the auxiliary electrode, silver/silver nitrate as the reference electrode, and a platinum wire as the working electrode. All electrochemical measurements were carried out under an atmospheric pressure of nitrogen. Continuous wave X-band electron paramagnetic resonance (EPR) spectra were recorded on a Bruker ELEXSYS E500 EPR spectrometer. Dibromophenylborane,[S1] 4-fluoro-dibromophenylborane,[S2] 4-tert-butyl-dibromophenylborane,[S3] and Dimesityloxaldiimidoyl dichloride[S4] were prepared according to the literature procedures.

Synthesis of compound 1: To a solution of dimesityloxalimidoyl dichloride (36.13 g, 100 mmol) in toluene (600 ml), 2,4,6-trimethylaniline (81.12 g, 600 mmol) was added at room temperature. The mixture was heated under reflux for two days. After cooling down and filtration, the solvent was concentrated in vacuo. The addition of hexane resulted in the formation of a yellow precipitate. The precipitate was separated by filtration and then washed three times with hexane (3×50 ml) to give **1** as a white solid (50.85 g, 91%). **M.p.**: 246 ^{o}C ; ^{1}**H NMR** ($CDCl_3$, 400 MHz, 298 K): δ 6.60 (s, 8H, Ar-C*H*), 2.15 (s, 12H, *p*-CH_3), 2.10 (s, 24H, *o*-CH_3); ^{13}**C{^{1}H} NMR** ($CDCl_3$, 100 MHz, 298 K): δ 128.2 (Ar-*C*H), 20.7 (*C*H_3), 18.8 (*C*H_3); **HRMS** (ESI): m/z calcd for $C_{38}H_{47}N_4$: 559.3801 $[(M+H)]^{+}$; found : 559.3802.

Synthesis of compound 2a: A solution of dibromophenylborane (4.95 g, 20 mmol) in toluene (50 ml) was added dropwise to a chilled solution (0 ^{o}C) of diisopropylethylamine (5.17 g, 40 mmol) in toluene (50 ml) whereby colorless solids precitipated. After the mixture was warmed to room temperature, the suspension of compound **1** (11.18 g, 20 mmol) in toluene (40 ml) was added and the resulting mixture was heated under reflux overnight. After cooling down to room temperature and filtration, the solvent was removed under vacuum and the solid residue was washed with hexane (3×10 ml) and dried under vacuum to afford **2a** as a yellow solid (10.04 g, 78%). Single crystals suitable for X-ray diffraction studies were grown from a saturated hexane

solution at room temperature. **M.p.**: 240 ^{o}C; **^{1}H NMR** (C_6D_6, 400 MHz, 298 K): δ 7.22 (d, J = 8.0 Hz, 2H. o-C*H*), 6.87-6.79 (m, 3H, *m*-C*H* and *p*-C*H*), 6.66 (s, 4H, Ar-*H*), 6.49 (s, 4H, Ar-*H*), 2.32 (s, 12H, *m*-C*H*$_3$), 2.15 (s, 12H, *m*-C*H*$_3$), 2.07 (s, 6H, *p*-C*H*$_3$), 2.05 (s, 6H, *p*-C*H*$_3$); **^{13}C NMR** (C_6D_6, 100 MHz, 298 K): δ 136.7 (Ar-*C*), 135.4 (Ar-*C*), 135.1 (Ar-*C*), 133.7 (Ar-*C*H), 131.1 (Ar-*C*H), 130.5 (Ar-*C*), 129.4 (Ar-*C*H), 128.2 (Ar-*C*H), 128.1 (Ar-*C*H), 125.0 (Ar-*C*), 21.0 (Ar-*C*H$_3$), 20.1 (Ar-*C*H$_3$), 19.4 (Ar-*C*H$_3$), 19.1 (Ar-*C*H$_3$); **^{11}B NMR** (C_6D_6, 128.3 MHz): δ 36.4 (br); **HRMS** (ESI): m/z calcd for $C_{44}H_{50}BN_4$: 645.4129 [(*M+H*)]$^+$; found : 645.4138.

Compound 2b: yellow powder (74 %), **M.p.**: 220 ^{o}C; **^{1}H NMR** (C_6D_6, 400 MHz, 298 K): δ 7.10-7.06 (m, 2H. Ar-C*H*), 6.68 (s, 4H, Ar-C*H*), 6.49 (s, 4H, Ar-*H*), 6.40 (t, *J* = 8.0 Hz, 2H, Ar-*H*), 2.29 (s, 12H, Ar-C*H*$_3$), 2.14 (s, 12H, Ar-C*H*$_3$), 2.08 (s, 6H, Ar-C*H*$_3$), 2.06 (s, 6H, Ar-C*H*$_3$); **^{13}C{^{1}H} NMR** (C_6D_6, 100 MHz, 298 K): δ 162.7 (d, $^{1}J_{CF}$ = 245 Hz, Ar-*C*F), 158.5 (N*C*N), 137.5 (Ar-*C*), 134.6 (d, $^{3}J_{CF}$ = 7 Hz, Ar-*C*CCF), 133.0 (Ar-*C*), 130.0 (Ar-*C*H), 113.7 (d, $^{2}J_{CF}$ = 20 Hz, Ar-*C*CF), 20.7 (Ar-*C*H$_3$); **^{11}B NMR** (C_6D_6, 128.3 MHz): δ 36.1 (br); **^{19}F{^{1}H} NMR** (C_6D_6, 376 MHz): δ -115.0; **HRMS** (ESI): m/z calcd for $C_{44}H_{49}BN_4F$: 663.4034 [(*M+H*)]$^+$; found : 663.4042.

Compound 2c: yellow powder (69 %), **M.p.**: 258 ^{o}C; **^{1}H NMR** (C_6D_6, 400 MHz, 298 K): δ 7.25 (d, *J* = 8.0 Hz, 2H. Ar-C*H*), 6.94 (d, *J* = 8.0Hz, 2H, Ar-C*H*), 6.69 (s, 4H, Ar-*H*), 6.50 (s, 4H, Ar-*H*), 2.36 (s, 12H, Ar-C*H*$_3$), 2.16 (s, 12H, Ar-C*H*$_3$), 2.09 (s, 6H, Ar-C*H*$_3$), 2.06 (s, 6H, Ar-C*H*$_3$), 0.89 (s, 9H, C(C*H*$_3$)$_3$); **^{13}C{^{1}H} NMR** (C_6D_6, 100 MHz, 298 K): δ 154.3 (N*C*N), 136.7 (Ar-*C*), 135.5 (Ar-*C*), 134.1 (Ar-*C*H), 130.4 (Ar-*C*), 129.5 (Ar-*C*H), 128.2 (Ar-*C*H), 125.3 (Ar-*C*H), 125.0 (Ar-*C*), 34.6 (*C*(CH$_3$)$_3$, 30.9 (C(*C*H$_3$)$_3$, 21.0 (Ar-*C*H$_3$), 20.8 (Ar-*C*H$_3$), 19.5 (Ar-*C*H$_3$), 19.2 (Ar-*C*H$_3$); **^{11}B NMR** ($CDCl_3$, 128.3 MHz): δ 37.2 (br); **HRMS** (ESI): m/z calcd for: $C_{48}H_{58}BN_4$: 701.4755 [(*M+H*)]$^+$; found : 701.4762.

Synthesis of compound 3a: A toluene (10 ml) solution of dibromophenylborane (2.48 g, 10 mmol) was added dropwise to a toluene (50 ml) solution of **2a** (6.41 g, 9.94 mmol) at -30°C. The resulting mixture was warmed to room temperature and stirred overnight. The precipitate was separated by filtration and then washed with hexane (3×5 ml) to give **3a** as a white solid (6.67g, 75%). Single crystals suitable for X-ray diffraction studies were grown from a saturated chloroform solution at room temperature. **M.p.**: 209 ^{o}C (dec.); **^{1}H NMR** ($CDCl_3$, 400 MHz, 298 K): δ 7.63-7.60 (m, 4H, *o*-C*H*), 7.20-7.16 (m, 6H, *m*-C*H* and *p*-C*H*), 6.71 (s, 4H, *m*-C*H*), 6.13 (s, 4H, *m*-C*H*), 2.80 (s, 12H, *m*-C*H*$_3$), 2.04 (s, 12H, *m*-C*H*$_3$), 1.26 (s, 12H, *p*-C*H*$_3$); **^{13}C{^{1}H} NMR** ($CDCl_3$, 100 MHz, 298 K): δ 154.9 (N*C*N), 135.6 (Ar-*C*), 133.6 (Ar-*C*), 133.4 (Ar-*C*H), 128.9 (Ar-*C*H), 128.8 (Ar-*C*H), 127.2 (Ar-*C*H), 126.8 (Ar-*C*H), 22.2 (Ar-*C*H$_3$), 20.6 (Ar-*C*H$_3$), 19.8 (Ar-*C*H$_3$); **^{11}B NMR** (C_6D_6, 128.3 MHz): δ 9.8 (s); **HRMS** (ESI): m/z calcd for $C_{50}H_{55}{}^{10}B^{11}B^{81}Br_2N_4$: 894.2975 [(*M+H*)]$^+$; found : 894.2956.

Compound 3b: white powder (69%). **M.p.**: 256 ^{o}C (dec.); **^{1}H NMR** ($CDCl_3$, 400 MHz, 298 K): δ 7.20 (t, *J* = 8.0 Hz, 4H. Ar-C*H*), 6.68 (t, *J* = 8.0 Hz, 4H, Ar-C*H*), 6.55 (s, 8H, Ar-*H*), 2.15-2.11(m, 36H, Ar-C*H*$_3$); **^{13}C{^{1}H} NMR** ($CDCl_3$, 100 MHz, 298 K): δ 164.8 (d, $^{1}J_{CF}$ = 250 Hz, Ar-*C*F), 136.8 (Ar-*C*), 136.2 (d, $^{3}J_{CF}$ = 8 Hz, Ar-*C*CCF), 136.1 (Ar-*C*H), 135.3 (Ar-*C*), 135.0 (Ar-*C*), 130.6 (Ar-*C*), 129.5 (Ar-*C*), 128.2 (Ar-*C*H), 125.0 (Ar-*C*), 115.3 (d, $^{2}J_{CF}$ = 20 Hz, Ar-*C*CF), 21.0 (Ar-*C*H$_3$), 20.8 (Ar-*C*H$_3$), 19.4 (Ar-*C*H$_3$), 19.0 (Ar-*C*H$_3$); **^{11}B NMR** ($CDCl_3$, 128.3 MHz): δ 35.1 (br); **^{19}F{^{1}H}** NMR (C_6D_6, 376 MHz): δ -115.0; **HRMS** (ESI): m/z calcd for $C_{50}H_{53}B_2N_4{}^{79}Br^{81}BrF_2$: 929.2771 [(*M+H*)]$^+$; found : 929.2787.

Compound 3c: white powder (81%). **M.p.**: 222 ^{o}C (dec.); **^{1}H NMR** ($CDCl_3$, 400 MHz, 298 K): δ 7.15 (d, J = 8.0 Hz, 2H, Ar-C*H*), 6.98 (d, J = 8.0 Hz, 2H, Ar-C*H*), 6.53 (s, 8H, Ar-C*H*), 2.16 (s, 12H, Ar-CH_3), 2.14 (s, 24H, Ar-CH_3), 1.20 (s, 18H, C(CH_3)$_3$); **^{13}C{^{1}H} NMR** ($CDCl_3$, 100 MHz, 298 K): δ 158.3 (N*C*N), 150.3 (Ar-*C*), 137.2 (Ar-*C*), 135.5 (Ar-*C*), 133.2 (Ar-*C*), 132.5 (Ar-*C*H), 129.5 (Ar-*C*H), 123.6 (Ar-*C*H), 34.4 (*C*(CH_3)$_3$), 31.4 (C(*C*H$_3$)$_3$), 22.2 (Ar-*C*H$_3$), 20.7 (Ar-*C*H$_3$); **^{11}B NMR** (C_6D_6, 128.3 MHz): δ 10.8 (br); **HRMS** (ESI): m/z calcd for $C_{58}H_{71}B_2N_4{}^{79}Br^{81}Br$:1005.4211 [(*M+H*)]$^+$; found : 1005.4236.

Synthesis of compound 4a: Potassium graphite (0.54 g, 4.0 mmol) was slowly added to a toluene (40 ml) suspension of **3a** (1.79 g, 2.0 mmol) and stirred overnight at room temperature. After filtration, the solid residue was extracted with warm (80 ^{o}C)) toluene (50 ml). The combined solvent was removed under vacuum and the residue was washed with hexane (3×5 ml) to afford **4a** as yellow-green solid (0.80 g, 54%). Single crystals suitable for X-ray diffraction studies were grown from a saturated toluene solution at room temperature. **M.p.**: 339 ^{o}C; **^{1}H NMR** (C_6D_6, 400 MHz, 298 K): δ 7.28-7.26 (m, 4H, *o*-C*H*), 6.91-6.84 (m, 6H, *m*-C*H* and *p*-C*H*), 6.52 (s, 8H, *m*-C*H*), 2.22 (s, 24H, *m*-CH_3), 2.07 (s, 12H, *p*-CH_3); **^{13}C{^{1}H} NMR** ($CDCl_3$, 100 MHz, 298 K): δ 136.0 (Ar-*C*), 135.5 (Ar-*C*), 135.2 (Ar-*C*), 132.3 (Ar-*C*H), 128.2 (Ar-*C*H), 127.5 (Ar-*C*H), 127.4 (Ar-*C*H), 122.9 (Ar-*C*), 21.0 (Ar-*C*H$_3$), 18.7 (Ar-*C*H$_3$); **^{11}B NMR** ($CDCl_3$, 128.3 MHz): δ 25.4 (br); **UV-Vis** (CH_2Cl_2): λ = 374 nm (9290); **Fluorescence** (CH_2Cl_2): λ = 492 nm; **HRMS** (ESI): m/z calcd for $C_{50}H_{55}B_2N_4$: 733.4613 [(*M+H*)]$^+$; found : 733.4629.

Compound 4b: yellow-green powder (76 %). **M.p.**: 298 ^{o}C; **^{1}H NMR** (C_6D_6, 400 MHz, 298 K): δ 7.12-7.09 (m, 4H, Ar-C*H*), 6.54 (s, 8H, Ar-C*H*), 6.51-6.48 (m, 4H, Ar-C*H*), 2.18 (s, 24H, Ar-CH_3), 2.08 (s, 12H, Ar-CH_3); **^{13}C{^{1}H} NMR** (C_6D_6, 100 MHz, 298 K): δ 163.3 (d, $^{1}J_{CF}$ = 246 Hz, Ar-*C*F), 136.2 (Ar-*C*), 135.7 (Ar-*C*), 134.5 (d, $^{3}J_{CF}$ = 7 Hz, Ar-*C*CCF), 128.8 (Ar-*C*H), 123.3 (Ar-*C*), 115.0 (d, $^{2}J_{CF}$ = 20 Hz, Ar-*C*CF), 21.0 (Ar-*C*H$_3$), 18.8 (Ar-*C*H$_3$); **^{11}B NMR** (C_6D_6, 128.3 MHz): δ 26.4 (br); **^{19}F{^{1}H} NMR** (C_6D_6, 376 MHz): δ -112.6; **UV-Vis** (CH_2Cl_2): λ = 364 nm (7023); **Fluorescence** (CH_2Cl_2): λ = 487 nm; **HRMS** (ESI): m/z calcd for $C_{50}H_{53}B_2N_4F_2$: 769.4424 [(*M+H*)]$^+$; found : 769.4445.

Compound 4c: yellow-green powder (64%). **M.p.**: 382 ^{o}C; **^{1}H NMR** (C_6D_6, 400 MHz, 298 K): δ 7.26 (d, J = 8.0 Hz, 4H, Ar-C*H*), 6.96 (d, J = 8.0 Hz, 4H, Ar-C*H*), 6.56 (s, 8H, Ar-C*H*), 2.36 (s, 24H, Ar-CH_3), 2.11 (s, 12H, Ar-CH_3), 0.96 (s, 18H, C(CH_3)$_3$); **^{13}C{^{1}H} NMR** (C_6D_6, 100 MHz, 298 K): δ 150.51 (N*C*N), 136.9 (Ar-*C*), 136.0 (Ar-*C*), 135.4 (Ar-*C*), 132.5 (Ar-*C*H), 128.8 (Ar-*C*H), 125.0 (Ar-*C*H), 123.6 (Ar-*C*), 34.38 (*C*(CH_3)$_3$), 31.1 (C(*C*H$_3$)$_3$), 21.0 (Ar-*C*H$_3$), 19.0 (Ar-*C*H$_3$); **^{11}B NMR** (C_6D_6, 128.3 MHz): δ 26.1 (br); **UV-Vis** (CH_2Cl_2): λ = 369 nm (6316); **Fluorescence** (CH_2Cl_2): λ = 490 nm; **HRMS** (ESI): m/z calcd for $C_{58}H_{71}B_2N_4$: 845.5865 [(*M+H*)]$^+$; found : 845.5886.

Synthesis of compound 4a$^{\bullet+}$•[B(C_6F_5)$_4$]$^-$: A solution of [Ph_3C][B(C_6F_5)$_4$] (0.46 g, 0.50mmol) in CH_2Cl_2 (15 mL) was added dropwise to a CH_2Cl_2 solution of **4a** (0.37 g, 0.50 mmol) (10 ml) at -60 ^{o}C. The resultant intensely red solution was slowly warmed to room temperature and stirred overnight. After removal of the solvent, the residue was washed with hexane and dried under vacuum to afford **4a$^{\bullet+}$•[B(C_6F_5)$_4$]$^-$** as a deep red powder (0.59g, 84%). Single crystals suitable for X-ray diffraction studies were grown from a saturated CH_2Cl_2 and toluene solution at room temperature. **M. p.**: 212 ^{o}C (dec.); **UV-Vis** (CH_2Cl_2): 458 nm (ε = 1691 $M^{-1}cm^{-1}$), 368 nm (ε = 16430 $M^{-1}cm^{-1}$); **HRMS** (ESI): m/z calcd for $C_{50}H_{55}B_2N_4$: 733.4613 [(*M+H*)]$^+$; found :733.4631.

Compound $4b^{\bullet+}\bullet[B(C_6F_5)_4]^-$: reddish brown powder (87%). **M. p.**: 199 °C (dec); **UV-Vis** (CH_2Cl_2): 455 nm (ε = 2317 $M^{-1}cm^{-1}$), 368 nm (ε = 15980 $M^{-1}cm^{-1}$); **HRMS** (ESI): m/z calcd for $C_{50}H_{53}B_2N_4F_2$: 769.4424 [(*M+H*)]$^+$; found :769.4432.

Synthesis of compound 5a: HOTf (0.06 ml, 0.68 mmol) was added to a toluene solution (30 ml) of **4a** (498 mg, 0.68 mmol) at room temperature and the resulting mixture was stirred for 30mins. After removal of the solvent, the solid residue was washed with hexane and dried under vacuum to afford a white solid of **5a** (510 mg, 85%). Single crystals suitable for X-ray diffraction studies were grown from a saturated chloroform solution at room temperature. **M. p.**: 100.1 °C (dec); **^{1}H NMR** ($CDCl_3$, 400 MHz, 298 K): *δ* 7.71 (s, 1H, NC*H*N), 7.26 (t, *J* = 7.6 Hz, 2H, Ar-*H*), 7.00 (t, *J* = 7.6 Hz, 4H, Ar-*H*), 6.84 (d, *J* = 8.0 Hz, 4H, Ar-*H*), 6.69 (s, 2H, Ar-*H*), 6.62 (s, 2H, Ar-*H*), 6.61 (s, 2H, Ar-*H*), 6.59 (s, 2H, Ar-*H*), 2.33 (s, 6H, Ar-C*H*$_3$), 2.20 (s, 6H, Ar-C*H*$_3$), 2.18 (s, 6H, Ar-C*H*$_3$), 2.17 (s, 6H, Ar-C*H*$_3$), 2.11 (s, 6H, Ar-C*H*$_3$), 2.06 (s, 6H, Ar-C*H*$_3$); **^{13}C{^{1}H} NMR** ($CDCl_3$, 100 MHz, 298 K): *δ* 187.4 (N*C*N), 139.4 (Ar-*C*), 137.3 (Ar-*C*), 135.4 (Ar-*C*), 134.4 (Ar-*C*), 133.4 (Ar-*C*), 133.1 (Ar-*C*H), 132.4 (Ar-*C*), 132.3 (Ar-*C*H), 129.8 (Ar-*C*H), 129.7 (Ar-*C*), 129.6 (Ar-*C*H), 129.2 (Ar-*C*H), 129.1 (Ar-*C*H), 128.3 (Ar-*C*H), 82.2 (N*C*HN), 20.84 (Ar-*C*H$_3$), 20.80 (Ar-*C*H$_3$), 19.8 (Ar-*C*H$_3$), 19.5 (Ar-*C*H$_3$), 19.2 (Ar-*C*H$_3$), 19.0 (Ar-*C*H$_3$); **^{11}B NMR** ($CDCl_3$, 128.3 MHz): *δ* 38.1 (br); **^{19}F{^{1}H} NMR** ($CDCl_3$, 376 MHz): *δ* -78.0 (C*F*$_3$); **HRMS** (ESI): m/z calcd for $C_{50}H_{55}B_2N_4$: 733.4613 [(*M-OTf*)]$^+$; found : 733.4623.

Compound 5b: white powder (89%). **M. p.**: 95.0 °C (dec); **^{1}H NMR** ($CDCl_3$, 400 MHz, 298 K): *δ* 7.70 (s, 1H, NC*H*N), 6.86-6.83 (m, 4H, Ar-*H*), 6.73-6.69 (m, 6H, Ar-*H*), 6.63 (s, 4H, Ar-*H*), 6.60 (s, 2H, Ar-*H*), 2.32 (s, 6H, Ar-C*H*$_3$), 2.18 (s, 18H, Ar-C*H*$_3$), 2.09 (s, 6H, Ar-C*H*$_3$), 2.05 (s, 6H, Ar-C*H*$_3$); **^{13}C{^{1}H} NMR** ($CDCl_3$, 100 MHz, 298 K): *δ* 187.6 (N*C*N), 165.2 (d, $^1J_{CF}$ = 254 Hz, Ar-*C*F), 139.6 (Ar-*C*), 137.6 (Ar-*C*), 135.8 (d, $^3J_{CF}$ = 9 Hz, Ar-*C*CCF), 135.4 (Ar-*C*), 134.6 (Ar-*C*), 133.6 (Ar-*C*), 133.0 (Ar-*C*), 132.3 (Ar-*C*), 130.0 (Ar-*C*), 129.8 (Ar-*C*H), 129.6 (Ar-*C*), 129.2 (Ar-*C*H), 129.1 (Ar-*C*H), 115.9 (d, $^2J_{CF}$ = 21 Hz, Ar-*C*CF), 82.5 (N*C*HN), 20.9 (Ar-*C*H$_3$), 20.8 (Ar-*C*H$_3$), 19.9 (Ar-*C*H$_3$), 19.5 (Ar-*C*H$_3$), 19.2 (Ar-*C*H$_3$), 19.0 (Ar-*C*H$_3$); **^{11}B NMR** ($CDCl_3$, 128.3 MHz): *δ* 39.3 (br); **^{19}F{^{1}H} NMR** ($CDCl_3$, 376 MHz): *δ* -78.1 (C*F*$_3$), -104.5 (Ar-*F*); **HRMS** (ESI): m/z calcd for $C_{50}H_{53}B_2N_4F_2$: 769.4424 [(*M-OTf*)]$^+$; found: 769.4441.

Compound 5c: white powder (92%). **M. p.**: 97.6 °C (dec); **^{1}H NMR** ($CDCl_3$, 400 MHz, 298 K): *δ* 7.47 (s, 1H, NC*H*N), 7.01 (d, *J* = 8.0 Hz, 4H, Ar-*H*), 6.77 (d, *J* = 8.0 Hz, 4H, Ar-*H*), 6.74 (s, 2H, Ar-*H*), 6.67 (s, 2H, Ar-*H*), 6.65 (s, 2H, Ar-*H*), 6.63(s, 2H, Ar-*H*), 2.32 (s, 6H, Ar-C*H*$_3$), 2.22 (s, 6H, Ar-C*H*$_3$), 2.21 (s, 6H, Ar-C*H*$_3$), 2.16 (s, 12H, Ar-C*H*$_3$), 2.07 (s, 6H, Ar-C*H*$_3$), 1.14 (s, 18H, C(C*H*$_3$)$_3$); **^{13}C{^{1}H} NMR** ($CDCl_3$, 100 MHz, 298 K): *δ* 187.1 (N*C*N), 156.2 (Ar-*C*), 139.5 (Ar-*C*), 137.4 (Ar-*C*), 135.7 (Ar-*C*), 134.2 (Ar-*C*), 133.5 (Ar-*C*), 133.3 (Ar-*C*H), 133.1 (Ar-*C*), 132.6 (Ar-*C*), 130.1 (Ar-*C*), 130.0 (Ar-*C*H), 129.7 (Ar-*C*H), 129.3(Ar-*C*H), 125.5 (Ar-*C*H), 81.9 (N*C*HN), 35.0 (*C*(CH$_3$)$_3$), 30.9 (C(*C*H$_3$)$_3$), 20.94 (Ar-*C*H$_3$), 20.88 (Ar-*C*H$_3$), 19.8 (Ar-*C*H$_3$), 19.6 (Ar-*C*H$_3$), 19.3 (Ar-*C*H$_3$), 19.0(Ar-*C*H$_3$); **^{11}B NMR** ($CDCl_3$, 128.3 MHz): *δ* 29.0 (br); **^{19}F{^{1}H}** NMR ($CDCl_3$, 376 MHz): *δ* -78.1 (C*F*$_3$); **HRMS** (ESI): m/z calcd for $C_{58}H_{71}B_2N_4$: 845.5865 [(*M-OTf*)]$^+$; found : 845.5877.

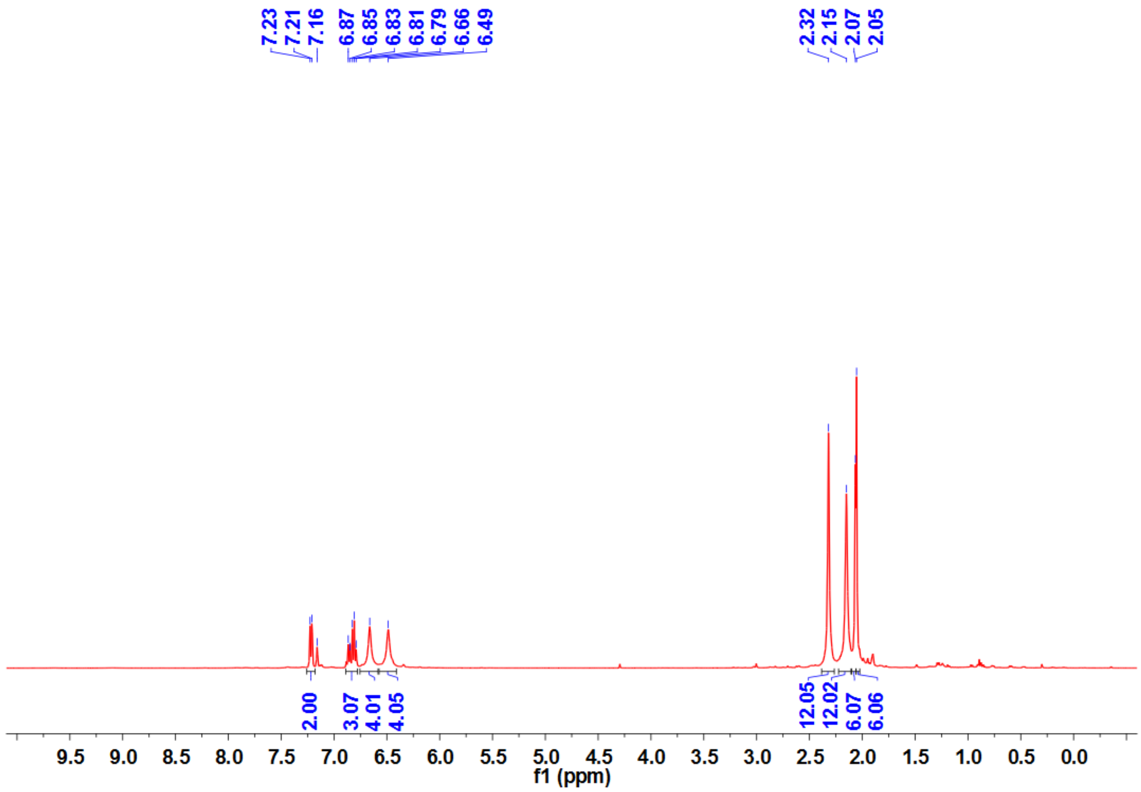

Figure S1. ^{1}H NMR spectrum of **2a** in C_6D_6.

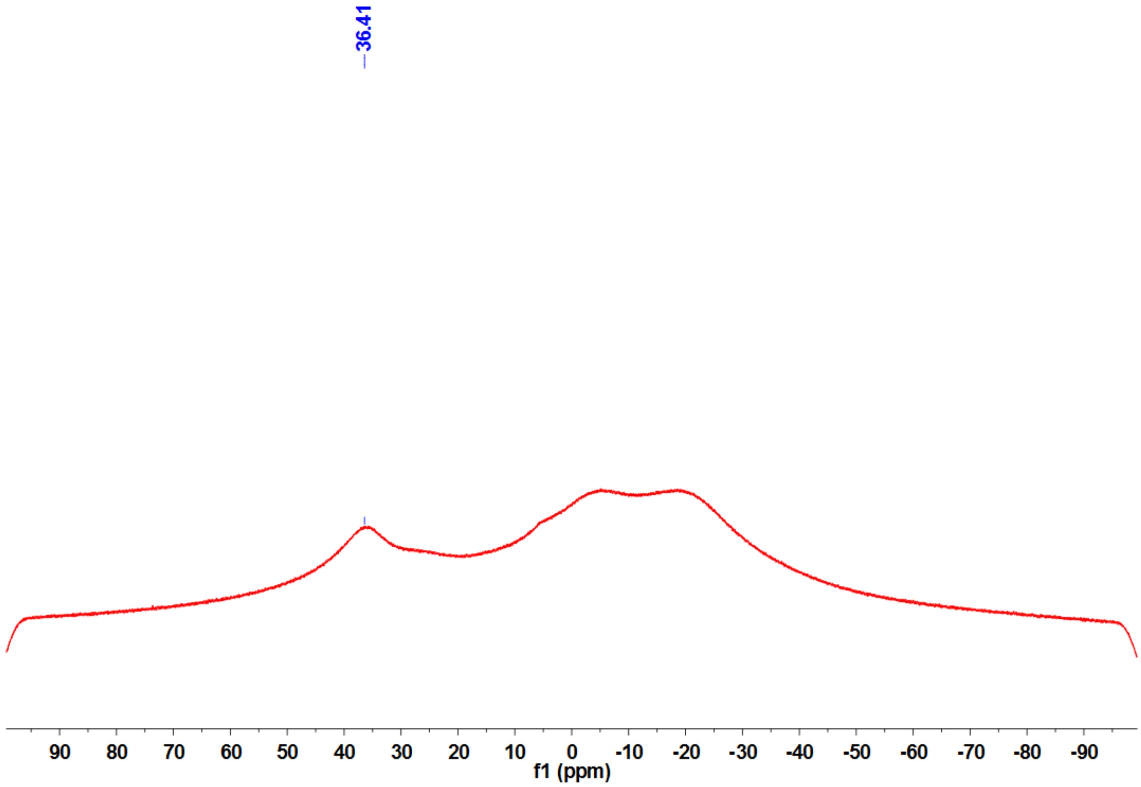

Figure S2. ^{11}B NMR spectrum of **2a** in C_6D_6.

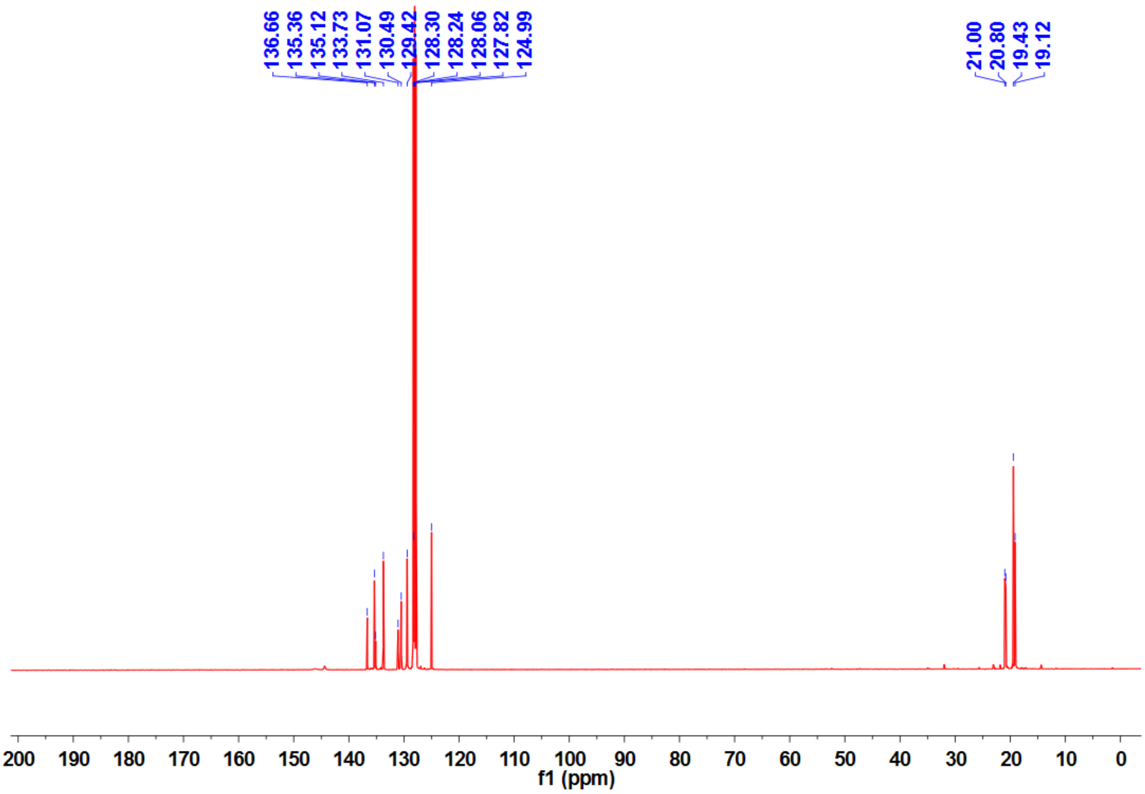

Figure S3. $^{13}C\{^{1}H\}$ NMR spectrum of **2a** in C_6D_6.

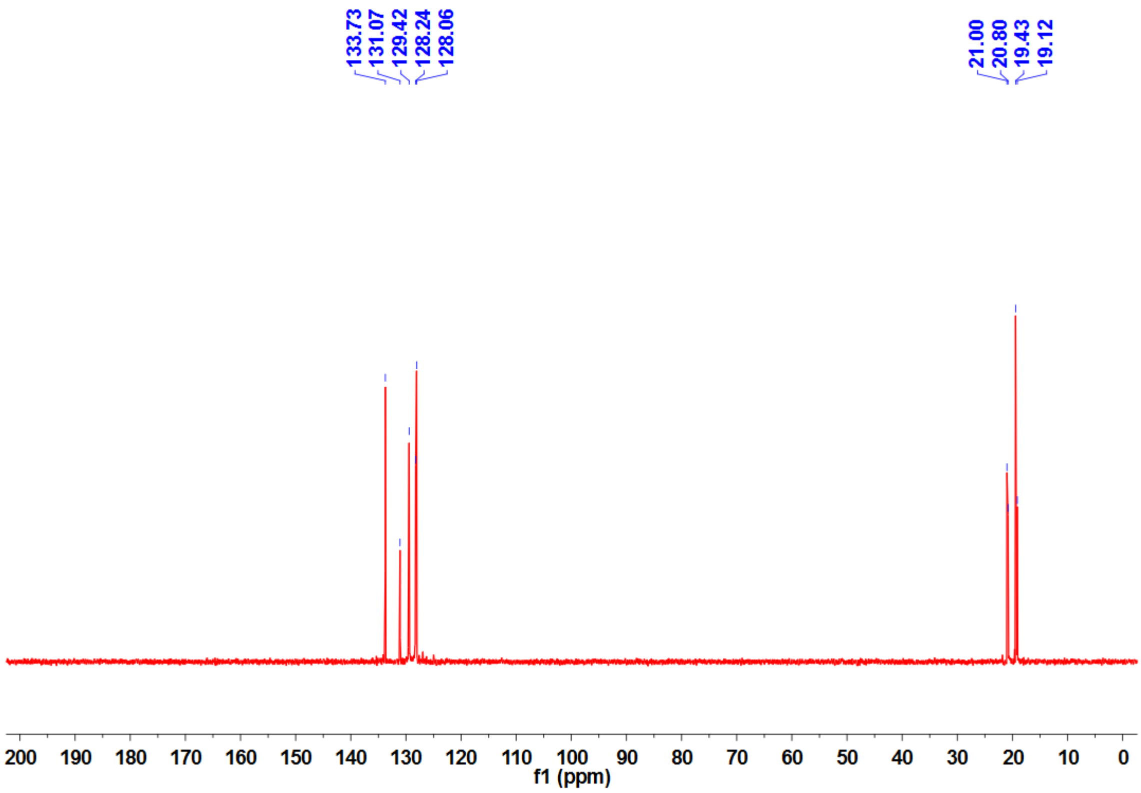

Figure S4. ^{13}C (DEPT135) NMR spectrum of **2a** in C_6D_6.

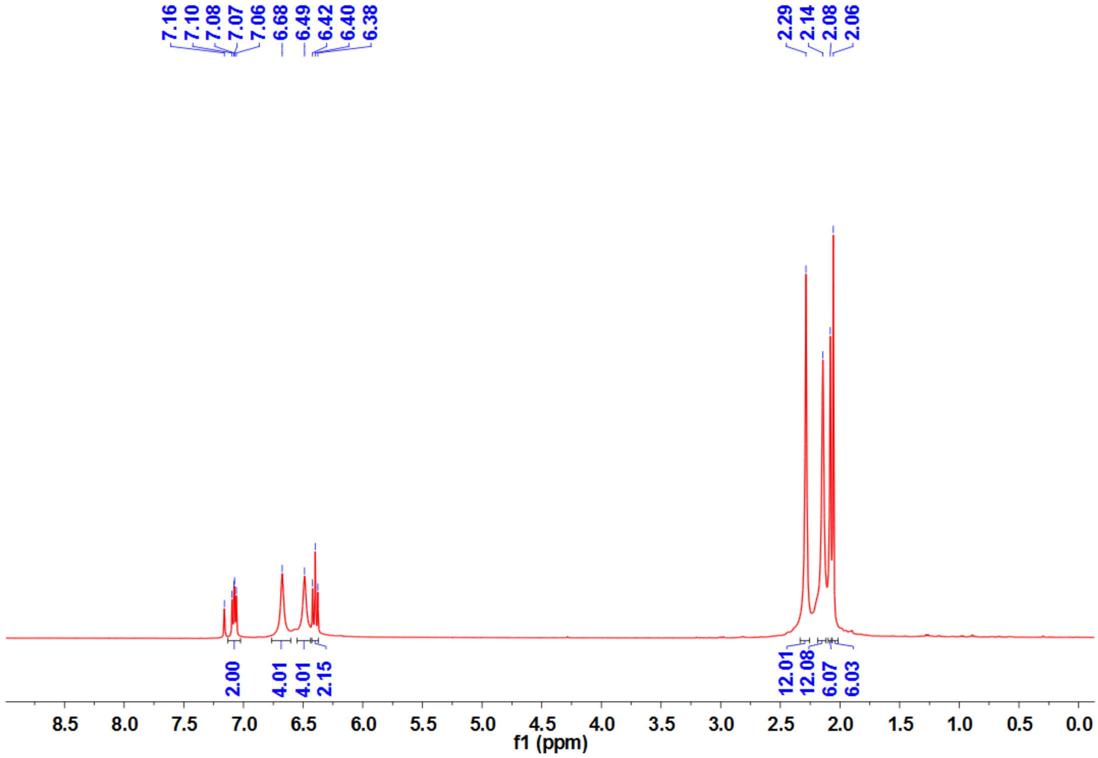

Figure S5. ^{1}H NMR spectrum of **2b** in C_6D_6.

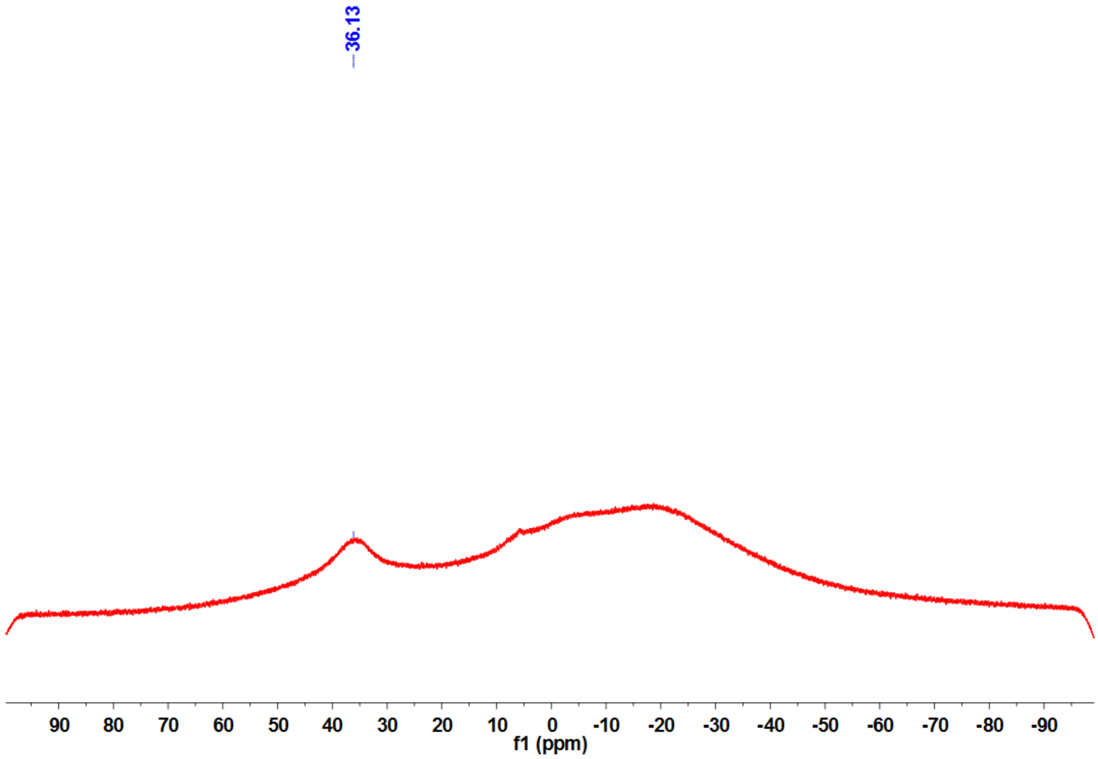

Figure S6. ^{11}B NMR spectrum of **2b** in C_6D_6.

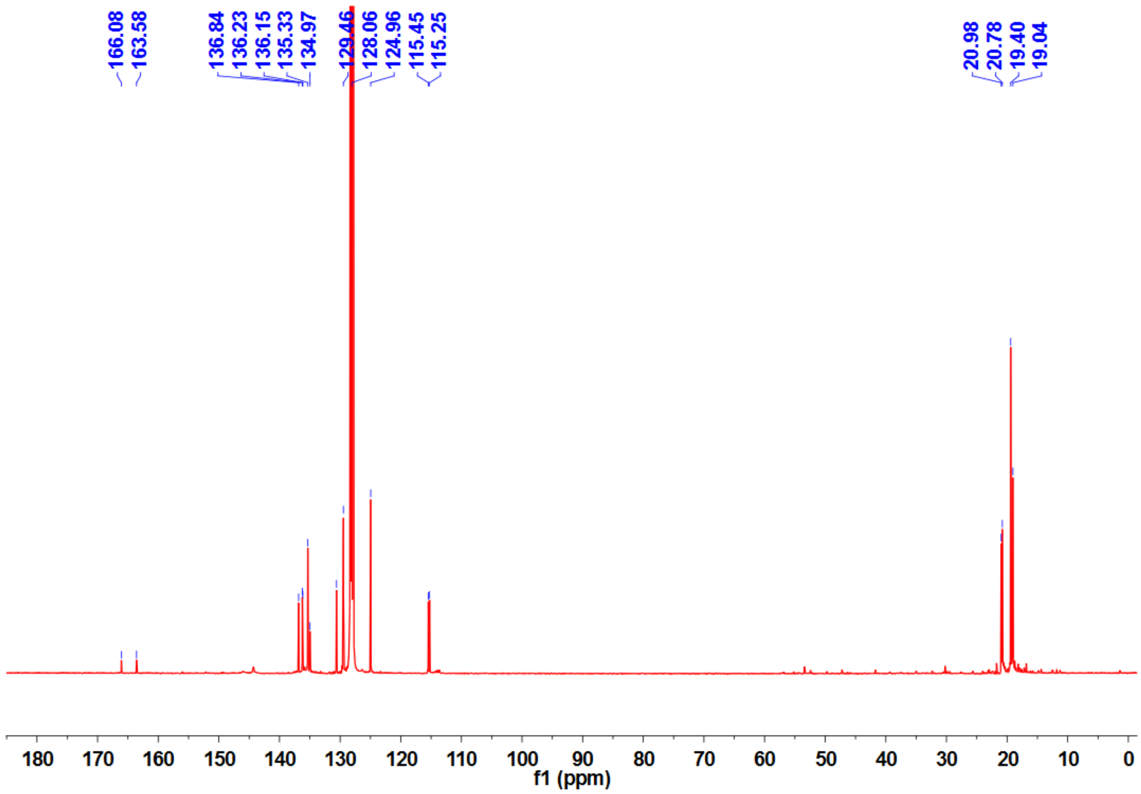

Figure S7. $^{13}C\{^{1}H\}$ NMR spectrum of **2b** in C_6D_6.

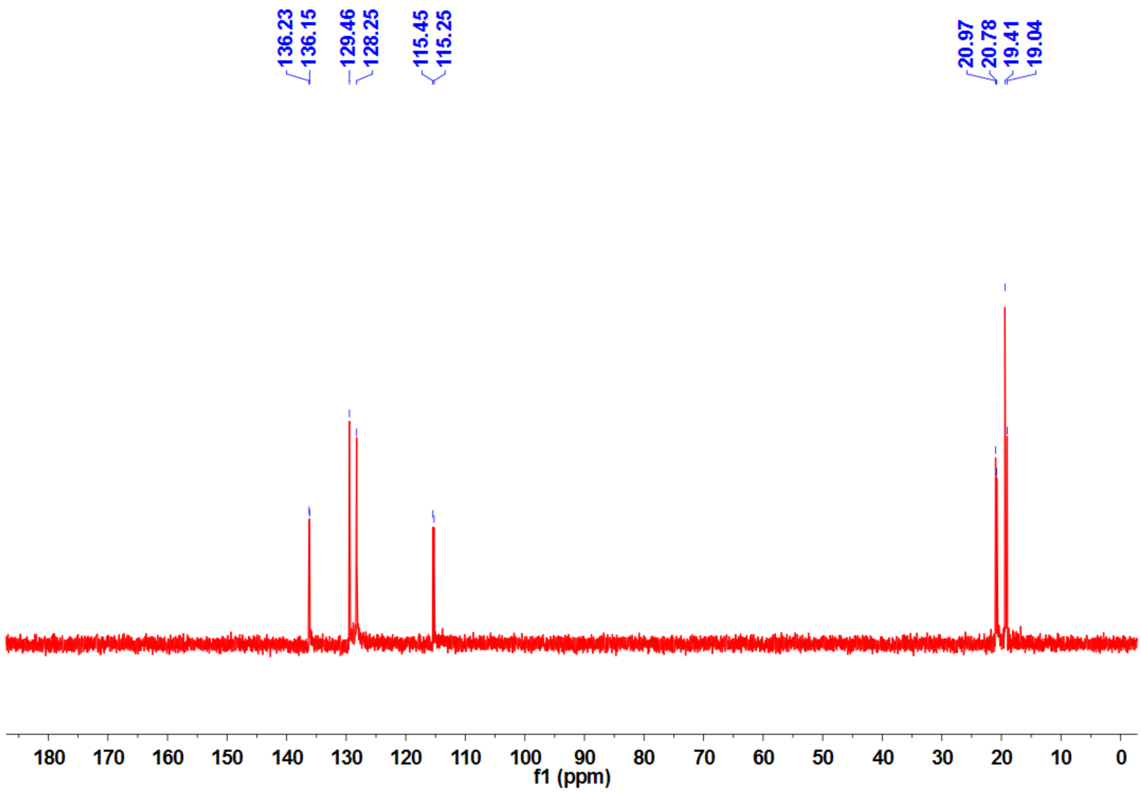

Figure S8. ^{13}C (DEPT135) NMR spectrum of **2b** in C_6D_6.

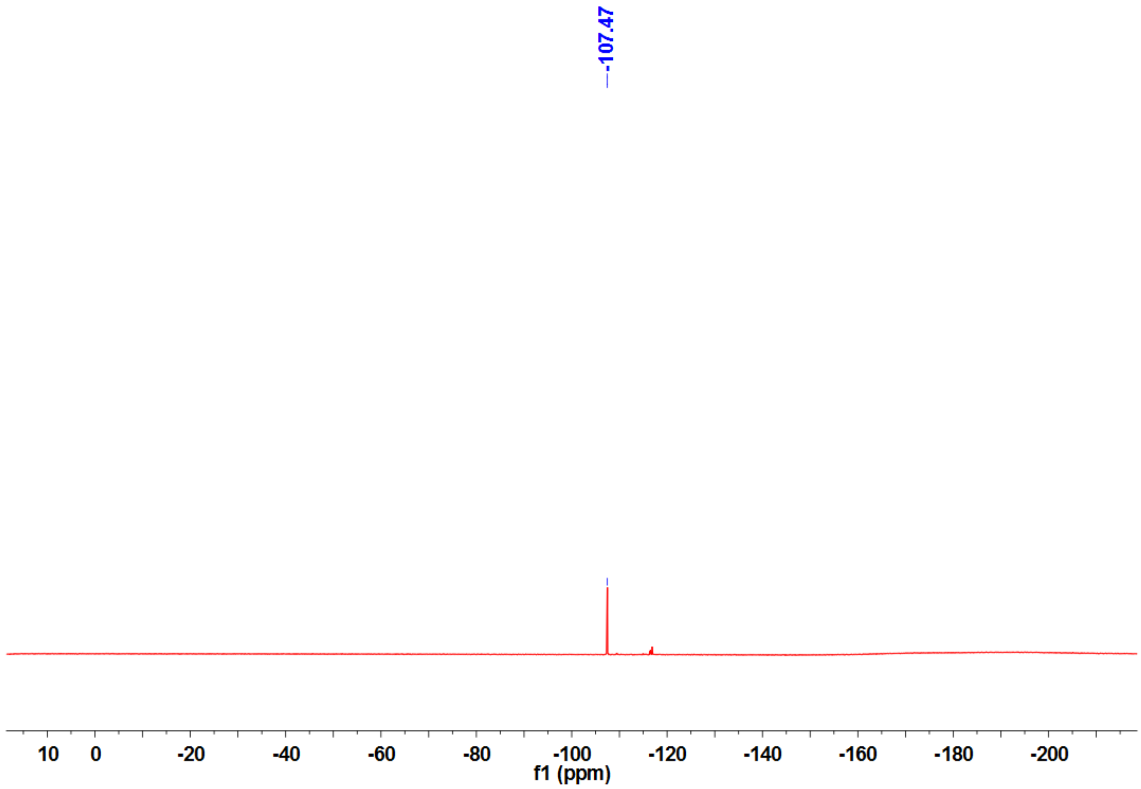

Figure S9. ^{19}F NMR spectrum of **2b** in C_6D_6.

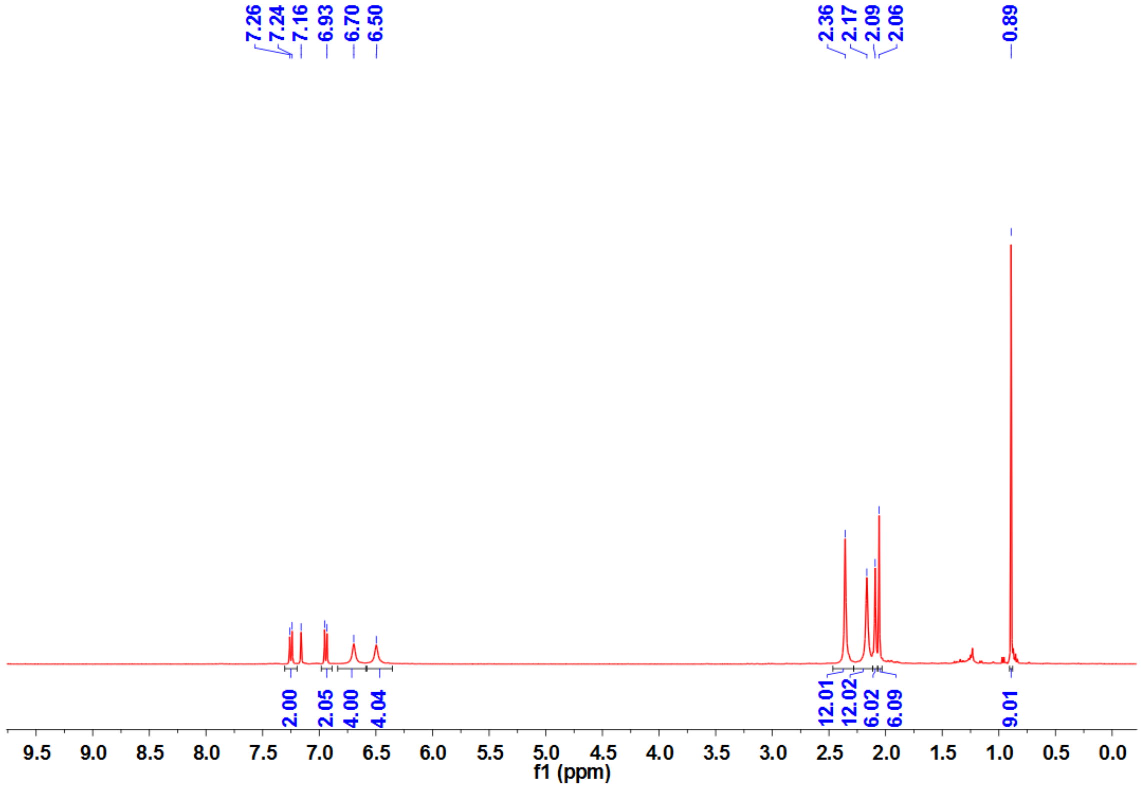

Figure S10. ^{1}H NMR spectrum of **2c** in C_6D_6.

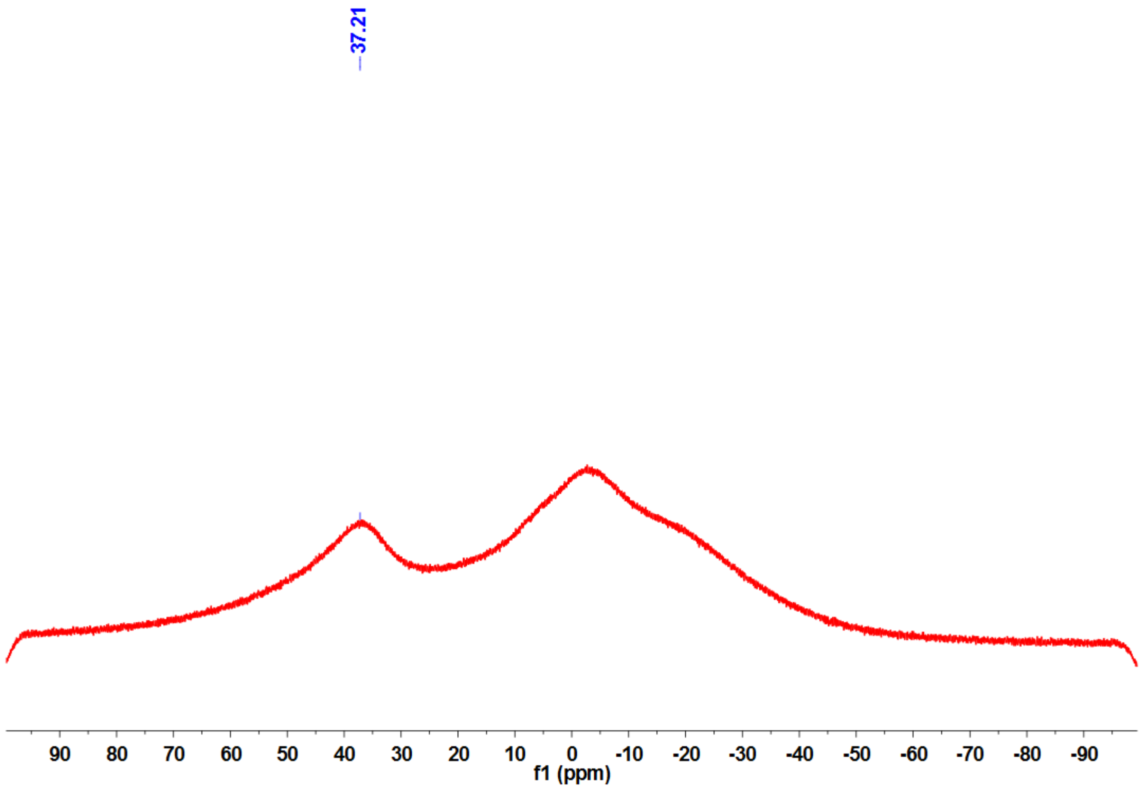

Figure S11. ^{11}B NMR spectrum of **2c** in C_6D_6.

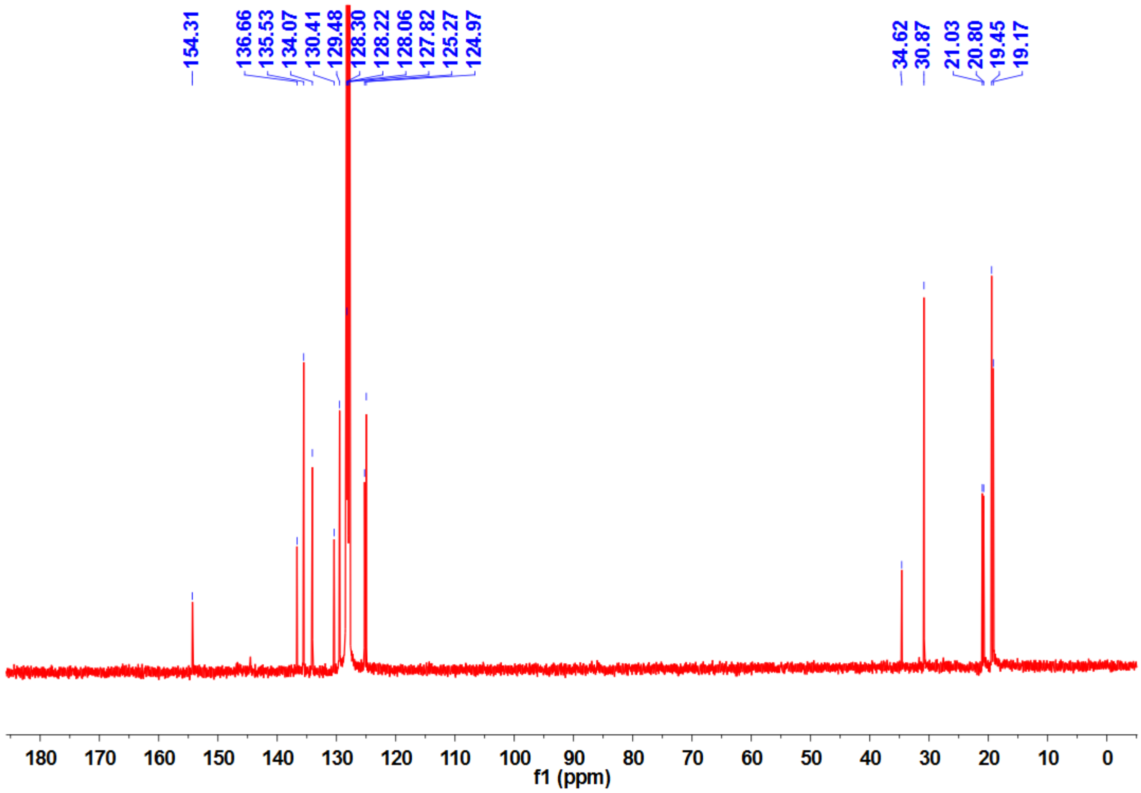

Figure S12. ^{13}C{^{1}H} NMR spectrum of **2c** in C_6D_6.

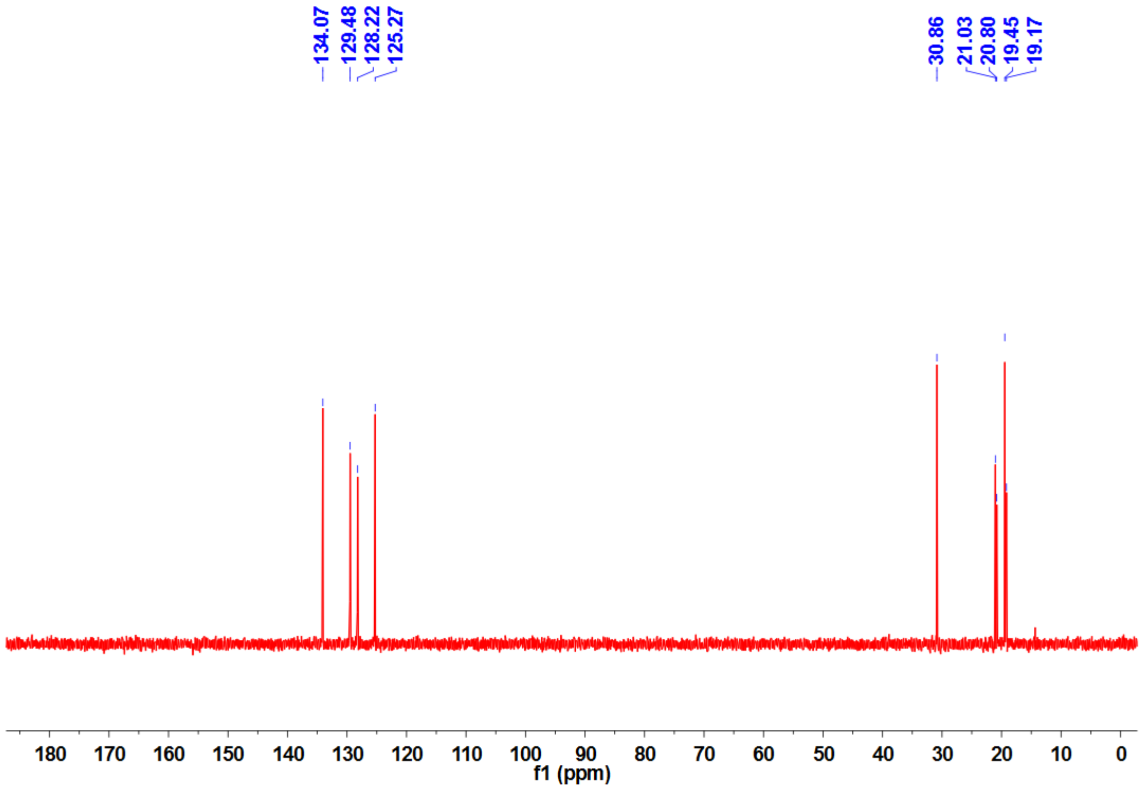

Figure S13. ^{13}C (DEPT135) NMR spectrum of **2c** in C_6D_6.

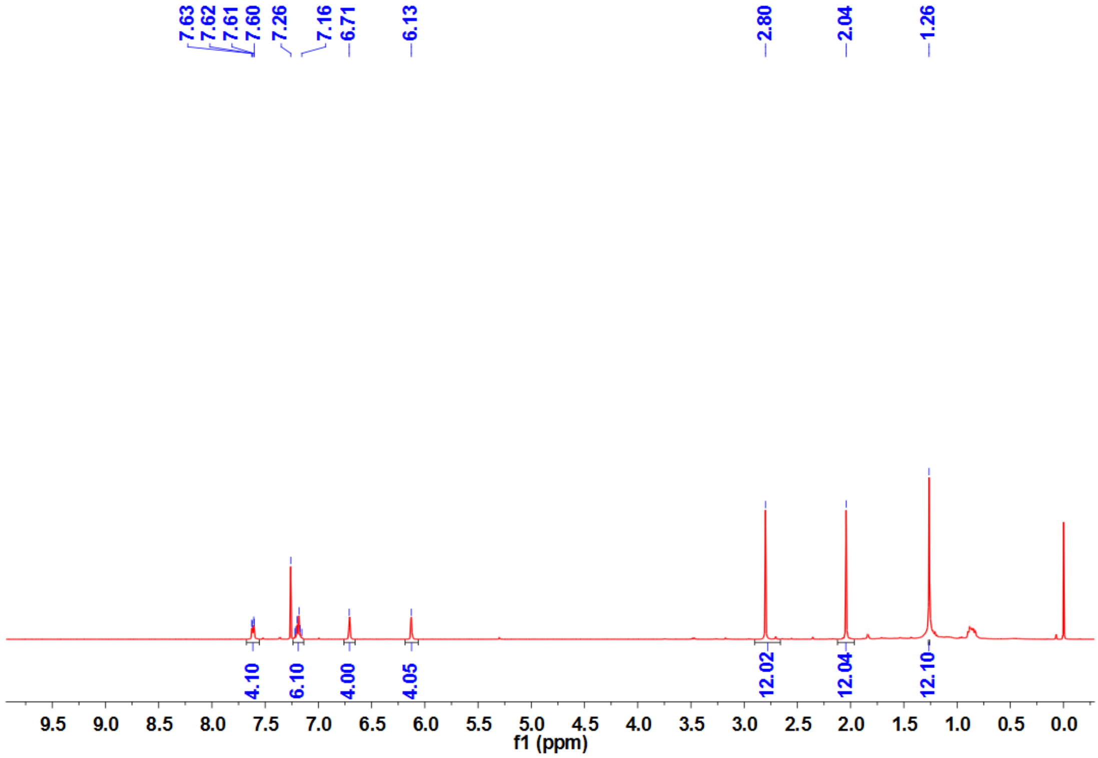

Figure S14. ^{1}H NMR spectrum of **3a** in $CDCl_3$.

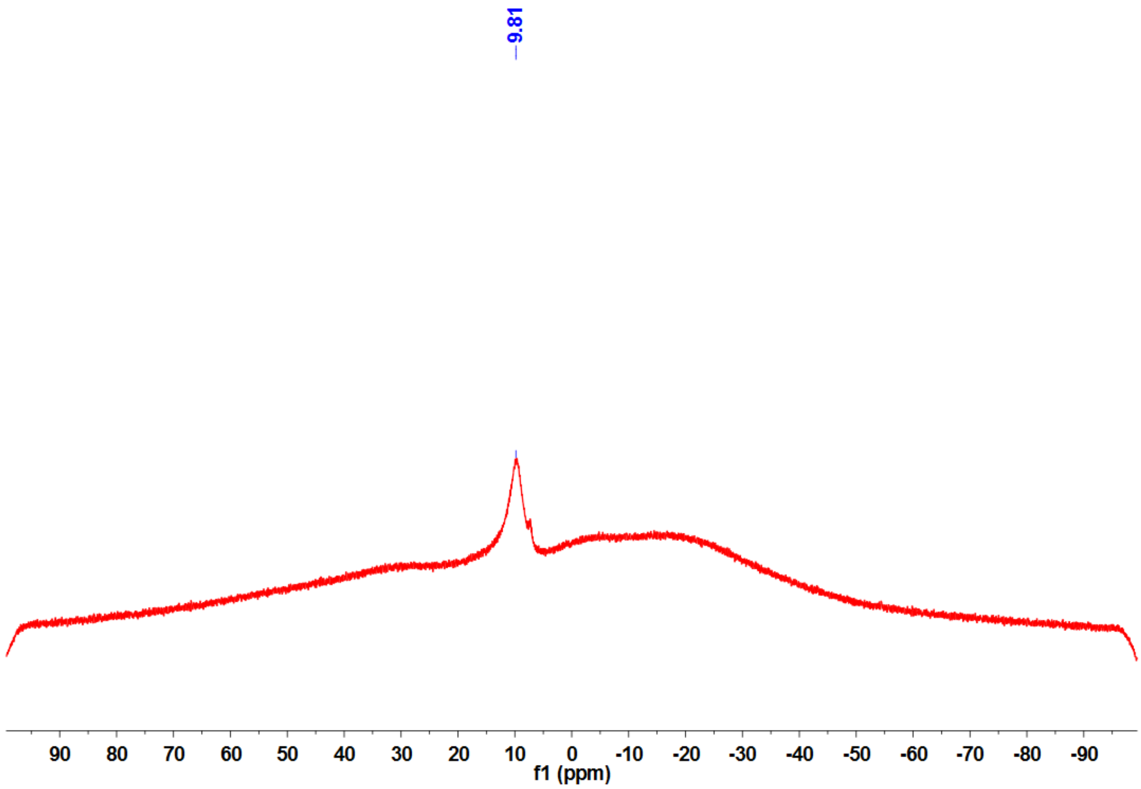

Figure S15. ^{11}B NMR spectrum of **3a** in $CDCl_3$.

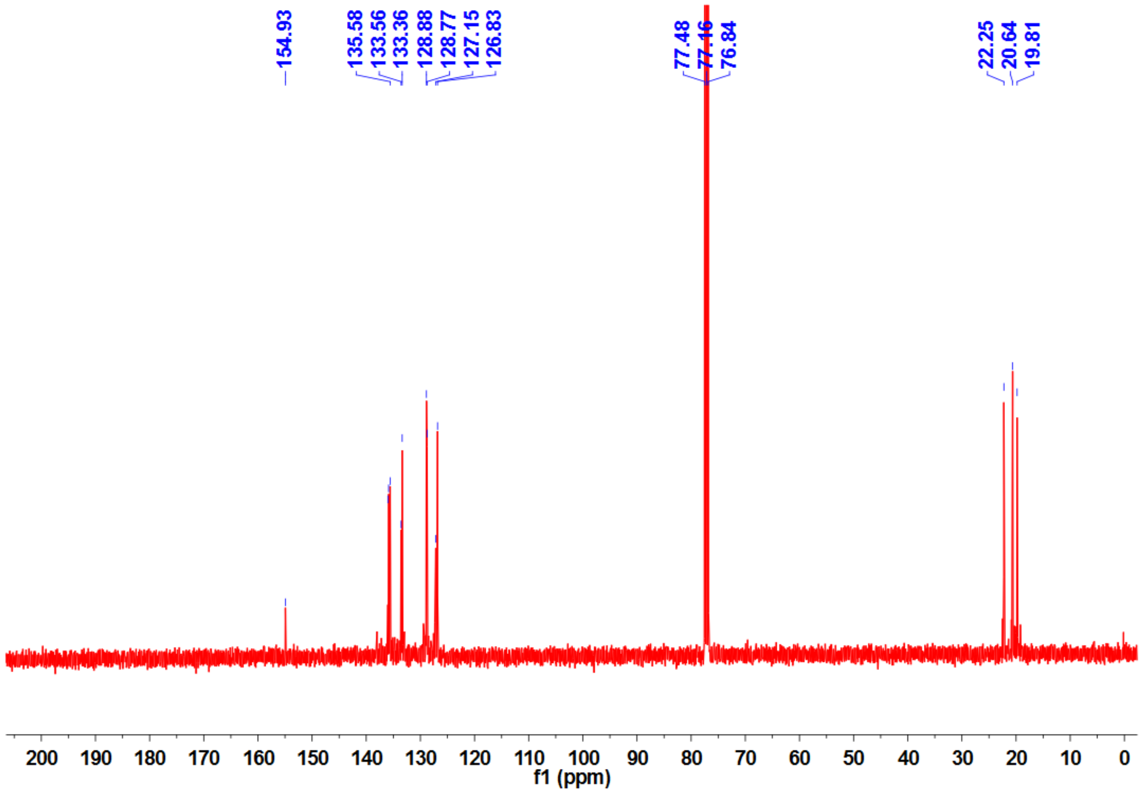

Figure S16. $^{13}C\{^{1}H\}$ NMR spectrum of **3a** in $CDCl_3$.

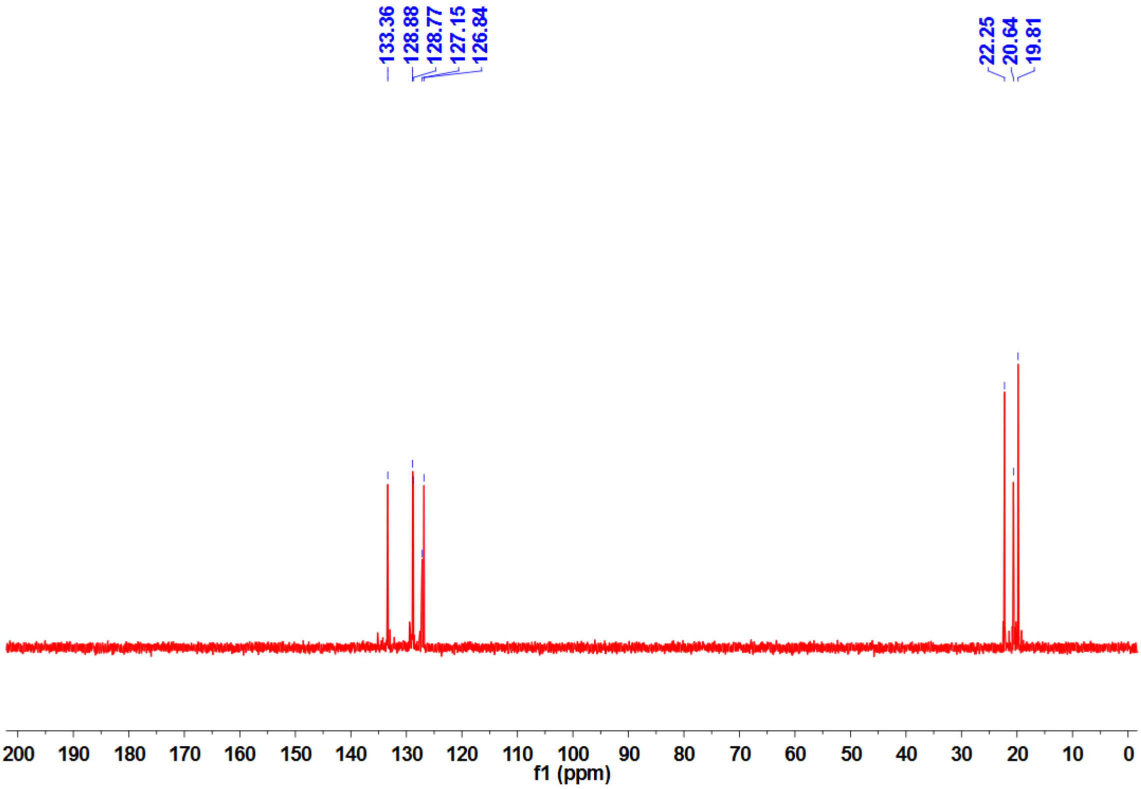

Figure S17. ^{13}C (DEPT135) NMR spectrum of **3a** in $CDCl_3$.

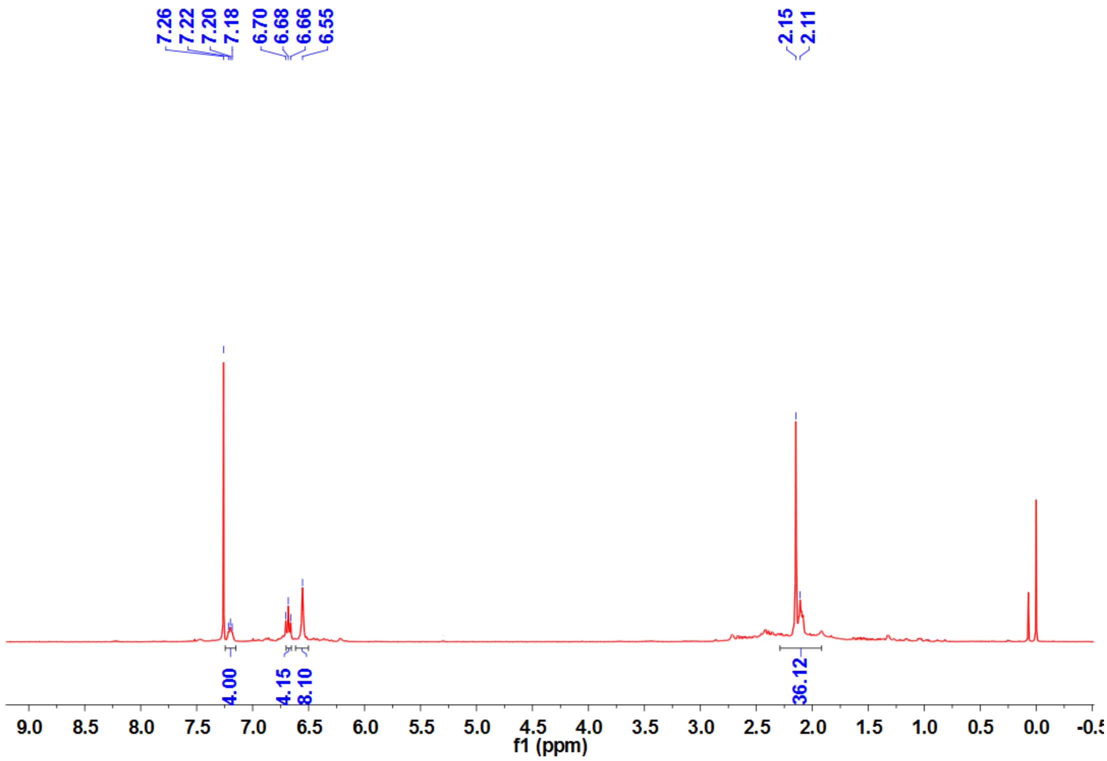

Figure S18. ^{1}H NMR spectrum of **3b** in $CDCl_3$.

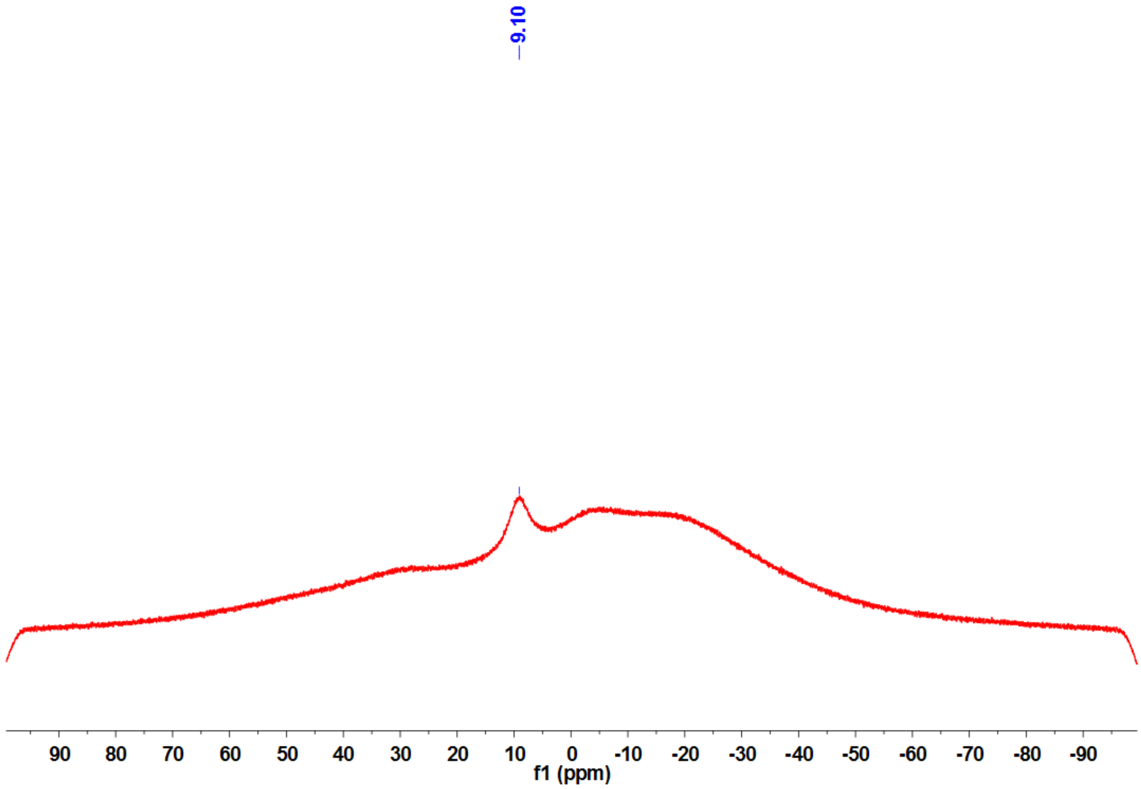

Figure S19. ^{11}B NMR spectrum of **3b** in $CDCl_3$.

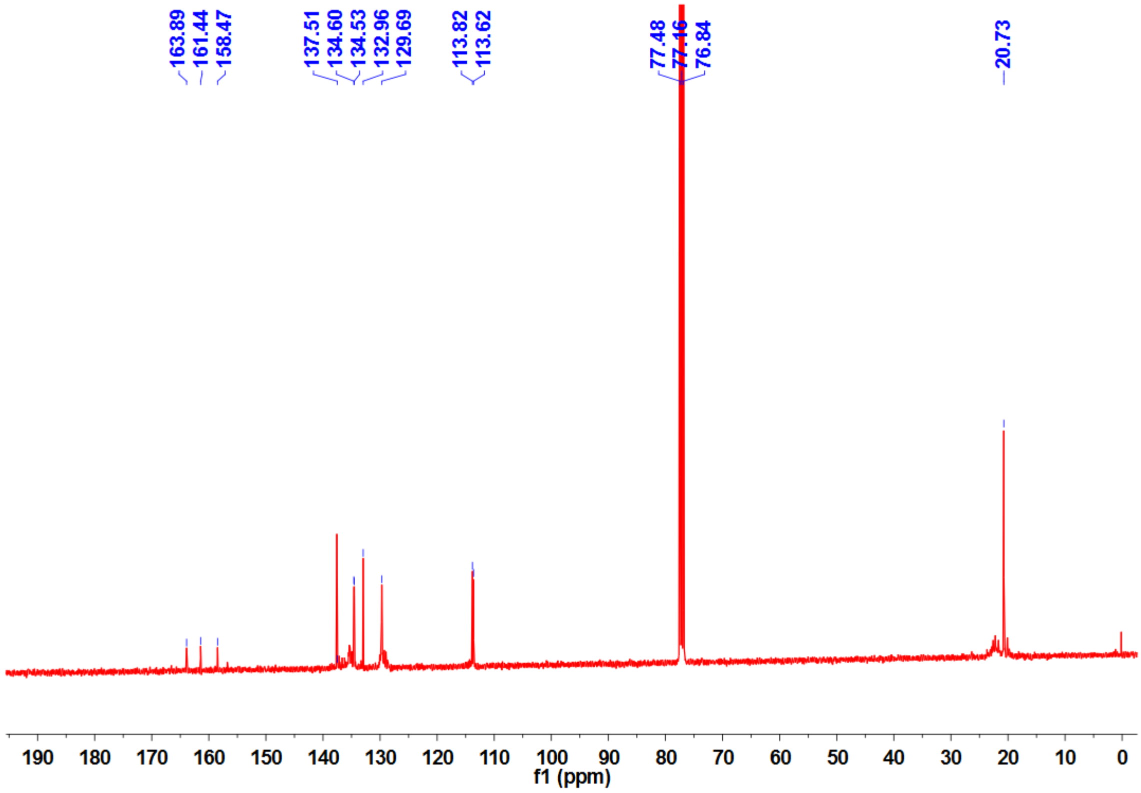

Figure S20. $^{13}C\{^1H\}$ NMR spectrum of **3b** in $CDCl_3$.

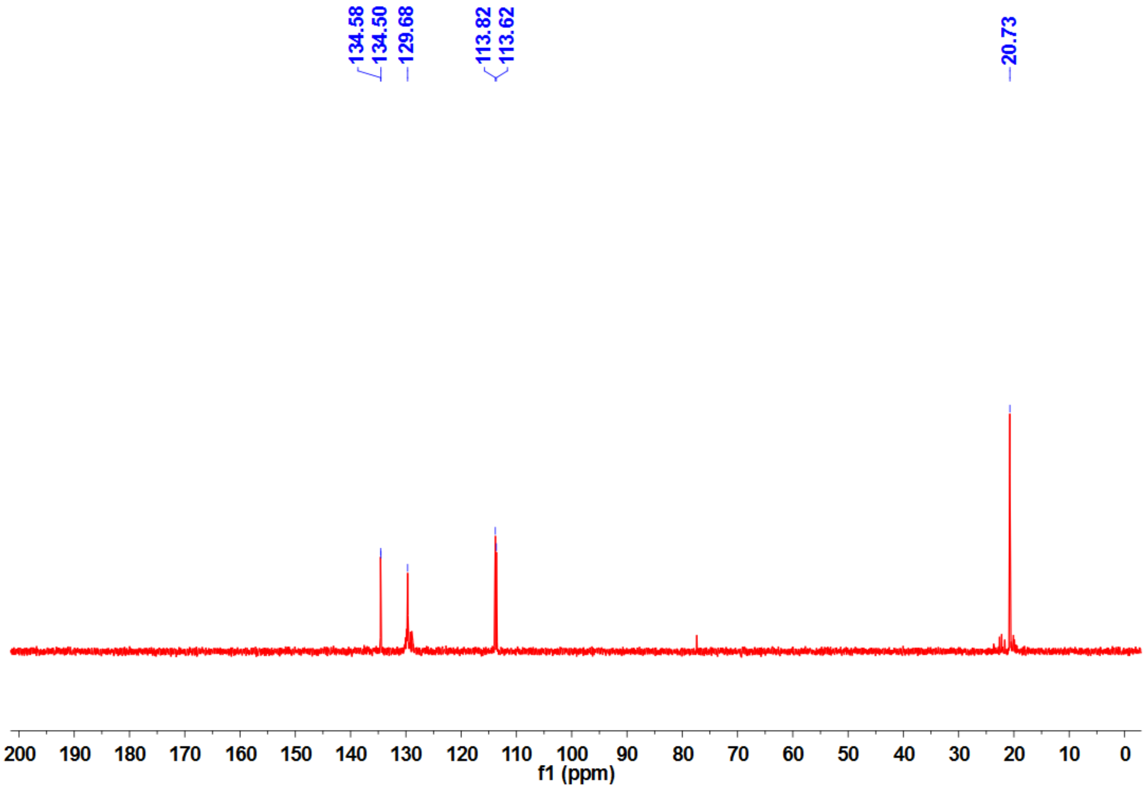

Figure S21. ^{13}C (DEPT135) NMR spectrum of **3b** in $CDCl_3$.

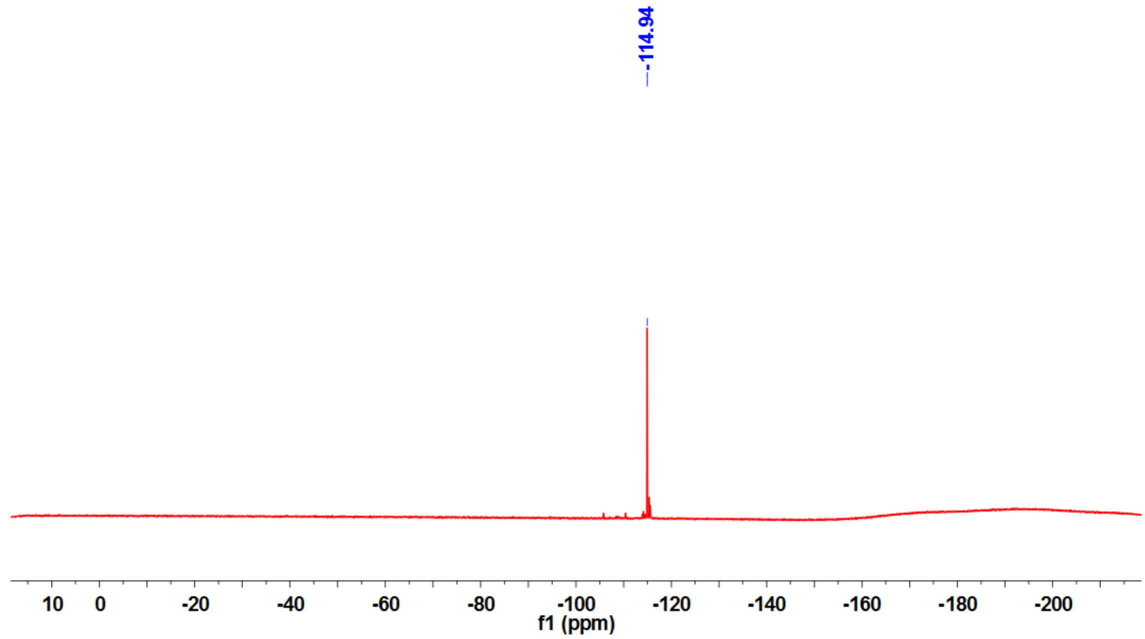

Figure S22. $^{19}F\{^1H\}$ NMR spectrum of **3b** in $CDCl_3$.

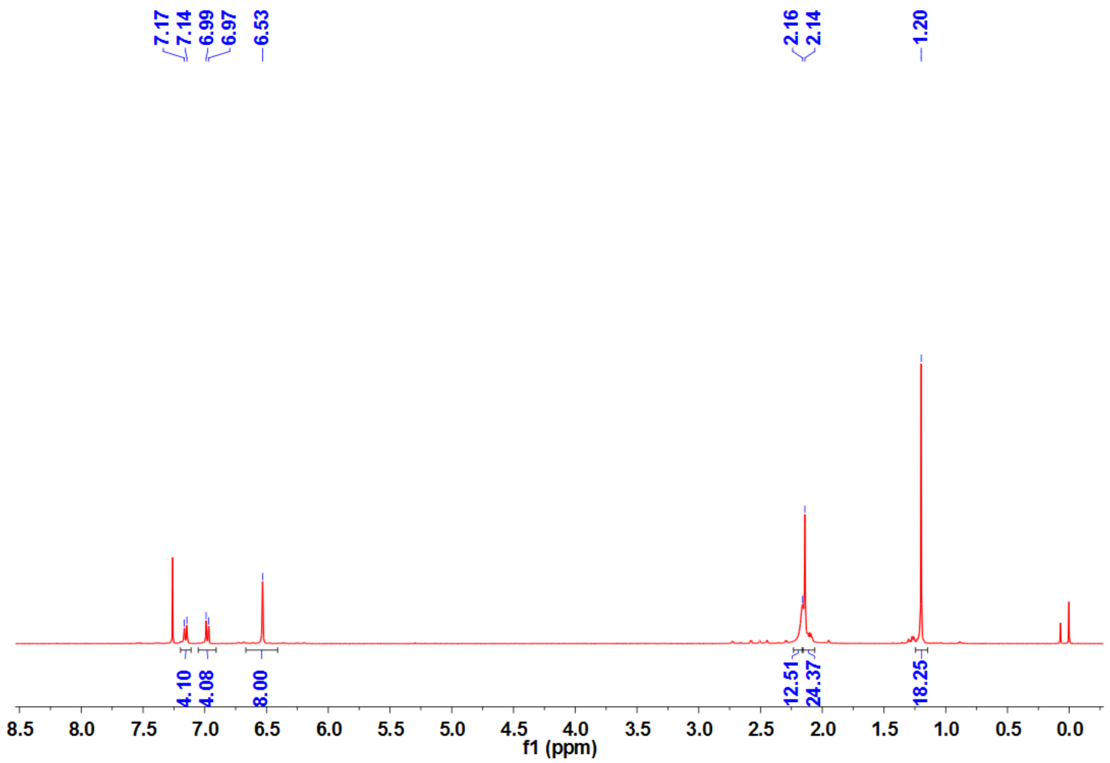

Figure S23. ^{1}H NMR spectrum of **3c** in $CDCl_3$.

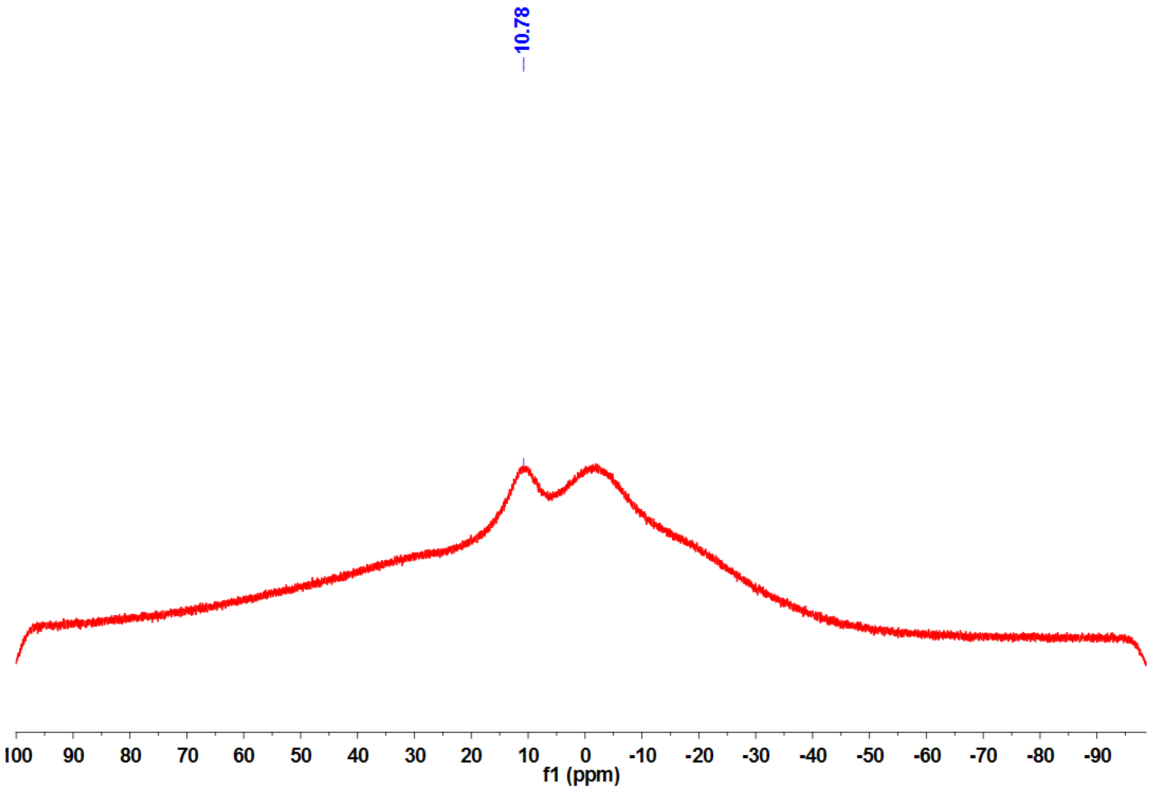

Figure S24. ^{11}B NMR spectrum of **3c** in $CDCl_3$.

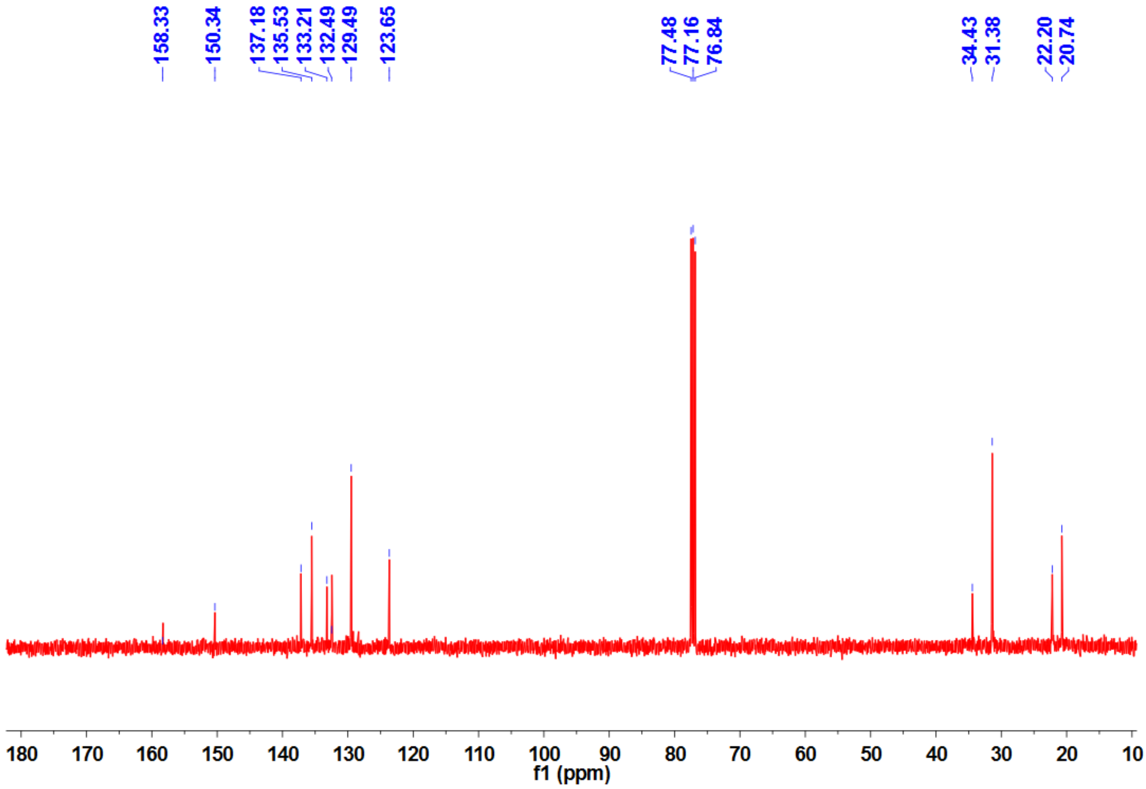

Figure S25. $^{13}C\{^{1}H\}$ NMR spectrum of **3c** in $CDCl_3$.

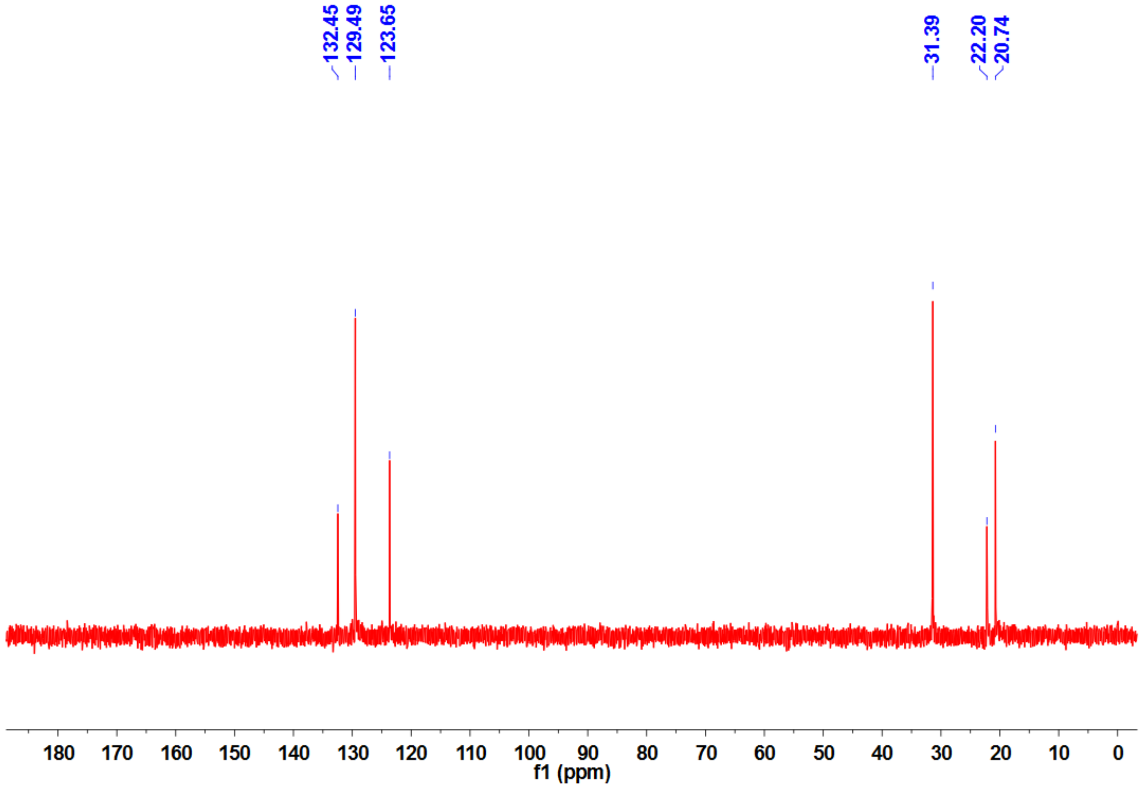

Figure S26. ^{13}C (DEPT135) NMR spectrum of **3c** in $CDCl_3$.

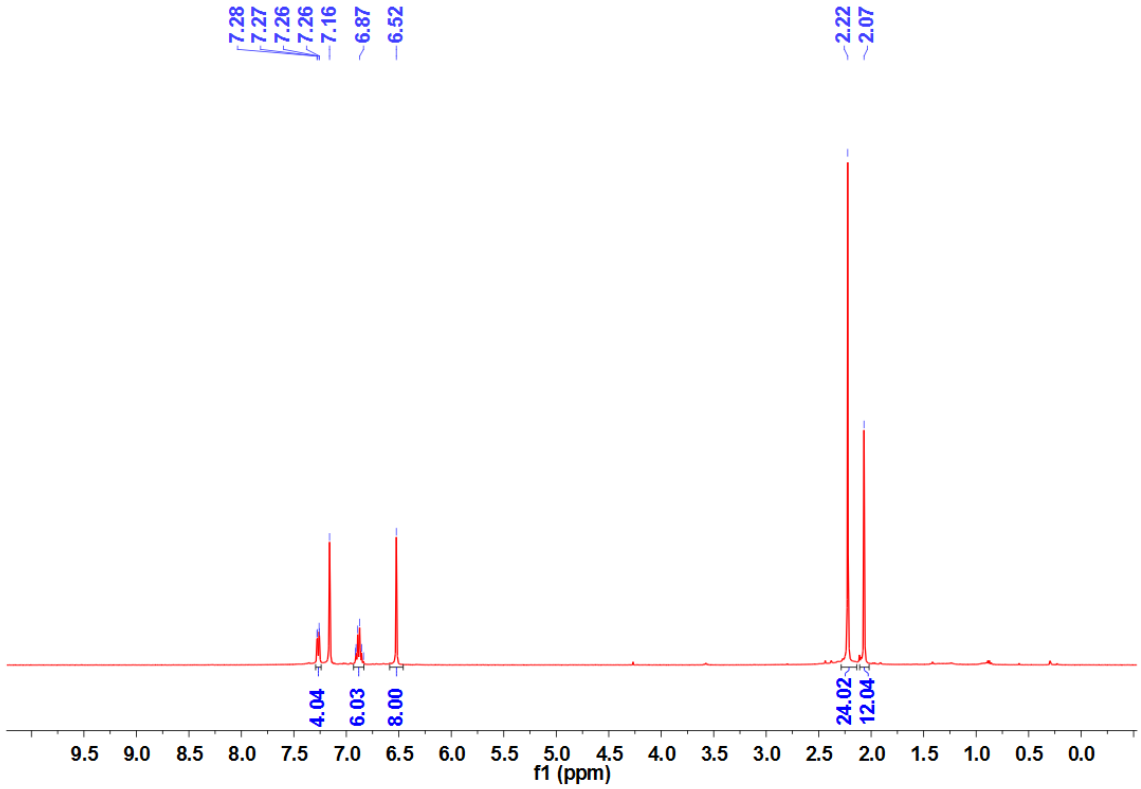

Figure S27. ^{1}H NMR spectrum of **4a** in C_6D_6.

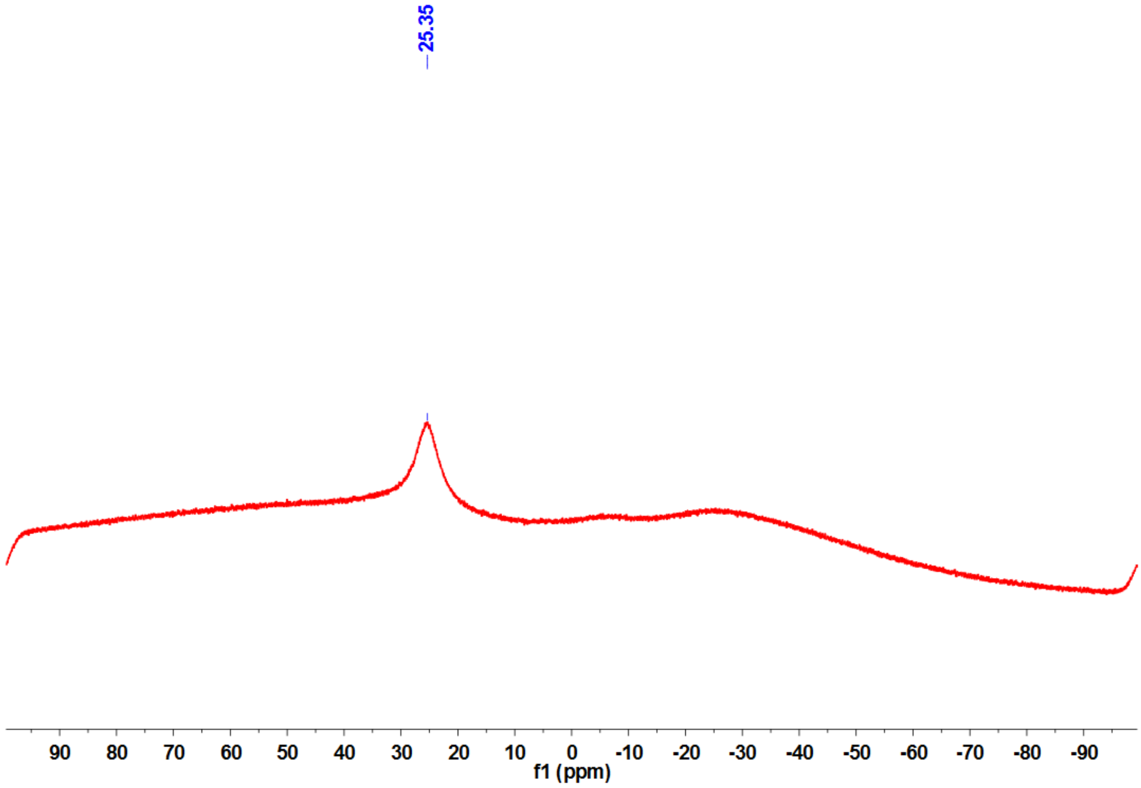

Figure S28. ^{11}B NMR spectrum of **4a** in $CDCl_3$.

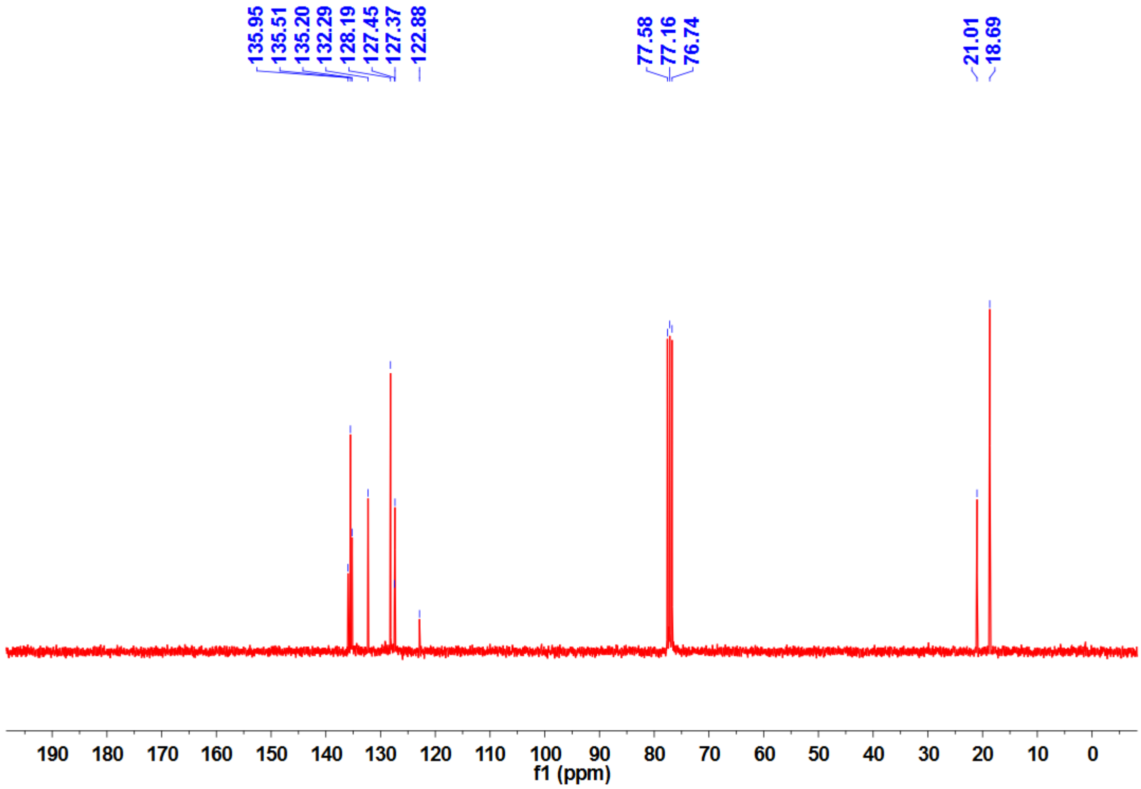

Figure S29. $^{13}C\{^1H\}$ NMR spectrum of **4a** in $CDCl_3$.

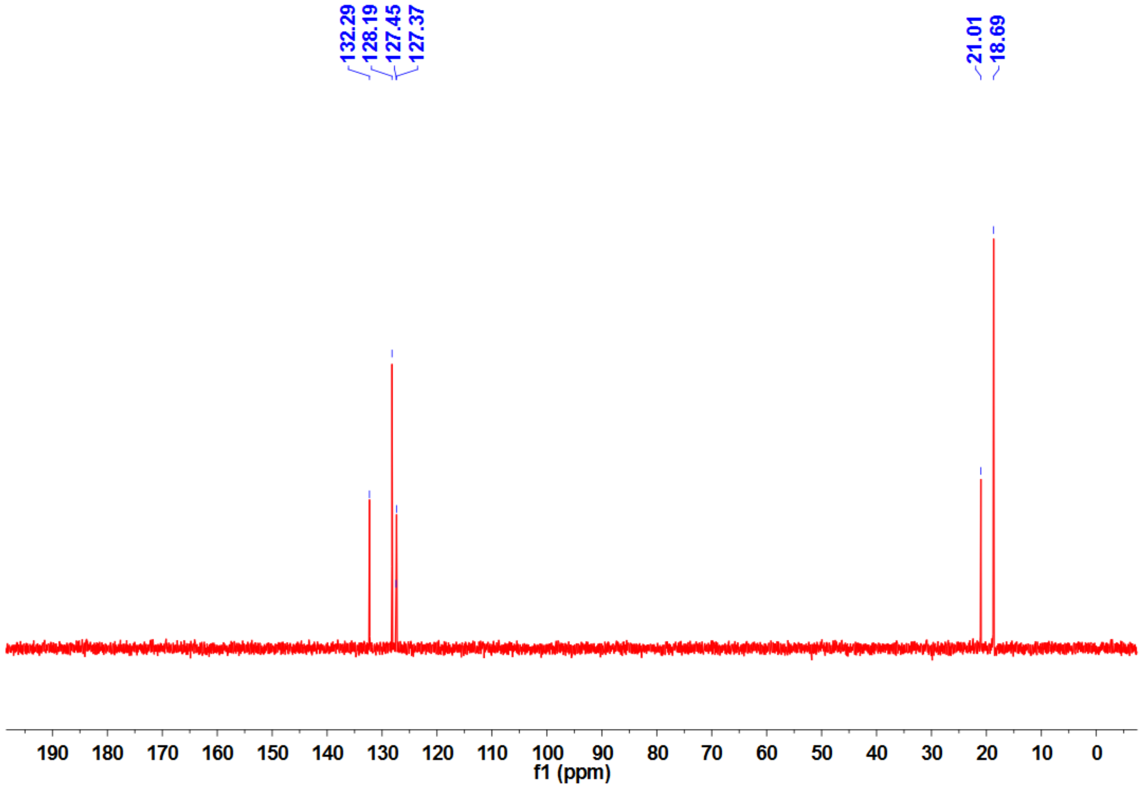

Figure S30. ^{13}C (DEPT135) NMR spectrum of **4a** in $CDCl_3$.

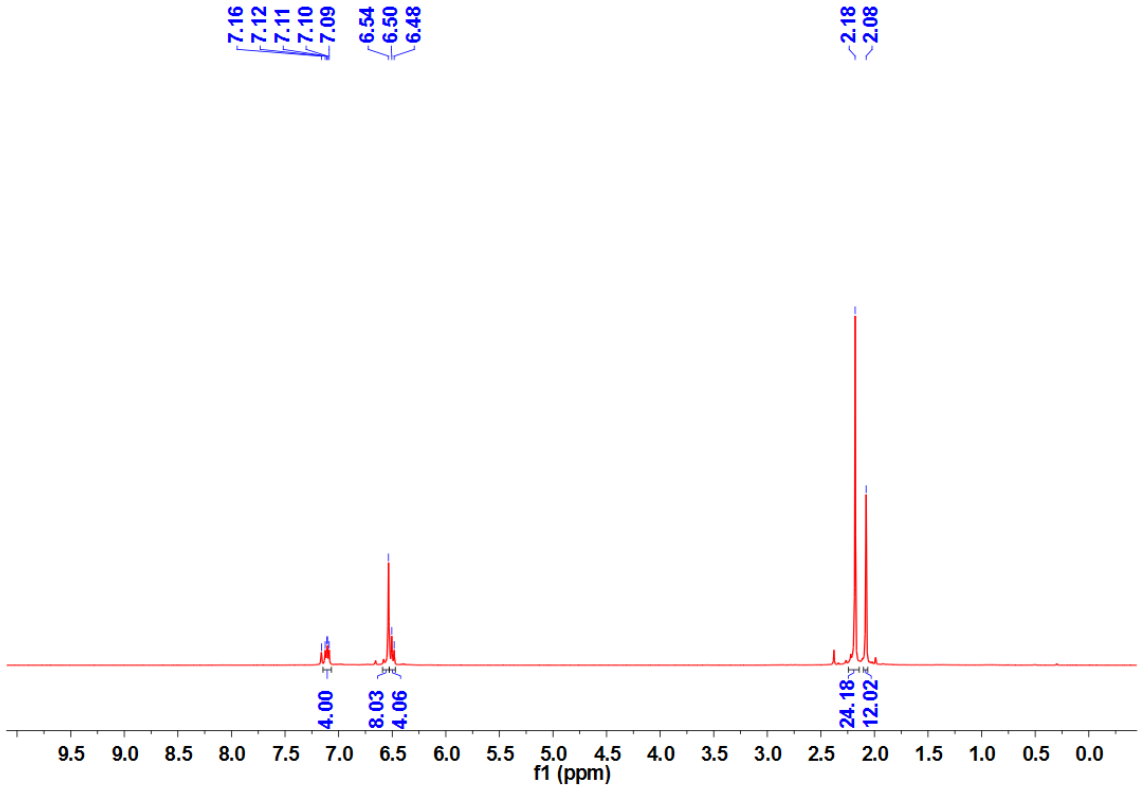

Figure S31. ^{1}H NMR spectrum of **4b** in C_6D_6.

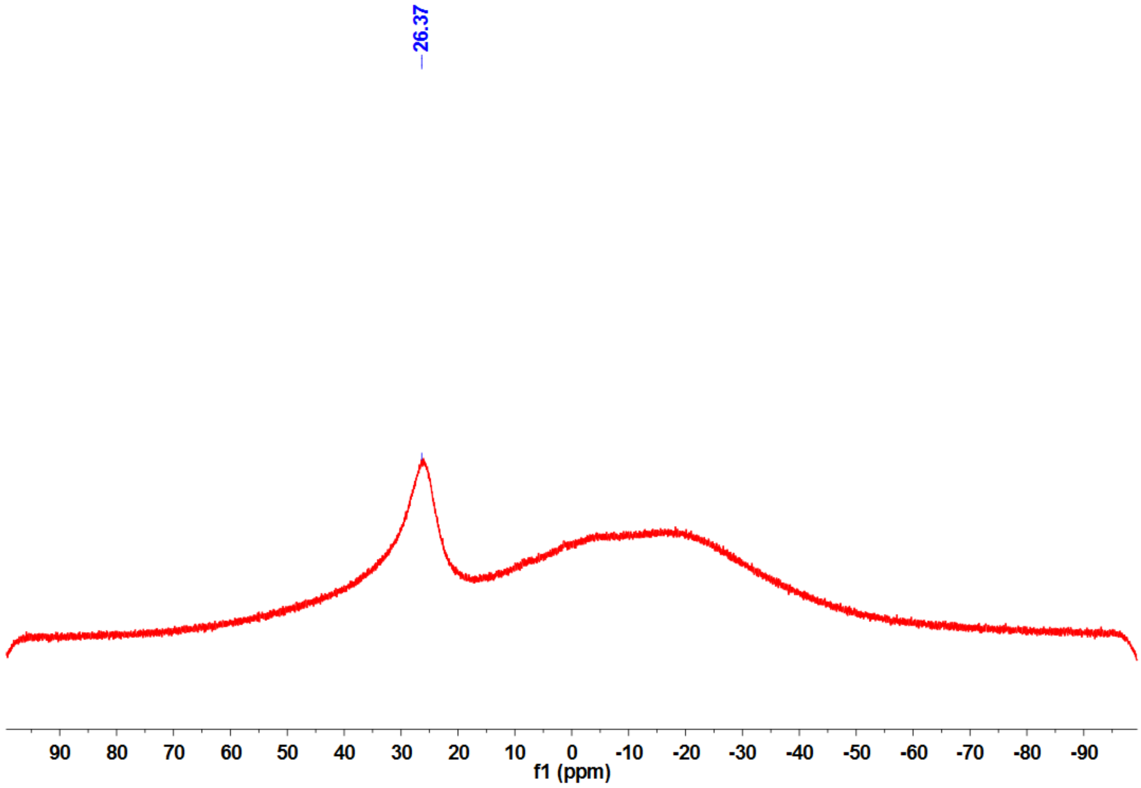

Figure S32. ^{11}B NMR spectrum of **4b** in C_6D_6.

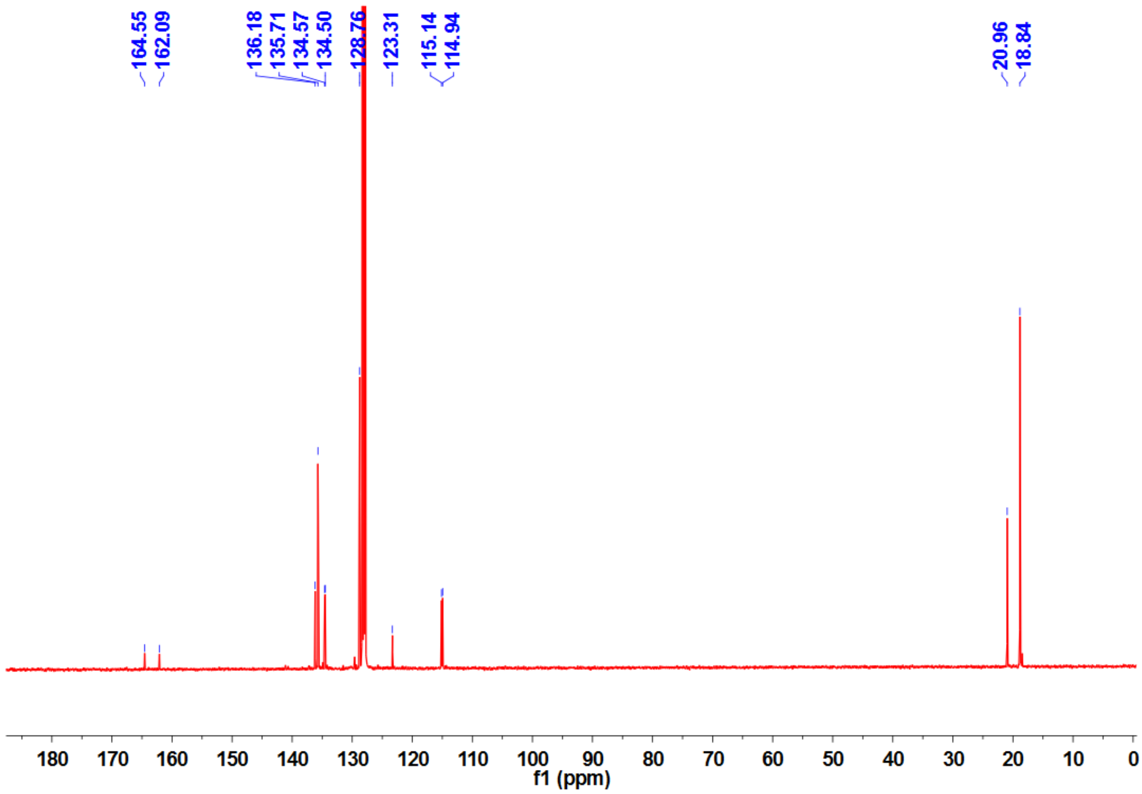

Figure S33. $^{13}C\{^{1}H\}$ NMR spectrum of **4b** in C_6D_6.

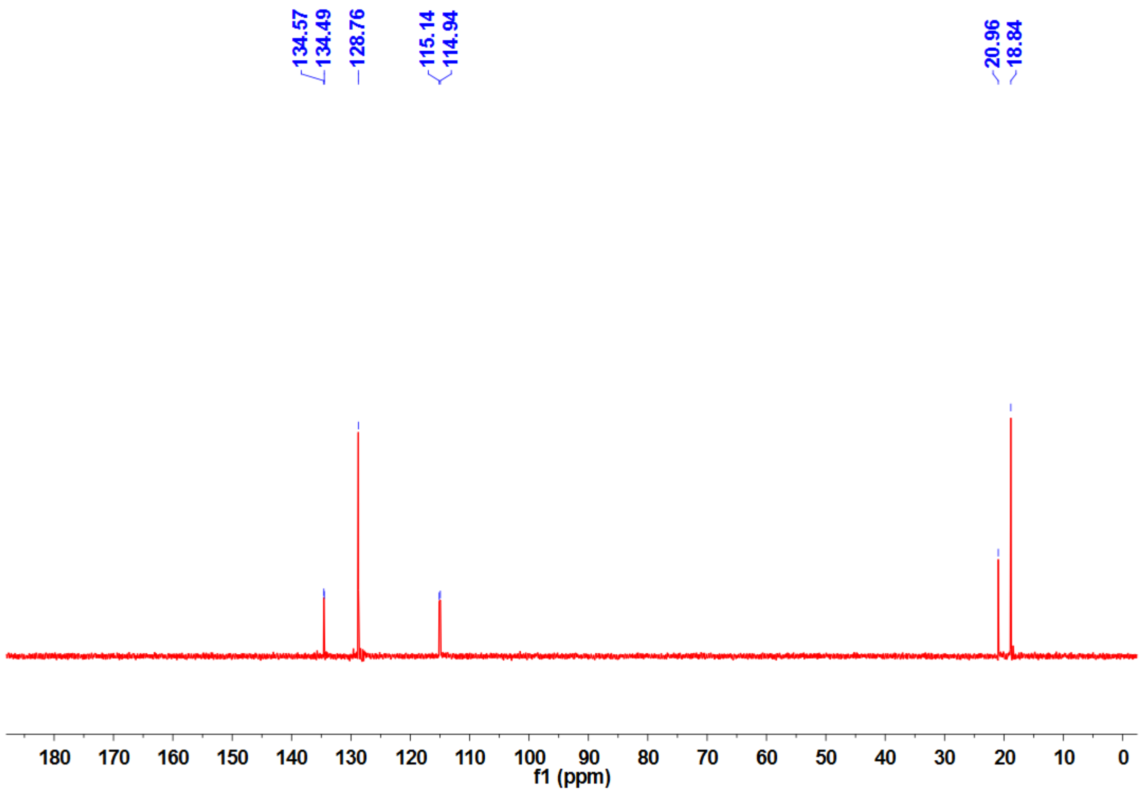

Figure S34. ^{13}C (DEPT135) NMR spectrum of **4b** in C_6D_6.

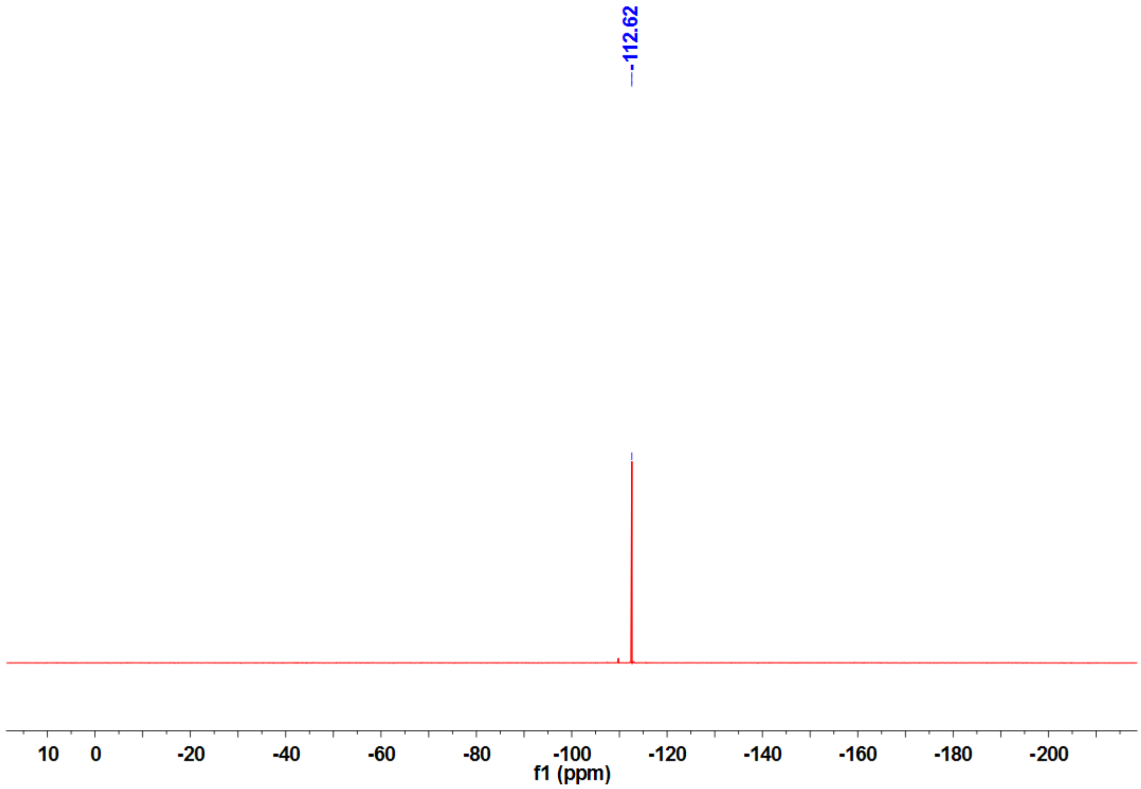

Figure S35. $^{19}F\{^{1}H\}$ NMR spectrum of **4b** in C_6D_6.

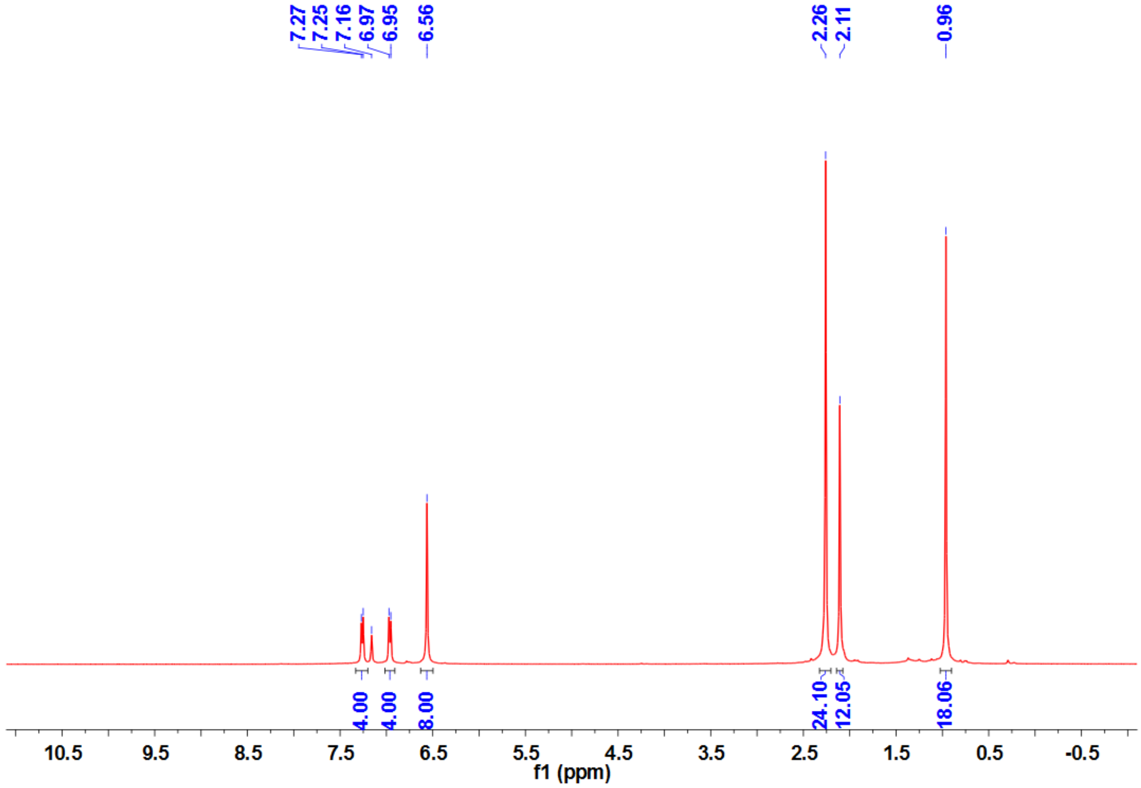

Figure S36. ^{1}H NMR spectrum of **4c** in C_6D_6.

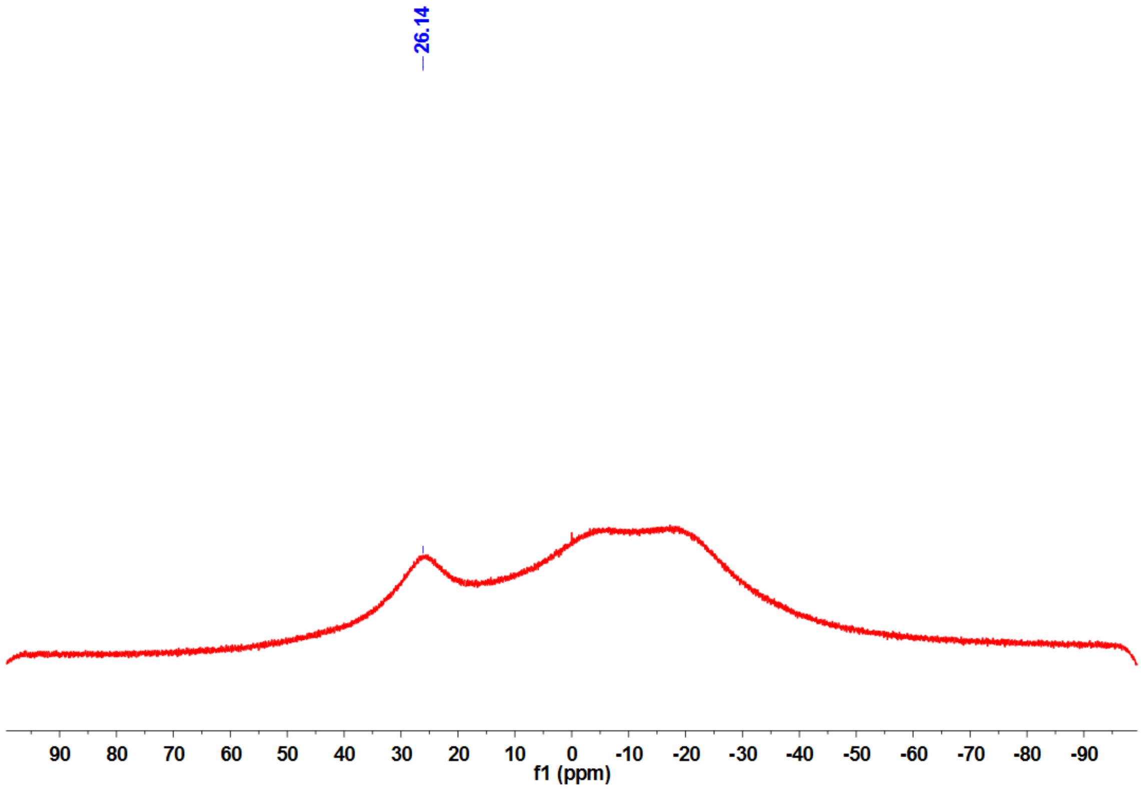

Figure S37. ^{11}B NMR spectrum of **4c** in C_6D_6.

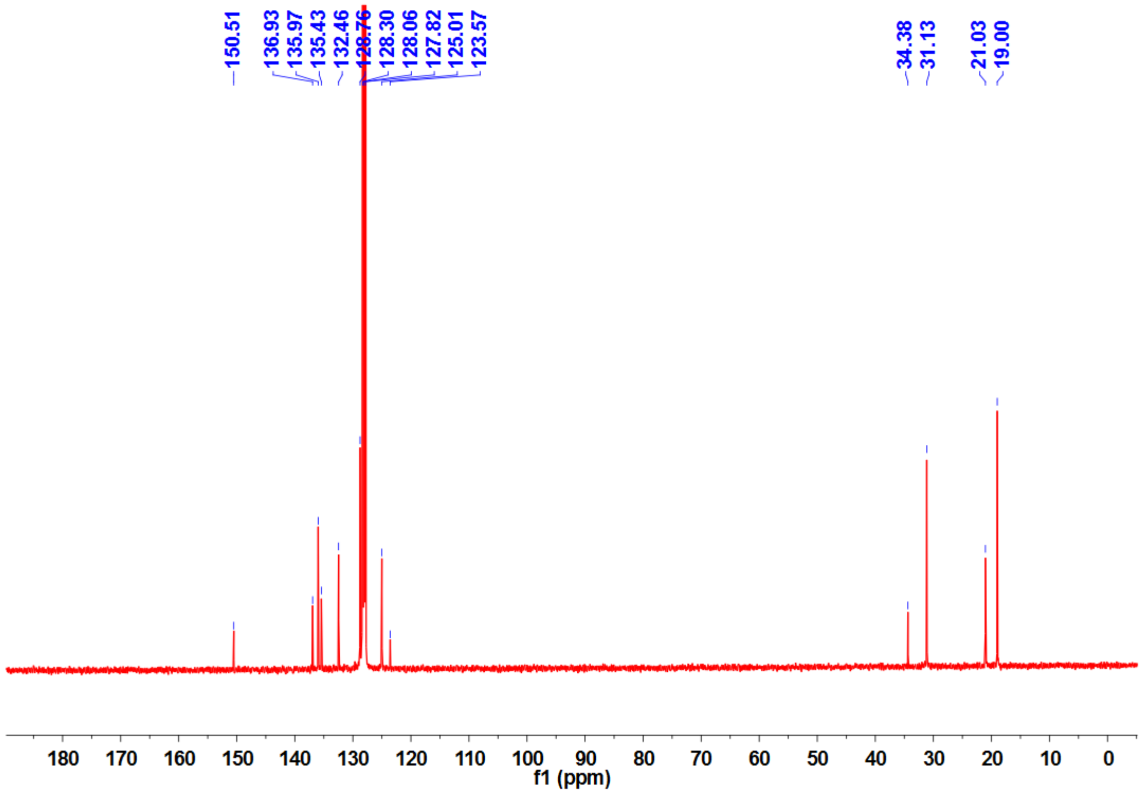

Figure S38. $^{13}C\{^1H\}$ NMR spectrum of **4c** in C_6D_6.

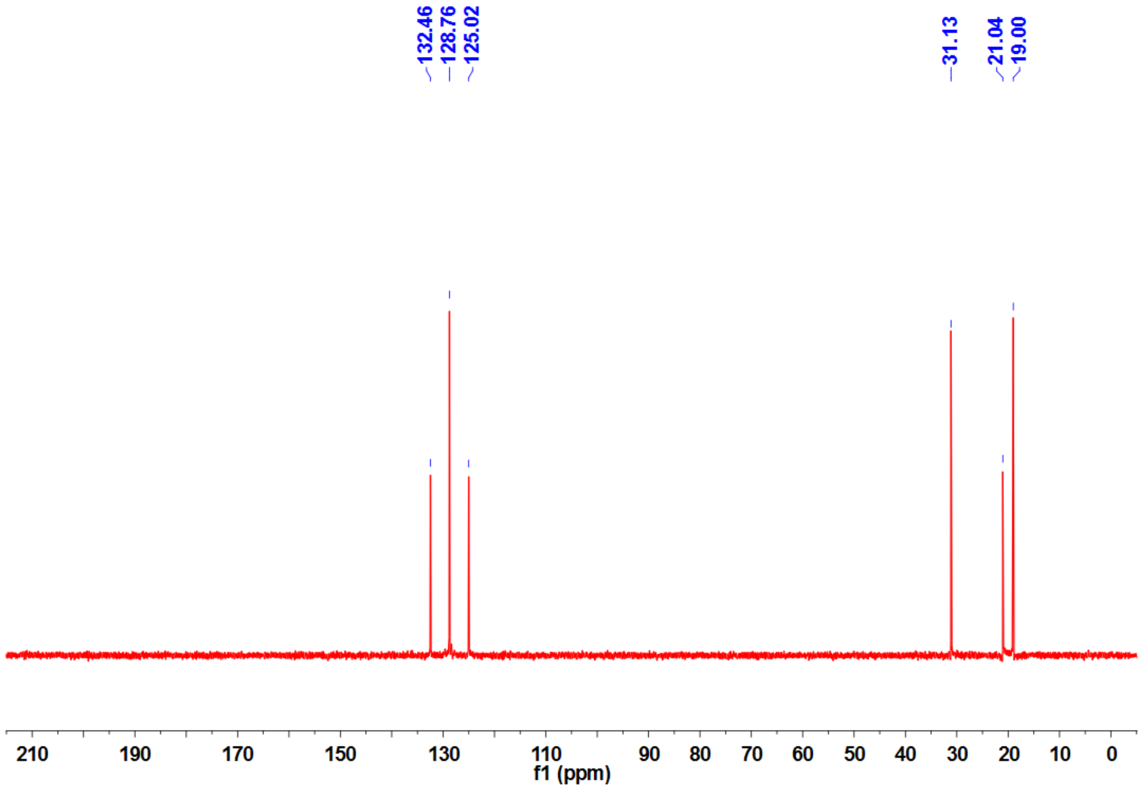

Figure S39. ^{13}C (DEPT135) NMR spectrum of **4c** in C_6D_6.

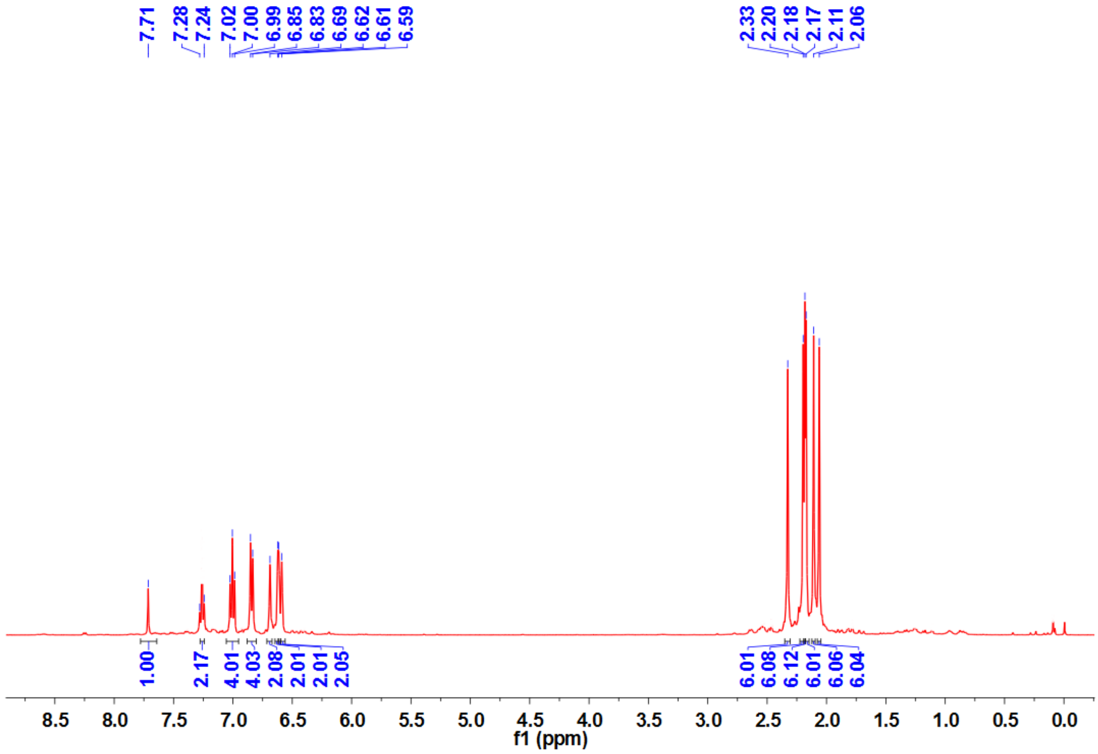

Figure S40. ^{1}H NMR spectrum of **5a** in $CDCl_3$.

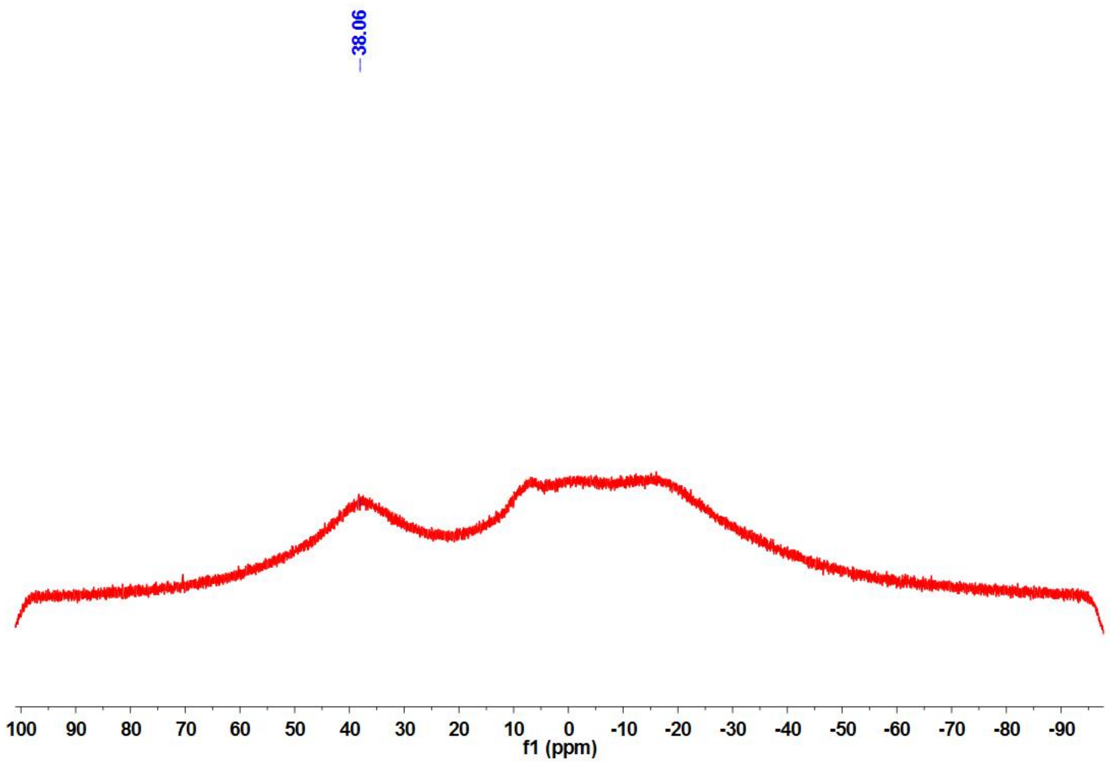

Figure S41. ^{11}B NMR spectrum of **5a** in $CDCl_3$.

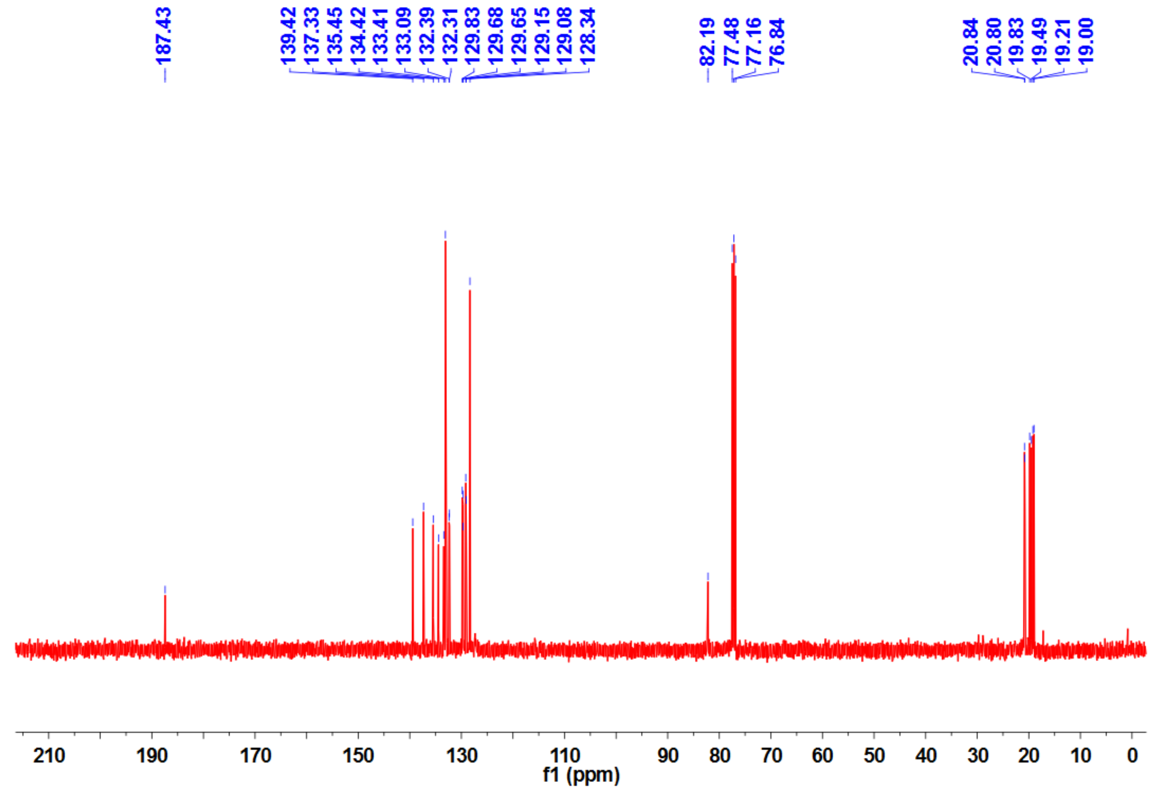

Figure S42. $^{13}C\{^1H\}$ NMR spectrum of **5a** in $CDCl_3$.

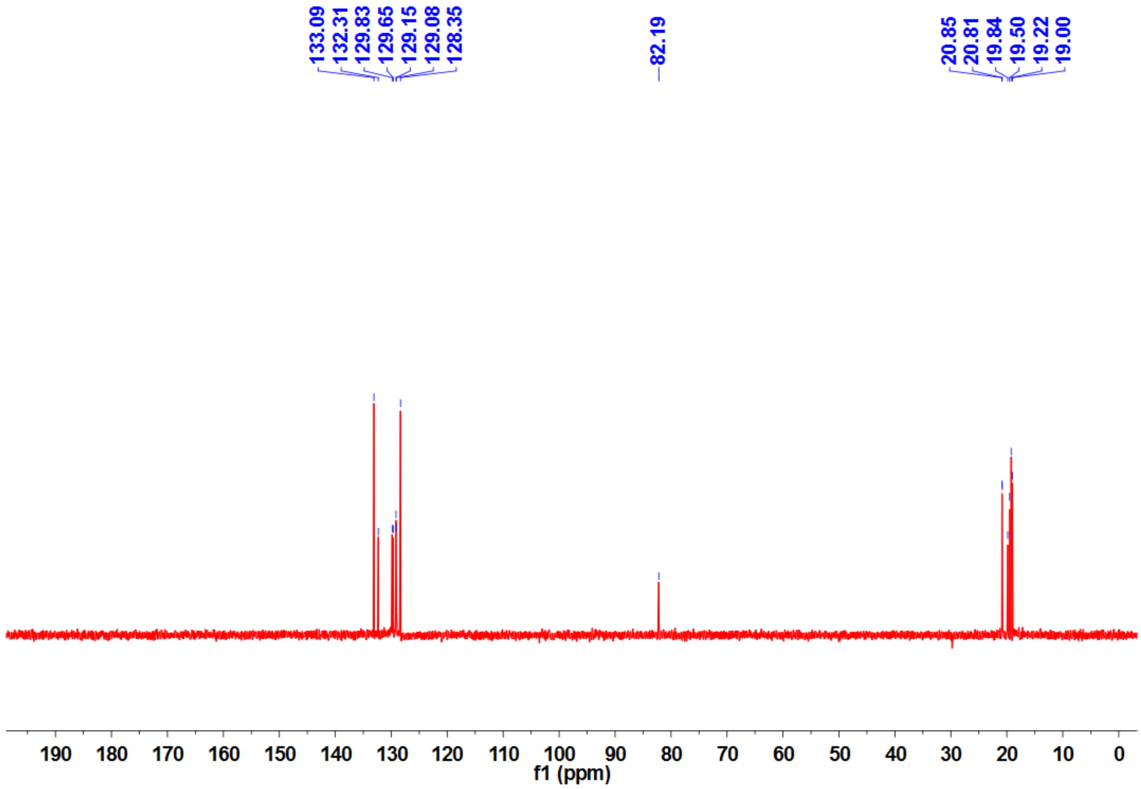

Figure S43. ^{13}C (DEPT135) NMR spectrum of **5a** in $CDCl_3$.

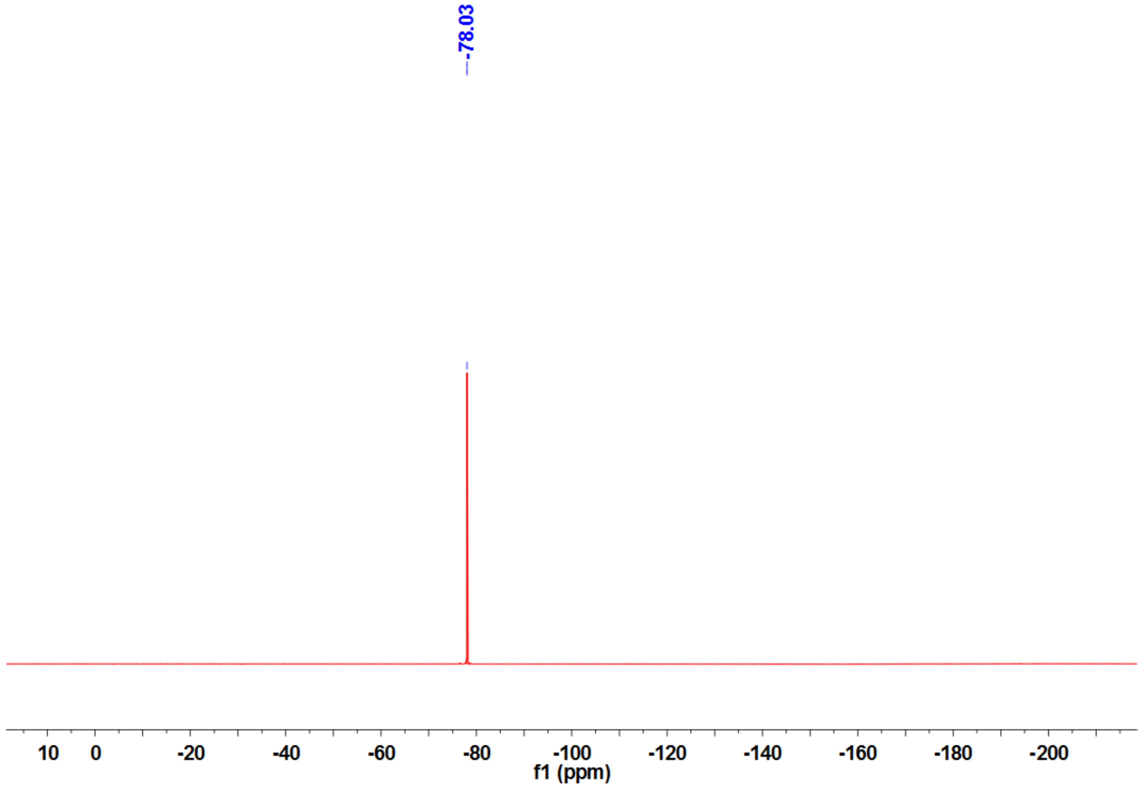

Figure S44. $^{19}F\{^{1}H\}$ NMR spectrum of **5a** in $CDCl_3$.

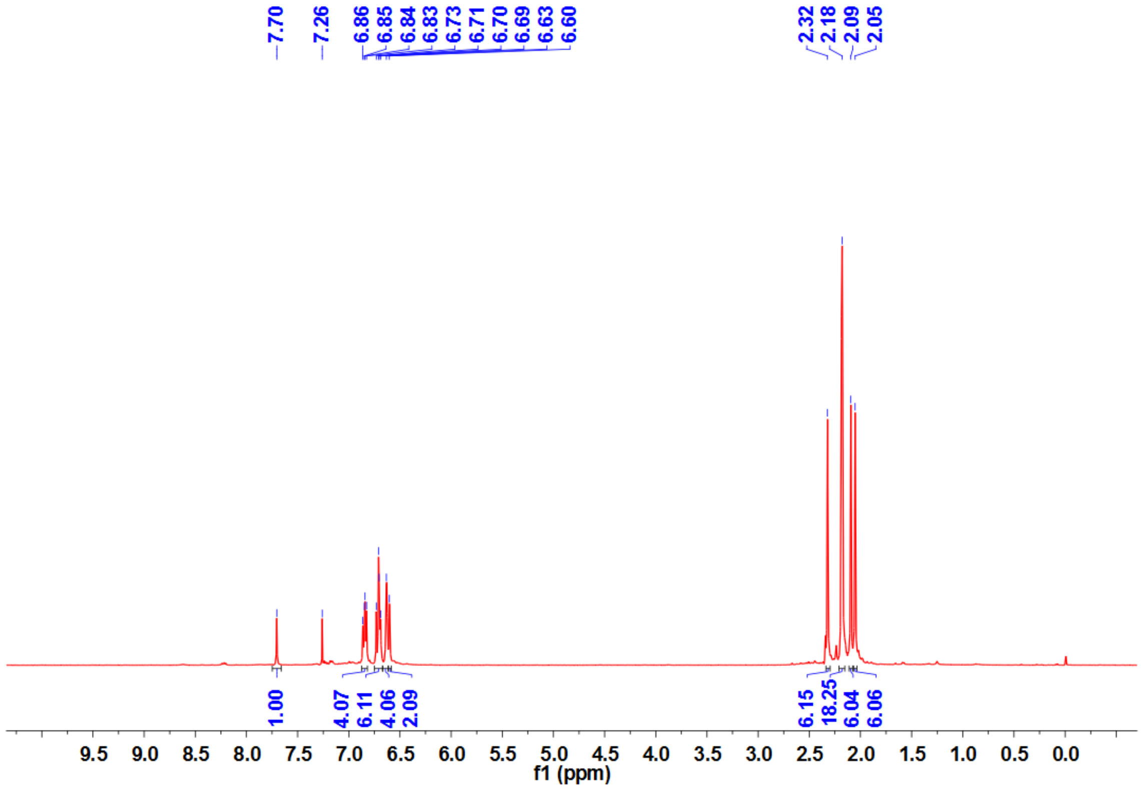

Figure S45. ^{1}H NMR spectrum of **5b** in $CDCl_3$.

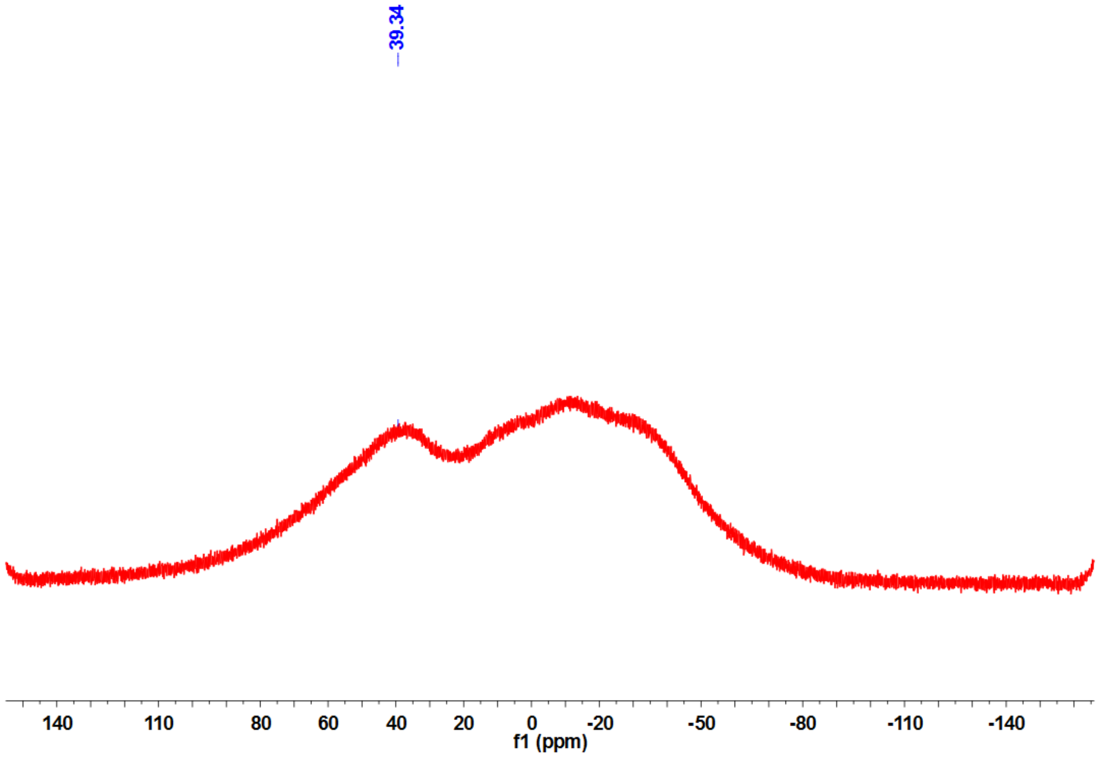

Figure S46. ^{11}B NMR spectrum of **5b** in $CDCl_3$.

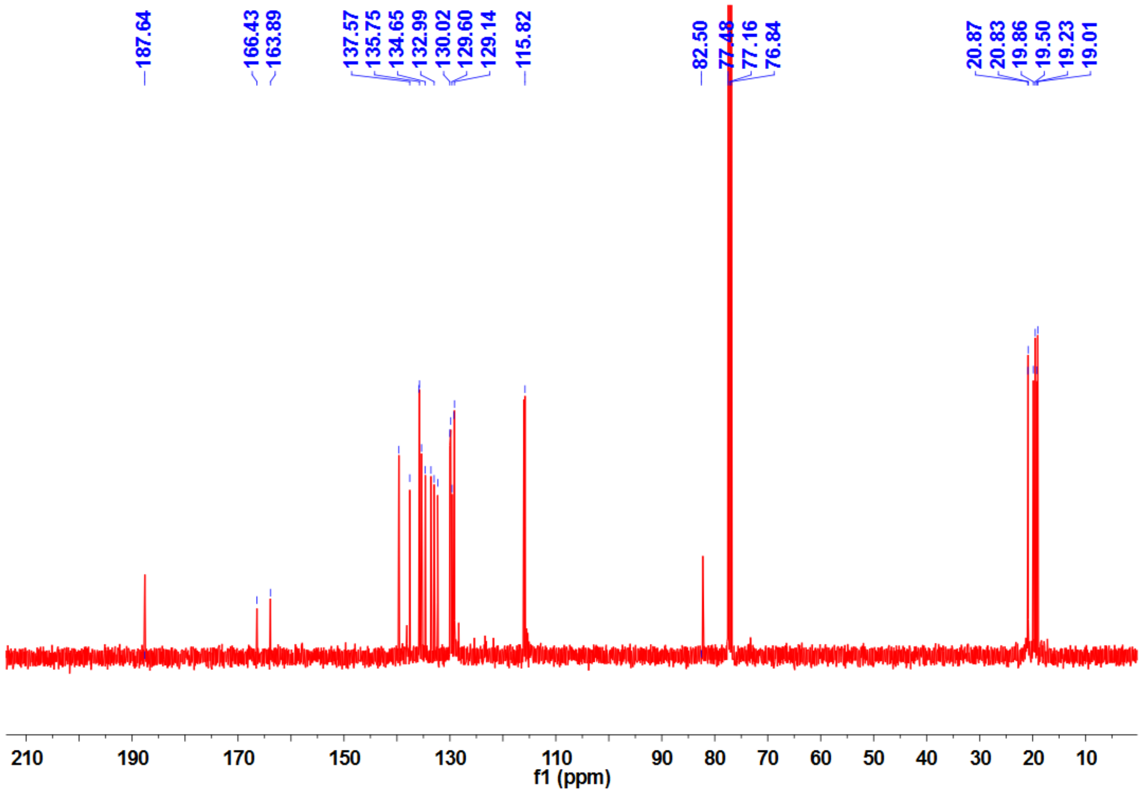

Figure S47. $^{13}C\{^{1}H\}$ NMR spectrum of **5b** in $CDCl_3$.

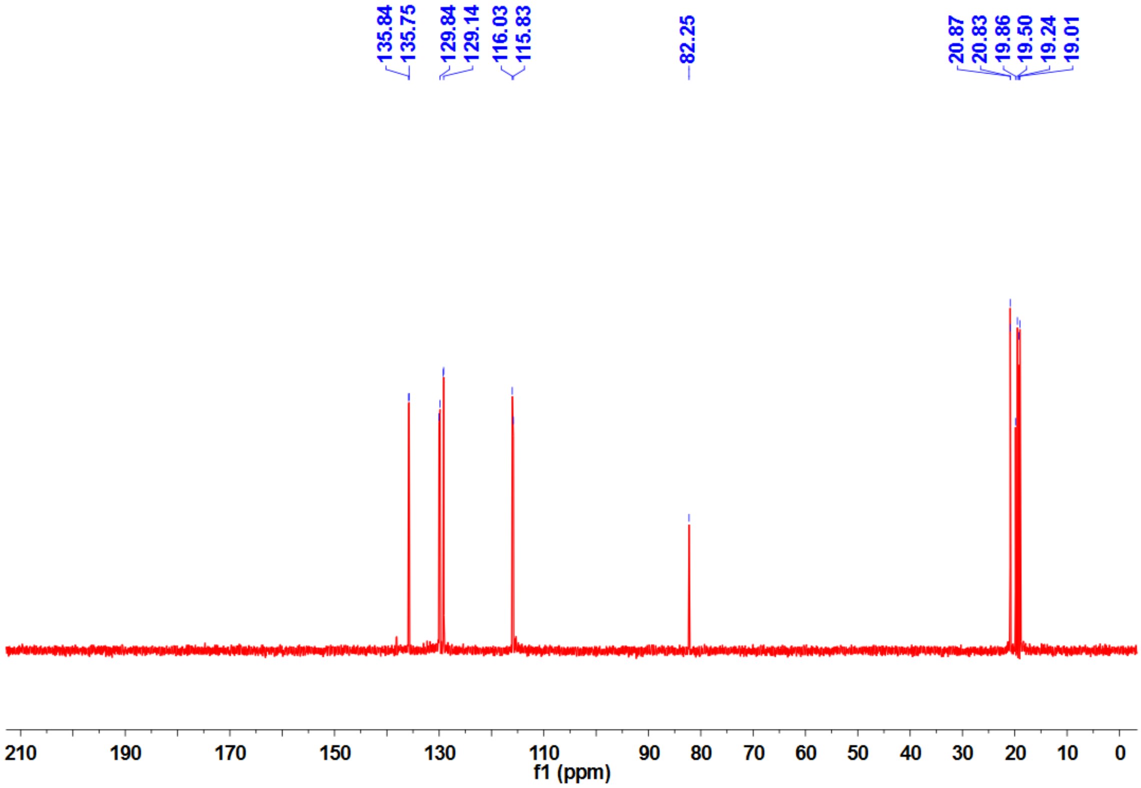

Figure S48. ^{13}C (DEPT135) NMR spectrum of **5b** in $CDCl_3$.

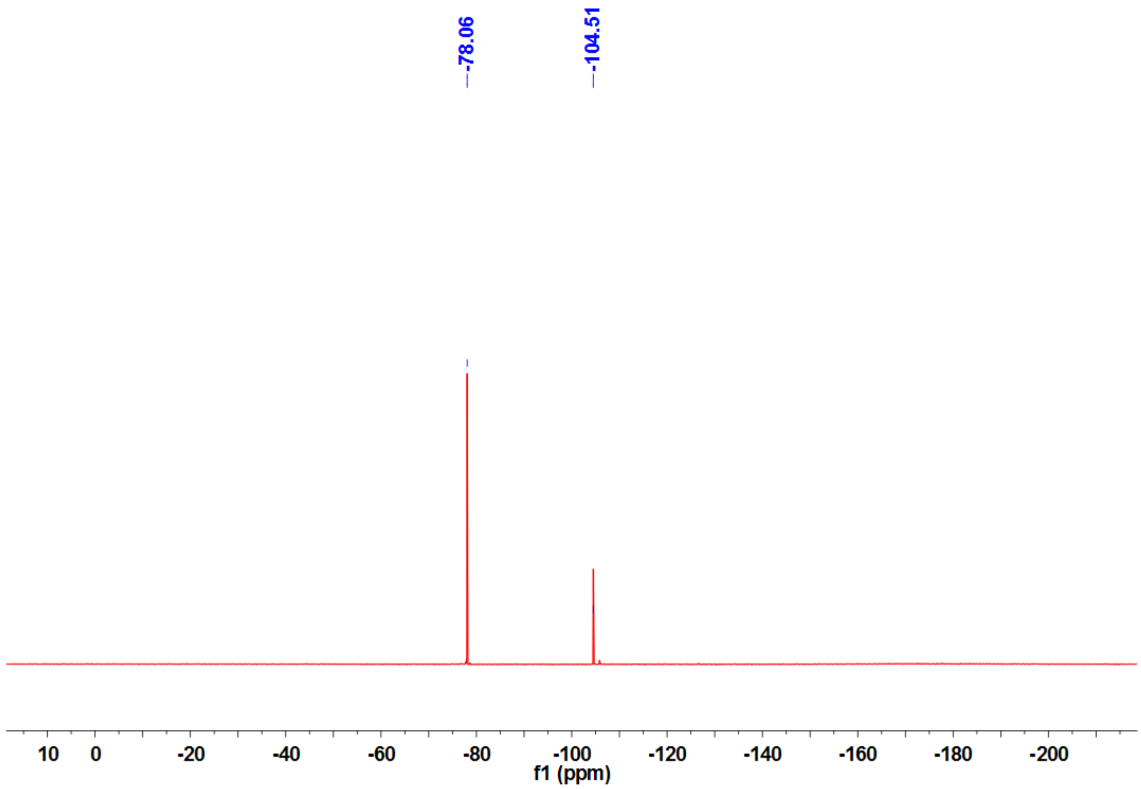

Figure S49. $^{19}F\{^{1}H\}$ NMR spectrum of **5b** in $CDCl_3$.

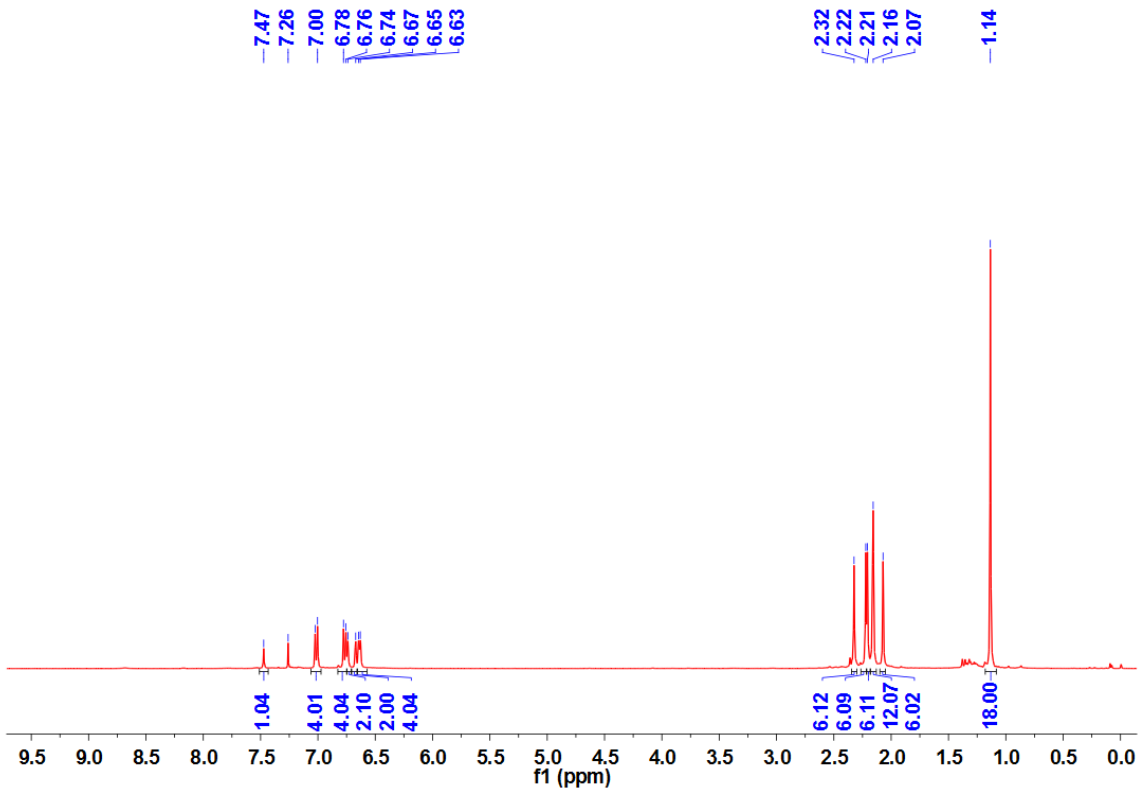

Figure S50. ^{1}H NMR spectrum of **5c** in $CDCl_3$.

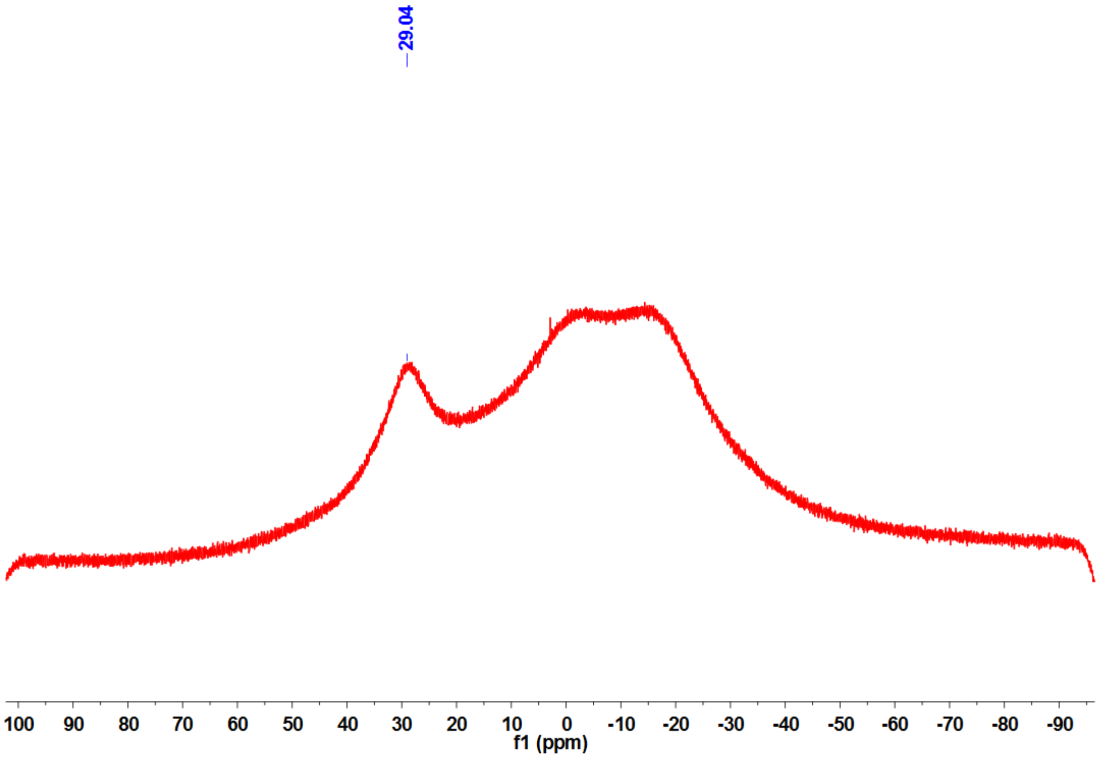

Figure S51. ^{11}B NMR spectrum of **5c** in $CDCl_3$.

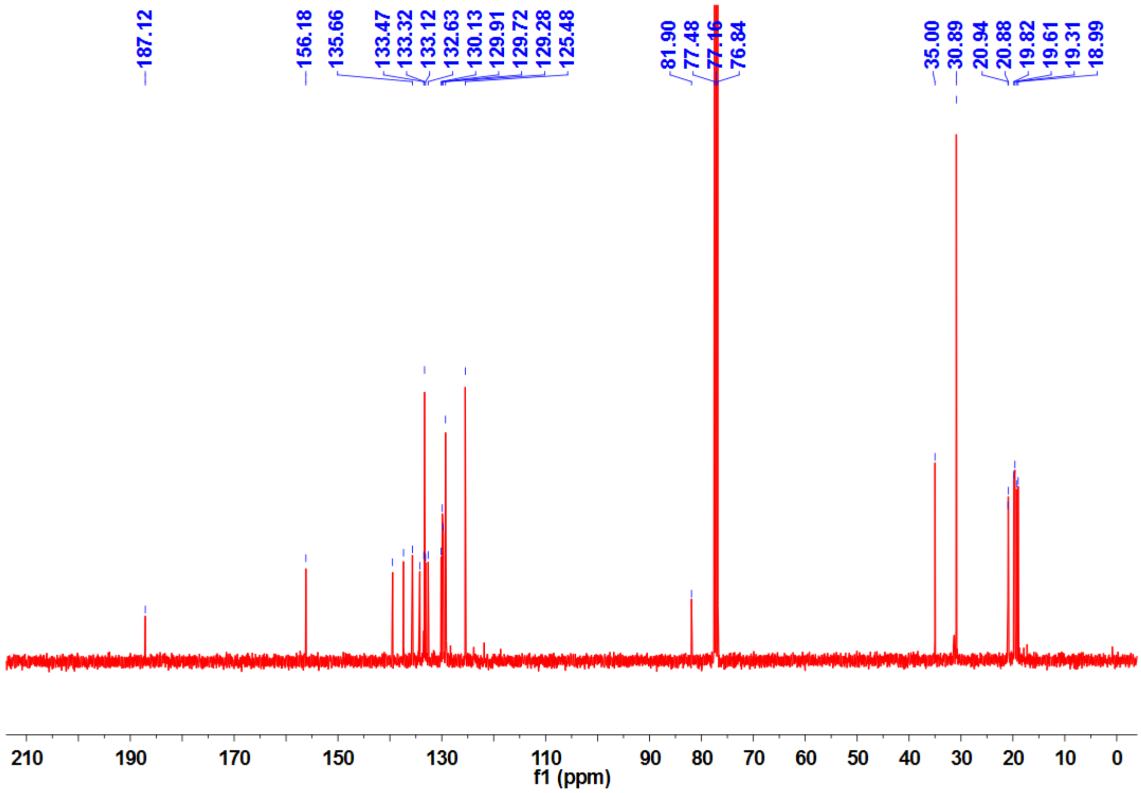

Figure S52. ^{13}C{^{1}H} NMR spectrum of **5c** in $CDCl_3$.

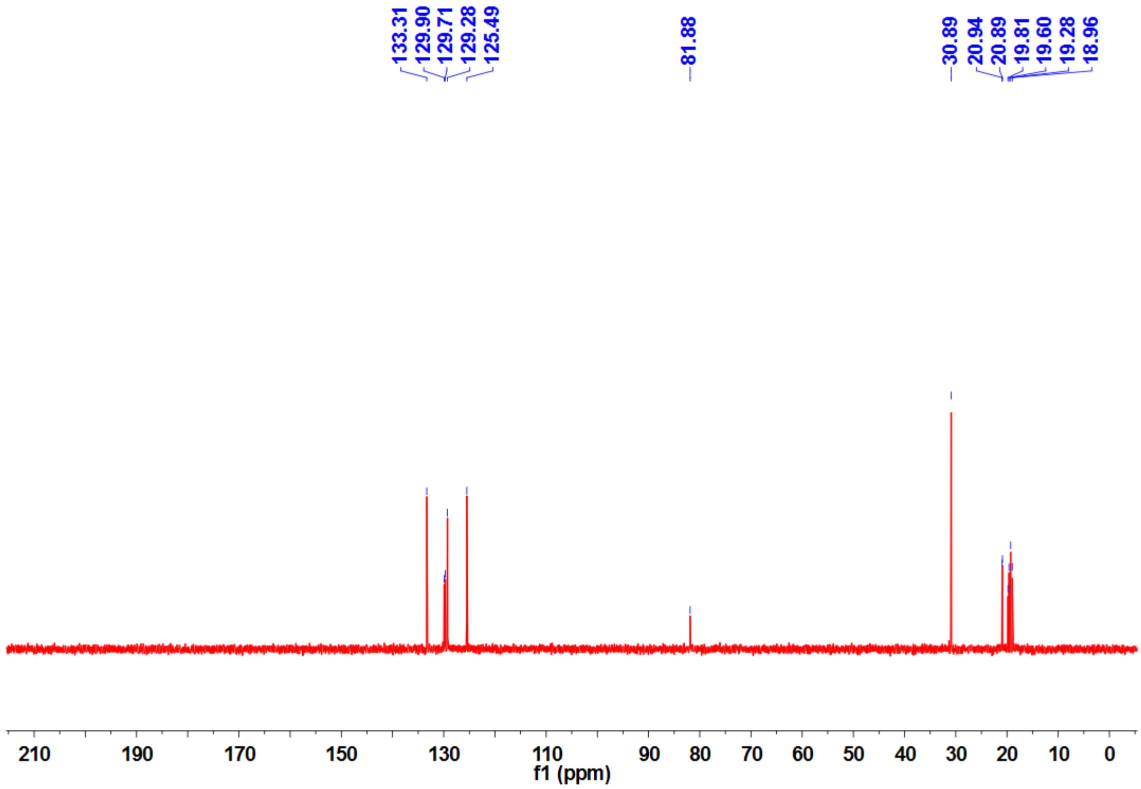

Figure S53. ^{13}C (DEPT135) NMR spectrum of **5c** in $CDCl_3$.

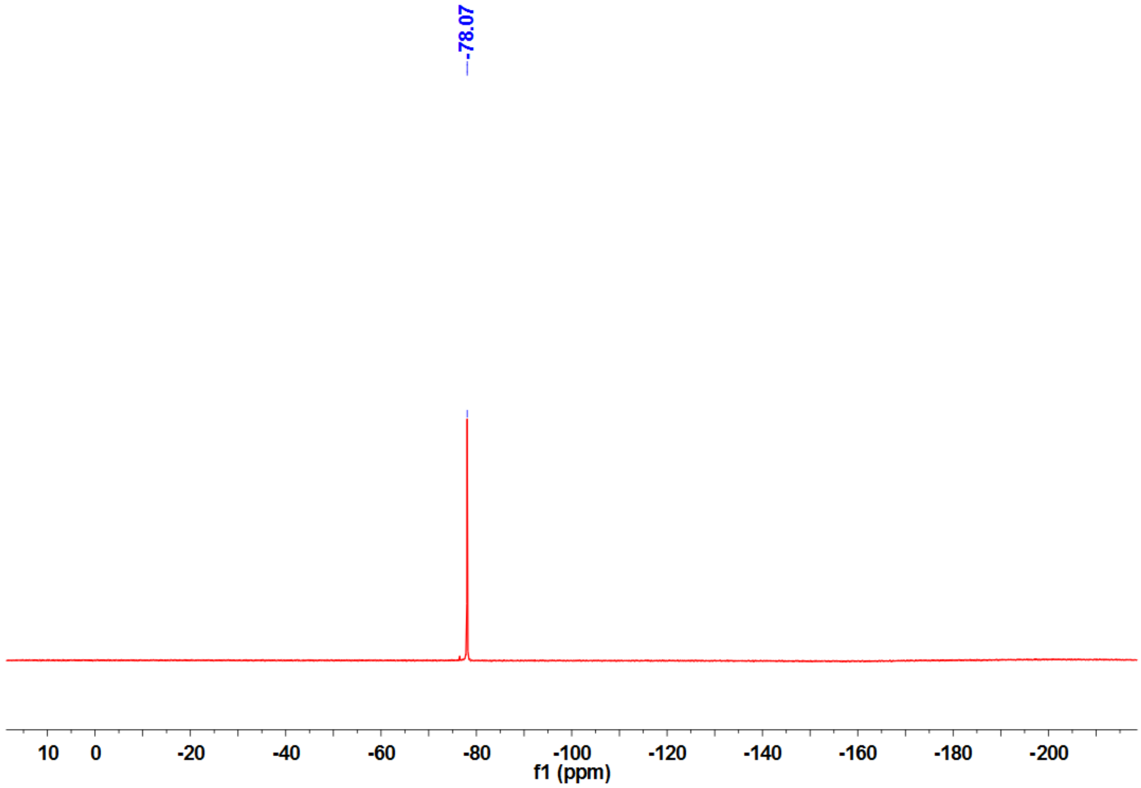

Figure S54. ^{19}F{^{1}H} NMR spectrum of **5c** in $CDCl_3$.

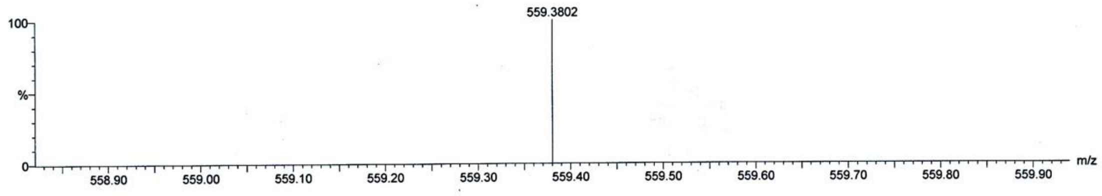

Minimum:				-1.5			
Maximum:		10.0	10.0	50.0			
Mass	Calc. Mass	mDa	PPM	DBE	i-FIT	i-FIT (Norm)	Formula
559.3802	559.3801	0.1	0.2	17.5	13.3	0.0	C38 H47 N4

Figure S55. HRMS spectrum of **1**.

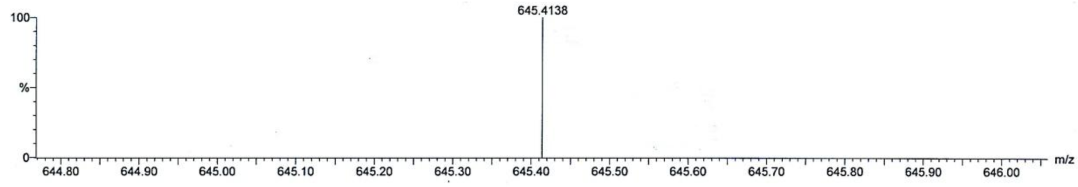

Minimum:				-1.5			
Maximum:		10.0	10.0	50.0			
Mass	Calc. Mass	mDa	PPM	DBE	i-FIT	i-FIT (Norm)	Formula
645.4138	645.4129	0.9	1.4	22.5	16.7	0.0	C44 H50 B N4

Figure S56. HRMS spectrum of **2a**.

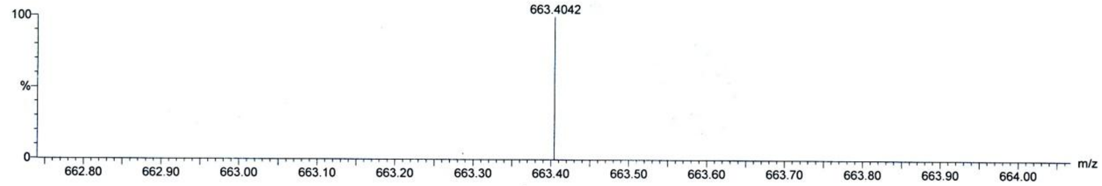

Minimum:				-1.5			
Maximum:		10.0	10.0	50.0			
Mass	Calc. Mass	mDa	PPM	DBE	i-FIT	i-FIT (Norm)	Formula
663.4042	663.4034	0.8	1.2	22.5	65.8	0.0	C44 H49 N4 B F

Figure S57. HRMS spectrum of **2b**.

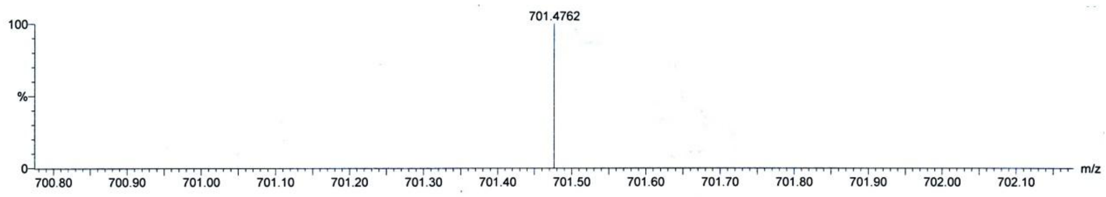

Minimum:				-1.5			
Maximum:		10.0	10.0	50.0			
Mass	Calc. Mass	mDa	PPM	DBE	i-FIT	i-FIT (Norm)	Formula
701.4762	701.4755	0.7	1.0	22.5	13.2	0.0	C48 H58 B N4

Figure S58. HRMS spectrum of **2c**.

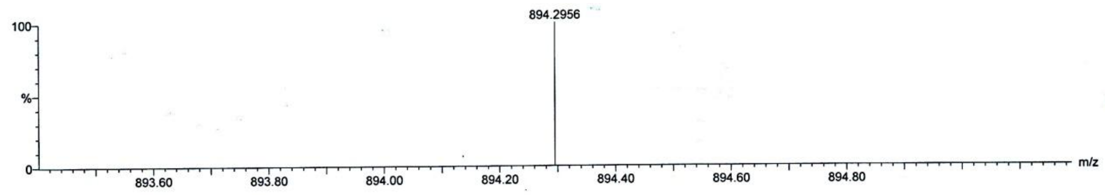

Minimum:				-1.5			
Maximum:		10.0	10.0	50.0			
Mass	Calc. Mass	mDa	PPM	DBE	i-FIT	i-FIT (Norm)	Formula
894.2956	894.2975	-1.9	-2.1	25.5	13.3	0.0	C50 H55 N4 81Br2 10B 11B

Figure S59. HRMS spectrum of **3a**.

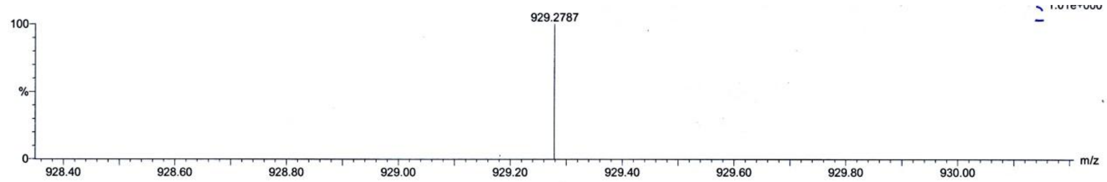

Minimum:				-1.5			
Maximum:		5.0	10.0	50.0			
Mass	Calc. Mass	mDa	PPM	DBE	i-FIT	i-FIT (Norm)	Formula
929.2787	929.2771	1.6	1.7	25.5	15.1	0.0	C50 H53 B2 N4 79Br 81Br F2

Figure S60. HRMS spectrum of **3b**.

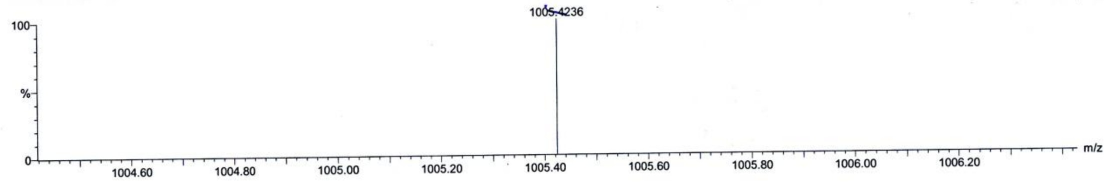

Minimum:				-1.5			
Maximum:		5.0	10.0	50.0			
Mass	Calc. Mass	mDa	PPM	DBE	i-FIT	i-FIT (Norm)	Formula
1005.4236	1005.4211	2.5	2.5	25.5	15.1	0.0	C58 H71 B2 N4 79Br 81Br

Figure S61. HRMS spectrum of **3c**.

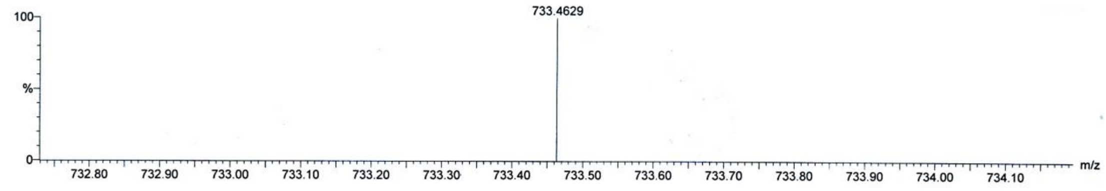

Minimum:				-1.5			
Maximum:		10.0	10.0	50.0			
Mass	Calc. Mass	mDa	PPM	DBE	i-FIT	i-FIT (Norm)	Formula
733.4629	733.4613	1.6	2.2	26.5	8.1	0.0	C50 H55 B2 N4

Figure S62. HRMS spectrum of **4a**.

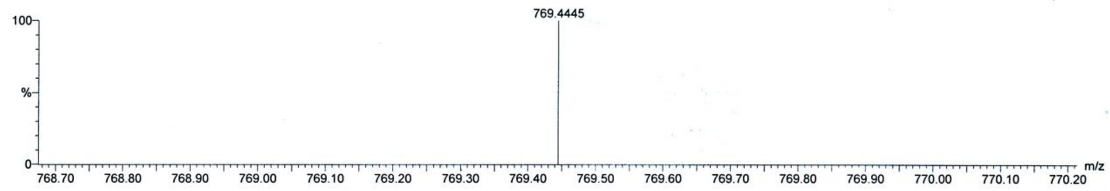

Minimum:				-1.5			
Maximum:		10.0	10.0	50.0			
Mass	Calc. Mass	mDa	PPM	DBE	i-FIT	i-FIT (Norm)	Formula
769.4445	769.4424	2.1	2.7	26.5	10.4	0.0	C50 H53 B2 N4 F2

Figure S63. HRMS spectrum of **4b**.

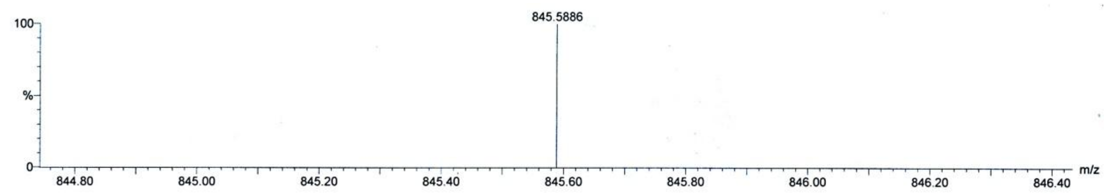

Minimum:				-1.5			
Maximum:		10.0	10.0	50.0			
Mass	Calc. Mass	mDa	PPM	DBE	i-FIT	i-FIT (Norm)	Formula
845.5886	845.5865	2.1	2.5	26.5	10.5	0.0	C58 H71 B2 N4

Figure S64. HRMS spectrum of **4c**.

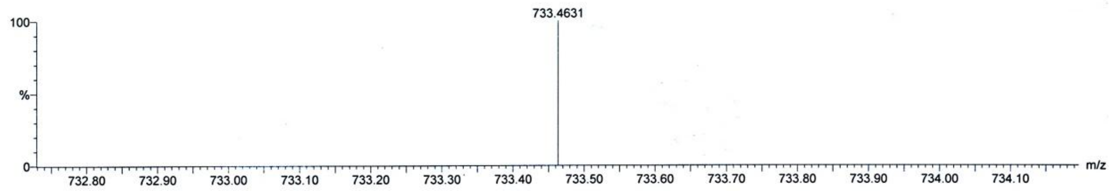

Minimum:				-1.5			
Maximum:		10.0	10.0	50.0			
Mass	Calc. Mass	mDa	PPM	DBE	i-FIT	i-FIT (Norm)	Formula
733.4631	733.4613	1.8	2.5	26.5	10.3	0.0	C50 H55 B2 N4

Figure S65. HRMS spectrum of **4a**$^{\bullet+}$.

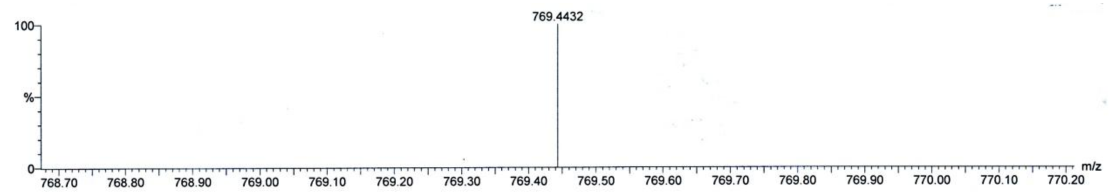

Minimum:				-1.5			
Maximum:		10.0	10.0	50.0			
Mass	Calc. Mass	mDa	PPM	DBE	i-FIT	i-FIT (Norm)	Formula
769.4432	769.4424	0.8	1.0	26.5	8.9	0.0	C50 H53 N4 B2 F2

Figure S66. HRMS spectrum of **4b**$^{\bullet+}$.

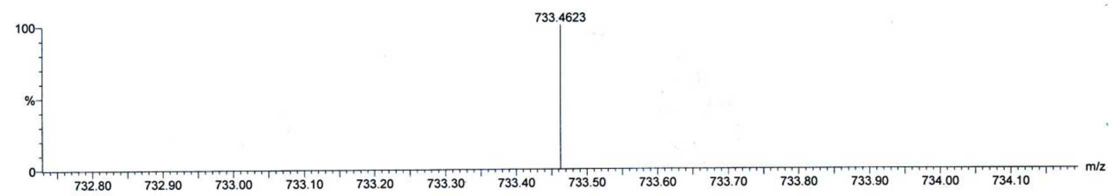

Minimum:				-1.5			
Maximum:		10.0	10.0	50.0			
Mass	Calc. Mass	mDa	PPM	DBE	i-FIT	i-FIT (Norm)	Formula
733.4623	733.4613	1.0	1.4	26.5	10.3	0.0	C50 H55 B2 N4

Figure S67. HRMS spectrum of **5a**.

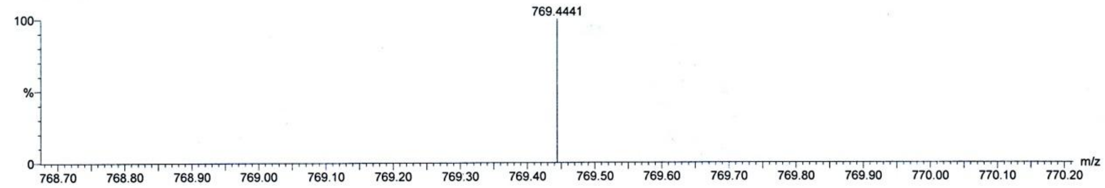

Minimum:				-1.5			
Maximum:		10.0	10.0	50.0			
Mass	Calc. Mass	mDa	PPM	DBE	i-FIT	i-FIT (Norm)	Formula
769.4441	769.4424	1.7	2.2	26.5	8.1	0.0	C50 H53 B2 N4 F2

Figure S68. HRMS spectrum of **5b**.

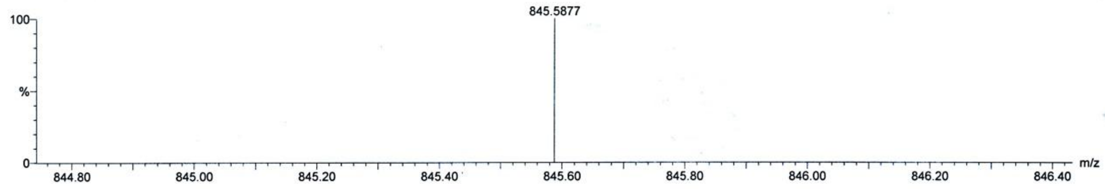

Minimum:				-1.5			
Maximum:		10.0	10.0	50.0			
Mass	Calc. Mass	mDa	PPM	DBE	i-FIT	i-FIT (Norm)	Formula
845.5877	845.5865	1.2	1.4	26.5	10.5	0.0	C58 H71 B2 N4

Figure S69. HRMS spectrum of **5c**.

2. Crystal structural parameters and solid structures

Intensity data for compounds **2a, 3a, 4a, 4b, [4a$^{•+}$]•[B(C_6F_5)$_4$], [4b$^{•+}$]•[B(C_6F_5)$_4$],** and **5a** were collected using a Bruker APEX II diffractometer. The structure was solved by direct phase determination (SHELX-2013) and refined for all data by full-matrix least squares methods on *F2*.[S5] All non-hydrogen atoms were subjected to anisotropic refinement. The hydrogen atoms were generated geometrically and allowed to ride in their respective parent atoms; they were assigned appropriate isotropic thermal parameters and included in the structure factor calculations. CCDC: 1548460-1548466 contains the supplementary crystallographic data for this paper. The data can be obtained free of charge from the Cambridge Crystallography Data Center via www.ccdc.cam.ac.uk/data_request/cif.

Table S1. Summary of Data Collection and Structure Refinement.

Compounds	**2a**	**3a•($CHCl_3$)$_2$**	**4a**	**[4a$^{•+}$]•[B(C_6F_5)$_4$]$^-$•toluene**
Formula	$C_{44}H_{49}BN_4$	$C_{52}H_{56}B_2Br_2Cl_6N_4$	$C_{50}H_{54}B_2N_4$	$C_{81}H_{62}B_3F_{20}N_4$
Fw	644.68	1131.14	732.59	1503.77
Crystal syst	Monoclinic	Triclinic	Monoclinic	Monoclinic
Space group	C 1 c 1	P-1	C 1 2/c 1	P 1 21/n 1
Size, mm^3	0.240 x 0.390 x 0.400	0.100 x 0.280 x 0.400	0.100 x 0.120 x 0.220	0.200 x 0.300 x 0.360
T, K	103(2)	103(2)	103(2)	143(2)
a, Å	21.931(3)	9.2160(12)	15.662(3)	17.0654(2)
b, Å	12.2382(17)	10.4837(15)	17.724(3)	18.7504(2)
c, Å	14.940(2)	14.3635(19)	16.037(3)	22.9274(3)
α, deg	90	81.176(4)	90	90
β, deg	108.564(4)	78.995(4)	109.914(5)	97.0523(5)
γ, deg	90	73.107(4)	90	90
V, Å^3	3801.2(9)	1296.4(3)	4185.6(12)	7280.88(15)
Z	4	1	4	4
d_{calcd}, *g*•cm^{-1}	1.127	1.449	1.163	1.372
μ, mm^{-1}	0.065	1.912	0.067	0.994
Refl collected	30037	27435	17039	74879
T_{min}/T_{max}	0.9740/0.9840	0.5150/0.8320	0.9850/0.9930	0.8260/0.7160
N_{measd}	11929	6956	3677	12735
[R_{int}]	0.0571	0.0963	0.1729	0.0555
R[I>2sigma(I)]	0.0601	0.0500	0.0782	0.0532
R*w*[I>2sigma(I)]	0.1148	0.0908	0.1357	0.1588
GOF	1.009	0.999	1.006	1.085
Largest diff. Peak/hole, e•Å^{-3}	0.277/-0.302	0.913/-0.722	0.323/-0.268	0.439/-0.423

Compounds	**4b**	**$[4b^{\bullet+}]\bullet[B(C_6F_5)_4]^-\bullet$toluene**	**$5a\bullet(CH_2Cl_2)_2$**
Formula	$C_{53}H_{59}B_2N_4Cl_4F_3SO_3$	$C_{81}H_{62}B_3F_{20}N_4$	$C_{53}H_{59}B_2N_4Cl_4F_3SO_3$
Fw	1052.52	1503.77	1052.52
Crystal syst	Monoclinic	Monoclinic	Monoclinic
Space group	C 1 c 1	P 1 21/n 1	C 1 c 1
Size, mm^3	0.390 x 0.400 x 0.420	0.200 x 0.300 x 0.360	0.390 x 0.400 x 0.420
T, K	153(2)	143(2)	153(2)
a, Å	21.6523(7)	17.0654(2)	21.6523(7)
b, Å	12.0310(4)	18.7504(2)	12.0310(4)
c, Å	22.3972(7)	22.9274(3)	22.3972(7)
α, deg	90	90	90
β, deg	117.8819(13)	97.0523(5)	117.8819(13)
γ, deg	90	90	90
V, $Å^3$	5157.1(3)	7280.88(15)	5157.1(3)
Z	4	4	4
d_{calcd}, $g\bullet cm^{-1}$	1.356	1.372	1.356
μ, mm^{-1}	0.328	0.994	0.328
Refl collected	62211	74879	62211
T_{min}/T_{max}	0.8830/0.8740	0.8260/0.7160	0.8830/0.8740
N_{measd}	14845	12735	14845
$[R_{int}]$	0.0627	0.0555	0.0627
R[I>2sigma(I)]	0.0628	0.0532	0.0628
R*w*[I>2sigma(I)]	0.1553	0.1588	0.1553
GOF	1.019	1.085	1.019
Largest diff. Peak/hole, $e\bullet Å^{-3}$	0.813/-0.631	0.439/-0.423	0.813/-0.631

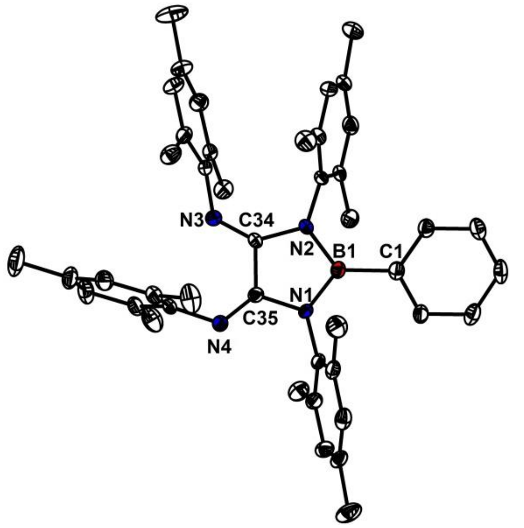

Figure S70. Solid structures of **2a** (hydrogen atoms are omitted for clarity). Thermal ellipsoids are set at the 50% probability. Selected bond lengths (Å) and angles (°): B1−N1 1.433(4), B1−N2 1.449(4), B1−C1 1.558(4), N1−C35 1.398(3), N2−C34 1.411(3), N3−C34 1.267(3), N4−C35 1.270(3), C34−C35 1.516(4), N1−B1−N2 107.2(2), N1−B1−C1 127.3(3), N2−B1−C1 125.4(2).

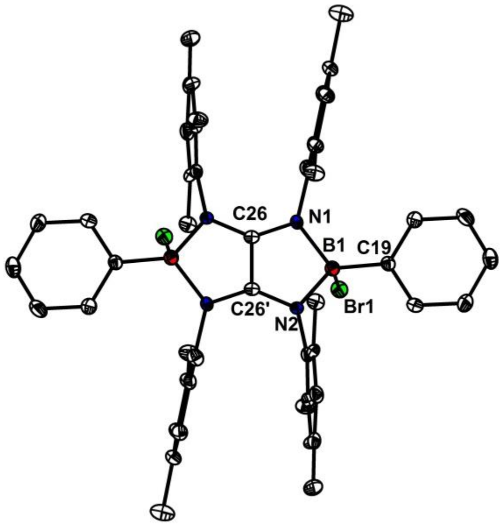

Figure S71. Solid structures of **3a** (hydrogen atoms are omitted for clarity). Thermal ellipsoids are set at the 50% probability. Selected bond lengths (Å) and angles (°): B1−N1 1.581(4), B1−N2 1.585(4), B1−C19 1.597(4), B1−Br1 2.109(3), N1−C26 1.327(4), N2−C26' 1.320(3), C26−C26' 1.466(5), N1−B1−N2 99.8(2), N1−B1−C19 120.5(3), N2−B1−C19 119.2(3), N1−B1−Br1 105.35(19), N2−B1−Br1 106.11(19), C19−B1−Br1 104.4(2).

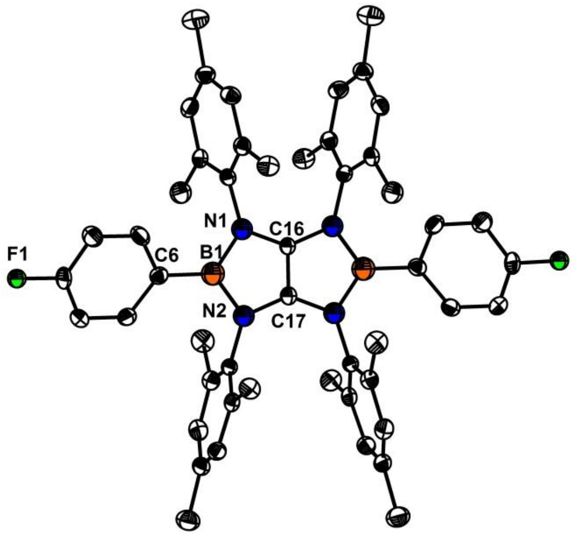

Figure S72. Solid structures of **4b** (hydrogen atoms are omitted for clarity). Thermal ellipsoids are set at the 50% probability. Selected bond lengths (Å) and angles (°): B1−N1 1.446(3), B1−N2 1.446(3), B1−C6 1.558(3), N1−C16 1.398(2), N2−C17 1.395(2), C16−C17 1.332(5), N1−B1−N2 106.9(2), N1−B1−C6 126.6(2), N2−B1−C6 126.5(2).

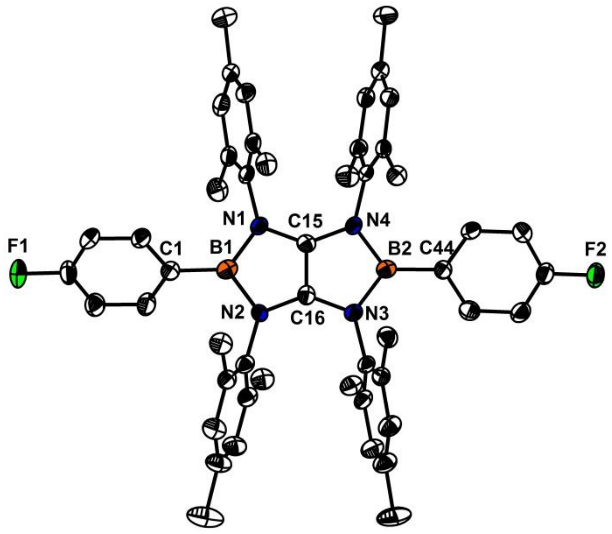

Figure S73. Solid structures of **4b**$^{\bullet+}$ (hydrogen atoms are omitted for clarity). Thermal ellipsoids are set at the 50% probability. y. Selected bond lengths (Å) and angles (°): B1−N1 1.458(6), B1−N2 1.473(6), B1−C1 1.539(6), B2−N3 1.473(6), B2−N4 1.468(5), B2−C44 1.537(6), N1−C15 1.362(5), N2−C16 1.361(5), N3−C16 1.360(5), N4−C15 1.371(5), C15−C16 1.395(5), N1−B1−N2 106.5(3), N1−B1−C1 128.1(4), N2−B1−C1 125.4(4), N3−B2−N4 106.0(3), N3−B2−C44 126.4(4), N4−B2−C44 127.5(4).

3. UV−vis absorption and photoluminescence spectroscopies

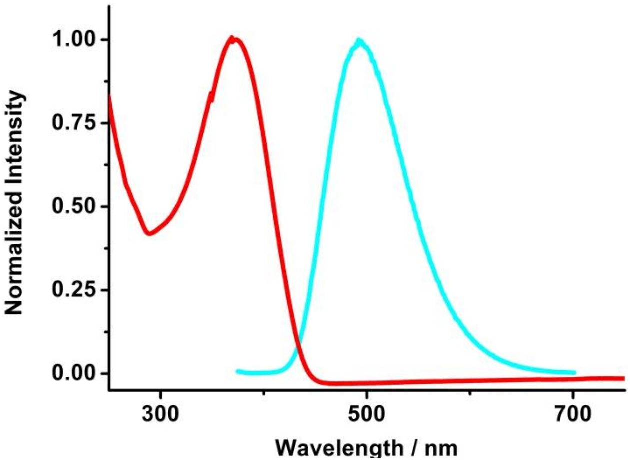

Figure S74. The normalized absorption (red line) and fluorescence (cyan line) spectra of **4a** in CH_2Cl_2.

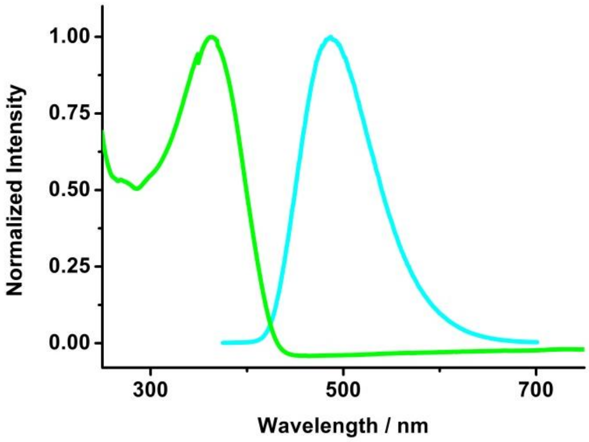

Figure S75. The normalized absorption (green line) and fluorescence (cyan line) spectra of **4b** in CH_2Cl_2.

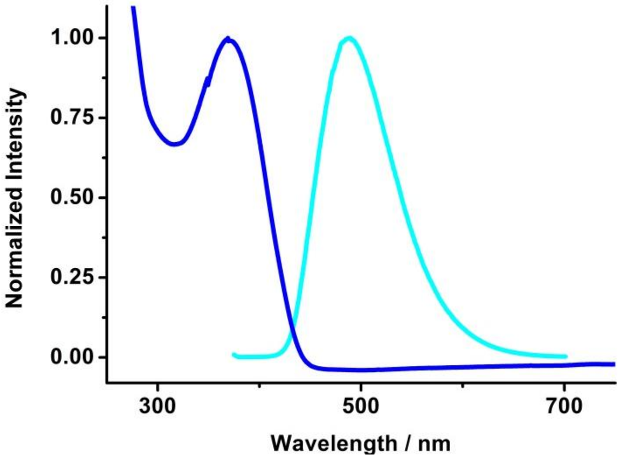

Figure S76. The normalized absorption (blue line) and fluorescence (cyan line) spectra of **4c** in CH_2Cl_2.

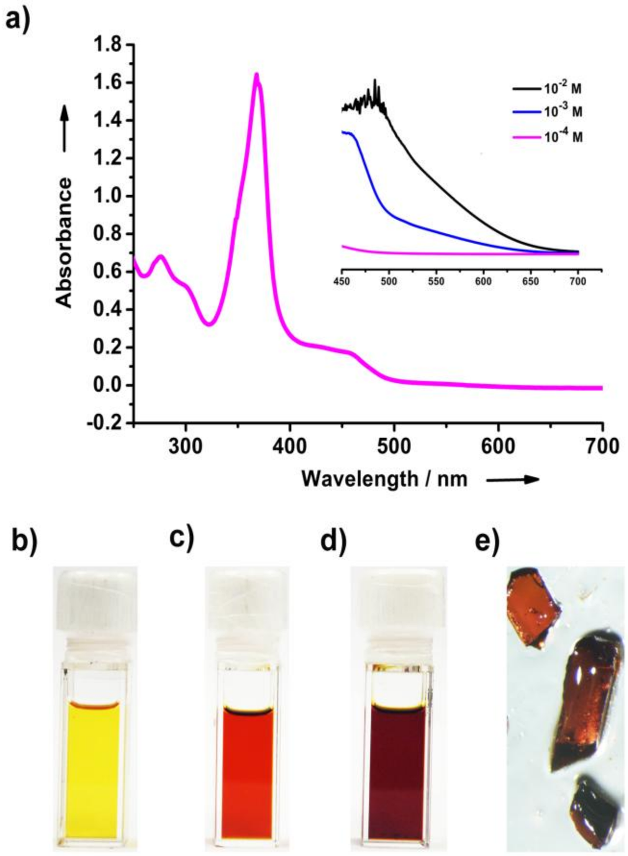

Figure S77 The absorption spectrum of **[4a$^{•+}$]•[B(C_6F_5)$_4$]$^-$** (a). Pictures of CH_2Cl_2 solutions of **[4a$^{•+}$]•[B(C_6F_5)$_4$]$^-$** [1×10^{-4} M (b) 1×10^{-3} M (c), 1×10^{-2} M (d). Crystals of **[4a$^{•+}$]•[B(C_6F_5)$_4$]$^-$** (e).

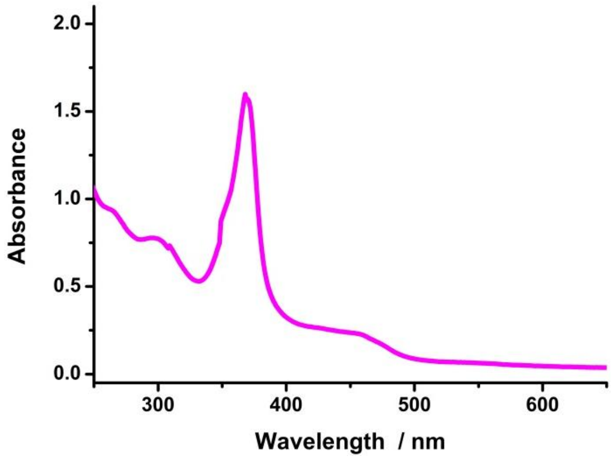

Figure S78. a) The absorption spectrum of **[4b$^{•+}$]•[B(C_6F_5)$_4$]$^-$** in CH_2Cl_2 .

4. Cyclic voltammograms

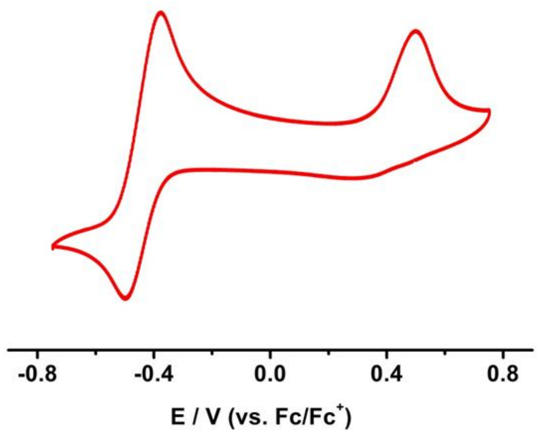

Figure S79. Cyclic voltammogram of **4a** in CH_2Cl_2/0.1 M [nBu_4N][PF_6] at room temperature. Scan rate: 100 mVs^{-1}.

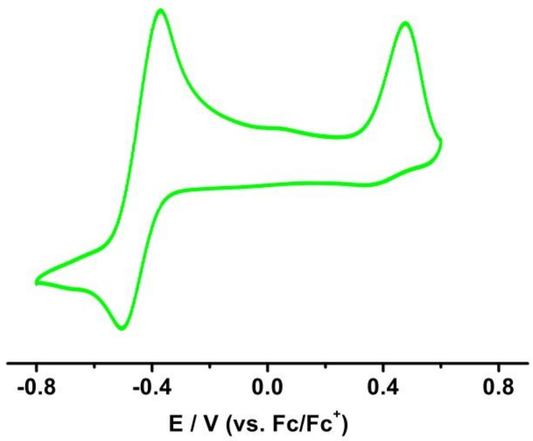

Figure S80. Cyclic voltammogram of **4b** in CH_2Cl_2/0.1 M [nBu_4N][PF_6] at room temperature. Scan rate: 100 mVs^{-1}.

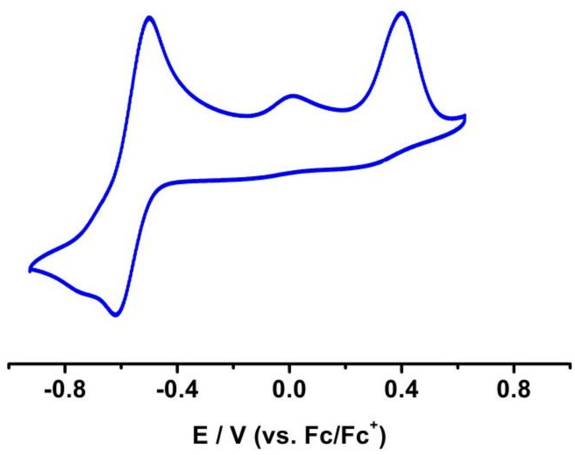

Figure S81. Cyclic voltammogram of **4c** in CH_2Cl_2/0.1 M [nBu_4N][PF_6] at room temperature. Scan rate: 100 mVs^{-1}.

5. EPR spectra

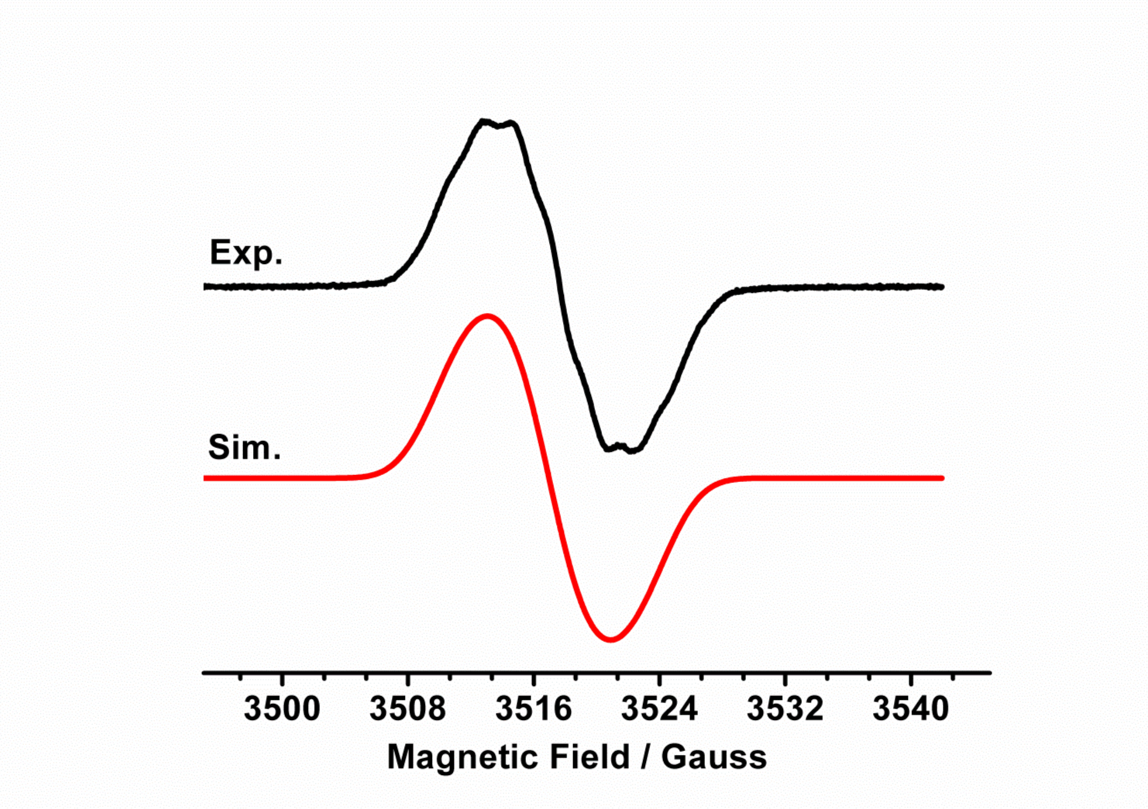

Figure S82. Experimental (top, black) EPR spectrum of **[4b$^{\bullet+}$]•[B(C$_6$F$_5$)$_4$]$^-$** in fluorobenzene at 298 K; simulated (bottom, red) EPR spectrum obtained using g = 2.0051, a(^{11}B) = 1.77 G, a(^{10}B) = 1.02 G, a(^{14}N) = 0.78 G, a (^{1}H) = 1.29 G.

6. Theoretical calculations

Gaussian 09 was used for all density functional theory (DFT) calculations including geometry optimization, frequency calculations, Natural bond orbital (NBO) analysis, nucleus-independent chemical-shift (NICS) calculations.[S6] Optimization, frequency calculations, NBO analysis were performed at the M062X/6-31G(d,p) level of theory. NICS calculations were performed at the M062X/6-311+G(d,p) level of theory. The UV-vis absorption spectrum was calculated for **4a** and **4a**$^{•+}$ using time-dependent DFT (TD-DFT) method at (U)M062x/6-31G(d, p) level of theory.

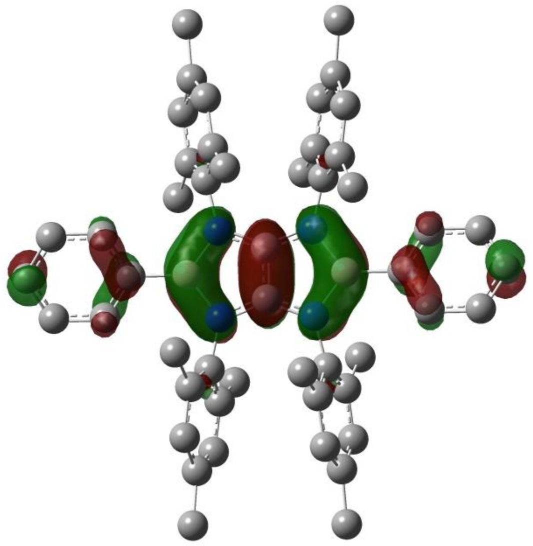

Figure S83. The plot of the SOMO of **4a**$^{•+}$.

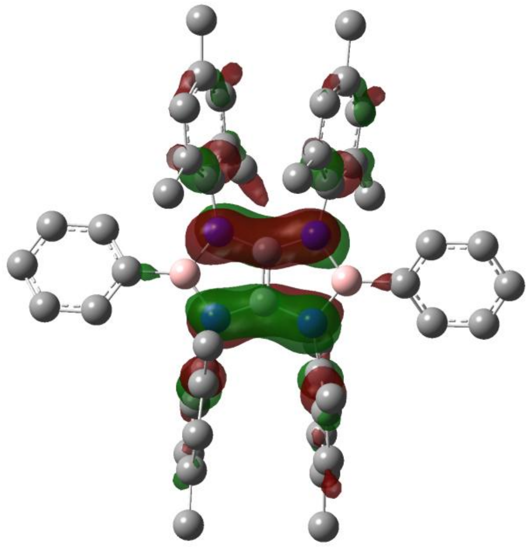

Figure S84. The plot of the HOMO−14 of **4a**.

Table S2: Calculated NICS(0) and NICS(1) values for **4'** and other related compounds at the M062X/6-311+G(d,p) level of theory.

	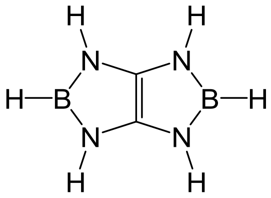 **4'**	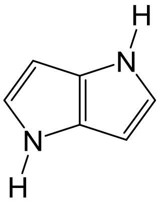	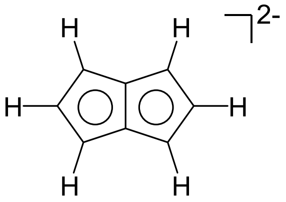
NICS(0)	−10.12	−13.06	−10.20
NICS(1)	−6.07	−9.71	−8.64

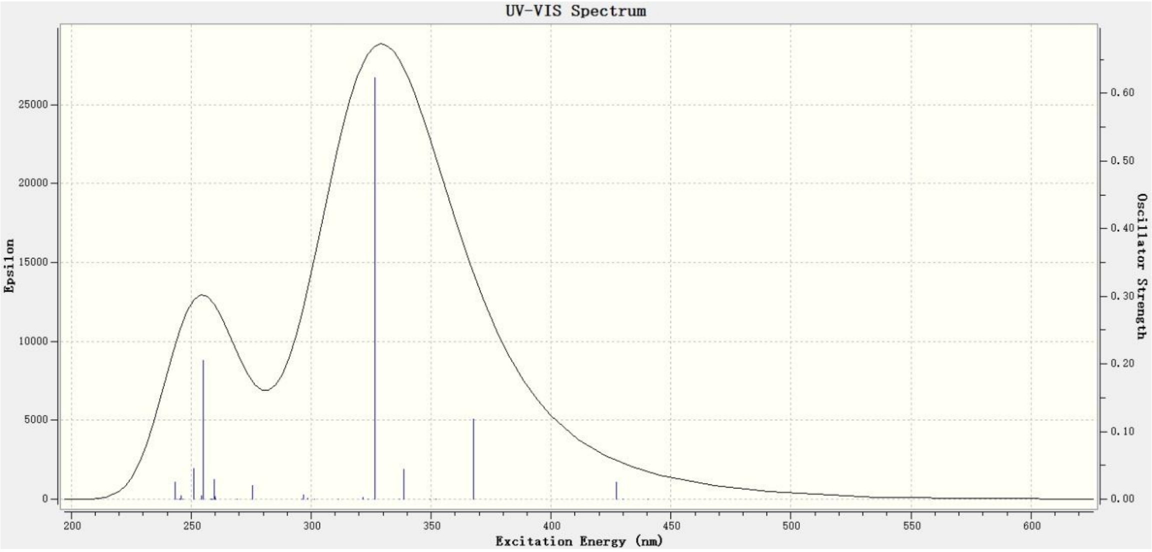

Figure S85. Calculated UV-Vis spectrum of **4a**$^{\bullet+}$ by TDDFT.

Table S3: TD-DFT vertical one-electron excitations calculated for **4a** and **4a**$^{\bullet+}$.

Molecular	Energy (ev)	Wavelength (nm)	Oscillator strength (f)	Contributions
**4a**	3.8330	323.5	0.6560	HOMO→LUMO (69.4%)
**4a**$^{\bullet+}$	2.9033	427.05	0.0246	194(β)→196(β) (97.8%)
	3.3717	367.73	0.1187	196(α)→197(α) (-24.0%)
				188(β)→196(β) (87.0%)
				184(β)→197(β) (15.7%)
	3.6597	338.78	0.0435	196(α)→197(α) (53.5%)
				188(β)→196(β) (35.3%)
				185(α)→198(α) (-15.8%)
				184(α)→197(α) (-11.5%)
	3.7976	326.48	0.6236	196(α)→197(α) (72.5%)
				196(α)→211(α) (14.9%)

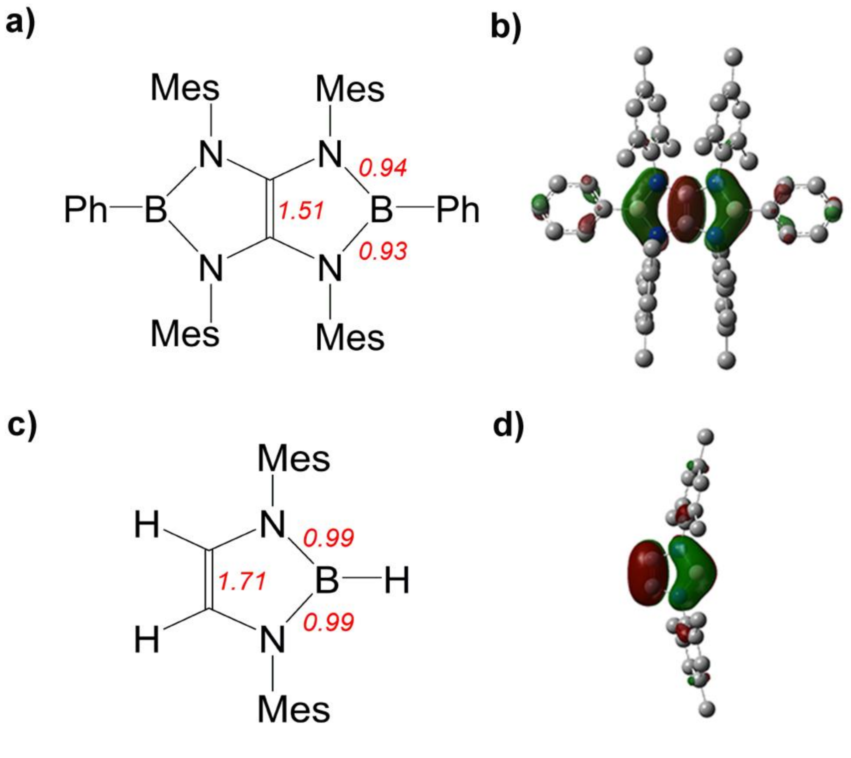

Figure S86. The Wiberg bond index values of **4a** (a) and 1,3-dimesityl-1,3,2-diazaborole (c) calculated at at the M062X/6-31G(d,p) level. The plots of the HOMO of **4a** (b) and 1,3-dimesityl-1,3,2-diazaborole (d) (H atoms are omitted for clarity).

Table S4: Optimized structures of **4a** and **4a**$^{\bullet+}$ (atom, x-, y-, z- positions in Å).

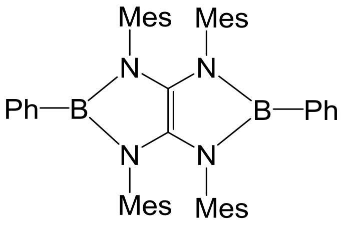

4a

Zero-point correction =	0.932520 (Hartree/Particle)
Thermal correction to Energy =	0.987400
Thermal correction to Enthalpy =	0.988344
Thermal correction to Gibbs Free Energy =	0.840378
Sum of electronic and zero-point Energies =	-2204.986008
Sum of electronic and thermal Energies =	-2204.986008
Sum of electronic and thermal Enthalpies =	-2204.930184
Sum of electronic and thermal Free Energies =	-2205.078150

B	0.037281	-2.164439	-0.230534
C	0.033009	-3.717924	-0.37713
C	-1.039755	-4.374898	-1.00396
H	-1.866789	-3.791464	-1.401068
C	-1.066969	-5.759933	-1.128717
H	-1.90715	-6.24244	-1.618702
C	-0.016462	-6.526257	-0.629479
H	-0.035741	-7.607502	-0.726086
C	1.059252	-5.897773	-0.008037
H	1.881956	-6.488403	0.383478
C	1.082001	-4.511423	0.115848
H	1.929922	-4.039173	0.605343
C	-2.52863	-1.610593	-0.104506
C	-3.069661	-2.23954	1.026287
C	-2.188355	-2.756063	2.13309
H	-1.73206	-3.710443	1.846815
H	-2.767138	-2.913463	3.046281
H	-1.369977	-2.064586	2.351042
C	-4.454937	-2.387313	1.097554
H	-4.886802	-2.855162	1.980084
C	-5.29841	-1.931336	0.085277
C	-6.795207	-2.008986	0.239472
H	-7.098233	-2.926118	0.751361
H	-7.297831	-1.975137	-0.730489
H	-7.163216	-1.163904	0.83299
C	-4.723176	-1.371572	-1.053792
H	-5.363081	-1.035525	-1.867448
C	-3.343978	-1.217066	-1.175502
C	-2.744227	-0.686458	-2.451105
H	-3.469518	-0.07748	-2.996203
H	-2.440293	-1.518276	-3.097692
H	-1.853698	-0.082375	-2.267573
C	-0.650539	-0.000031	0.000571
C	0.694771	0.000222	0.000902
C	2.56348	-1.565951	0.185368
C	2.910073	-1.597445	1.544331
C	1.830123	-1.543276	2.593951
H	1.192359	-0.659549	2.474645
H	2.261631	-1.528269	3.597559
H	1.177969	-2.422123	2.513865
C	4.255466	-1.711503	1.885848
H	4.535894	-1.717334	2.93739
C	5.24769	-1.812485	0.909723

C	6.704701	-1.846416	1.291563
H	7.107951	-0.829027	1.349634
H	7.297129	-2.394896	0.555022
H	6.8515	-2.31477	2.268019
C	4.859853	-1.86184	-0.427638
H	5.618073	-1.985302	-1.198437
C	3.524234	-1.745715	-0.814354
C	3.133529	-1.816947	-2.265607
H	3.000498	-0.815149	-2.684833
H	2.194521	-2.360607	-2.393557
H	3.911785	-2.320231	-2.843793
N	-1.133093	-1.306953	-0.144444
N	1.199181	-1.291703	-0.146644
B	0.036372	2.164898	0.229713
C	0.031352	3.718481	0.375208
C	-1.041639	4.375503	1.001588
H	-1.868357	3.79205	1.399324
C	-1.069493	5.760638	1.125087
H	-1.909845	6.243186	1.61474
C	-0.019417	6.527012	0.625018
H	-0.039218	7.608337	0.720625
C	1.056529	5.898478	0.004031
H	1.878898	6.489142	-0.388136
C	1.079914	4.512026	-0.118585
H	1.927995	4.039714	-0.607751
C	-2.52924	1.609971	0.104212
C	-3.070979	2.238513	-1.026089
C	-2.190762	2.755467	-2.133556
H	-1.737398	3.71177	-1.849004
H	-2.769653	2.909419	-3.047281
H	-1.370271	2.066001	-2.349855
C	-4.456673	2.385729	-1.096681
H	-4.889064	2.853281	-1.979064
C	-5.299224	1.929717	-0.084089
C	-6.796326	2.004301	-0.236093
H	-7.098064	2.891642	-0.798417
H	-7.294982	2.028079	0.736292
H	-7.170041	1.127675	-0.778135
C	-4.723001	1.37051	1.055126
H	-5.362413	1.034959	1.869427
C	-3.344031	1.216364	1.176016
C	-2.743201	0.686607	2.451477
H	-3.467861	0.077522	2.997295

H	-2.439224	1.518854	3.097485
H	-1.852494	0.082868	2.267684
C	2.562967	1.566875	-0.184505
C	2.909804	1.598275	-1.543418
C	1.830005	1.544037	-2.593202
H	1.191425	0.661003	-2.473112
H	2.261682	1.527629	-3.596716
H	1.178659	2.42358	-2.514146
C	4.255239	1.712335	-1.884718
H	4.535855	1.718098	-2.936209
C	5.247296	1.813374	-0.908412
C	6.704372	1.847341	-1.289996
H	7.107642	0.829959	-1.348044
H	7.296658	2.395794	-0.553321
H	6.851337	2.315748	-2.266402
C	4.859237	1.86273	0.428873
H	5.61733	1.986198	1.199799
C	3.523545	1.746613	0.815376
C	3.132729	1.817715	2.266608
H	3.002016	0.815808	2.686313
H	2.192526	2.359316	2.394375
H	3.909909	2.322994	2.844504
N	-1.133606	1.306881	0.14374
N	1.198624	1.292524	0.147175

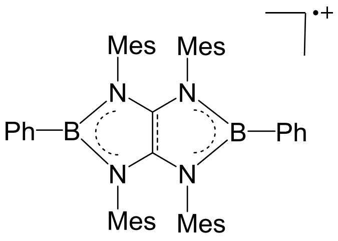

4a•+

Zero-point correction =	0.932874 (Hartree/Particle)
Thermal correction to Energy =	0.986078
Thermal correction to Enthalpy =	0.987022
Thermal correction to Gibbs Free Energy =	0.843998
Sum of electronic and zero-point Energies =	-2204.796429
Sum of electronic and thermal Energies =	-2204.743226
Sum of electronic and thermal Enthalpies =	-2204.742281
Sum of electronic and thermal Free Energies =	-2204.885305

B	0.019325	-2.160702	-0.00485
B	-0.019351	2.160733	-0.004635
C	0.033163	-3.699654	-0.008957
C	-1.045179	-4.432622	0.518532
H	-1.902197	-3.911707	0.937261
C	-1.030759	-5.822366	0.518882
H	-1.867464	-6.372495	0.936481
C	0.058697	-6.506785	-0.01606
H	0.068635	-7.591965	-0.018749
C	1.13526	-5.799924	-0.547839
H	1.981559	-6.332597	-0.968695
C	1.124437	-4.410188	-0.540576
H	1.971231	-3.871626	-0.957757
C	-2.566312	-1.610934	-0.082234
C	-3.079802	-2.092447	-1.29117
C	-2.190791	-2.450818	-2.454238
H	-1.785559	-3.460161	-2.324176
H	-1.338651	-1.772643	-2.555668
H	-2.753923	-2.43101	-3.389376
C	-4.461203	-2.269125	-1.380581
H	-4.881247	-2.629379	-2.316733
C	-5.312117	-1.98821	-0.312554
C	-6.805803	-2.099926	-0.463731
H	-7.280339	-2.390723	0.476236
H	-7.076968	-2.83161	-1.227682
H	-7.228906	-1.134669	-0.763573

C	-4.748584	-1.576168	0.896358
H	-5.394619	-1.386607	1.750648
C	-3.376323	-1.394315	1.040233
C	-2.784395	-1.033336	2.376991
H	-2.383404	-1.92722	2.86924
H	-3.54393	-0.600092	3.030734
H	-1.965369	-0.316125	2.29347
C	-0.705917	-0.006342	-0.001915
C	0.705908	0.006371	-0.001959
C	2.594111	-1.569652	0.081454
C	3.104555	-2.05567	1.292019
C	2.211583	-2.43226	2.446661
H	1.342745	-1.774155	2.538802
H	2.764767	-2.397824	3.387313
H	1.83076	-3.450795	2.31423
C	4.485193	-2.222394	1.390005
H	4.901379	-2.587308	2.326194
C	5.342595	-1.929574	0.328776
C	6.834747	-2.048132	0.492947
H	7.100647	-2.915212	1.101947
H	7.236219	-1.160228	0.99333
H	7.335088	-2.140908	-0.47335
C	4.785066	-1.508229	-0.877873
H	5.434953	-1.304554	-1.725984
C	3.410977	-1.335002	-1.031024
C	2.836945	-0.961694	-2.372748
H	1.945193	-0.337653	-2.28935
H	2.553257	-1.86277	-2.928436
H	3.573108	-0.415564	-2.966764
C	-2.594153	1.5696	0.081633
C	-3.41098	1.335036	-1.030872
C	-2.836982	0.961746	-2.372618
H	-3.573002	0.415236	-2.966458
H	-1.944958	0.338085	-2.289247
H	-2.553758	1.86286	-2.928486
C	-4.785091	1.508345	-0.877761
H	-5.434951	1.304727	-1.7259
C	-5.342639	1.929637	0.328868
C	-6.83476	2.048546	0.493099
H	-7.100507	2.917238	1.099879
H	-7.236083	1.162003	0.996
H	-7.335392	2.138795	-0.473287
C	-4.485253	2.222282	1.390193

H	-4.901466	2.58712	2.326402
C	-3.104643	2.055522	1.292239
C	-2.211642	2.431939	2.446912
H	-1.343292	1.773251	2.539478
H	-2.765043	2.398264	3.387457
H	-1.830074	3.450153	2.314157
C	-0.033173	3.699686	-0.008804
C	-1.124426	4.410195	-0.540509
H	-1.971274	3.871613	-0.957551
C	-1.135156	5.799929	-0.548033
H	-1.981443	6.332576	-0.968949
C	-0.058523	6.506824	-0.016444
H	-0.068385	7.592005	-0.019358
C	1.030905	5.822434	0.518592
H	1.867668	6.372586	0.936046
C	1.045231	4.432689	0.51851
H	1.90223	3.91181	0.937318
C	2.566314	1.610933	-0.082093
C	3.376229	1.394238	1.040412
C	2.784132	1.033267	2.377096
H	1.965739	0.315324	2.293549
H	2.382196	1.926988	2.868864
H	3.543798	0.600901	3.031259
C	4.748524	1.575997	0.896629
H	5.394496	1.386382	1.750949
C	5.312152	1.988032	-0.312232
C	6.805839	2.099678	-0.463466
H	7.280854	2.386615	0.477442
H	7.07705	2.834213	-1.224676
H	7.228419	1.135432	-0.767282
C	4.461312	2.269057	-1.380307
H	4.88143	2.629349	-2.316413
C	3.079903	2.092486	-1.29098
C	2.190931	2.451083	-2.454006
H	1.338817	1.772898	-2.555627
H	2.754102	2.431519	-3.389124
H	1.785639	3.460375	-2.323717
N	-1.165701	-1.282291	-0.000261
N	1.188869	-1.260988	-0.006163
N	-1.188889	1.261006	-0.005889
N	1.165683	1.282332	-0.000208

Table S5. The NPA charges of **4a** calculated at M062X/6-31G(d,p) level of theory

Atom	No	Natural Charge	Natural Population			
			Core	Valence	Rydberg	Total
B	1	0.9455	1.99892	2.03836	0.01723	4.0545
C	2	-0.38892	1.99892	4.37442	0.01558	6.38892
C	3	-0.21039	1.99913	4.19825	0.01302	6.21039
H	4	0.24516	0	0.7533	0.00154	0.75484
C	5	-0.24614	1.99912	4.23318	0.01385	6.24614
H	6	0.24426	0	0.75448	0.00125	0.75574
C	7	-0.2408	1.99912	4.22828	0.01339	6.2408
H	8	0.24293	0	0.75585	0.00122	0.75707
C	9	-0.24565	1.99912	4.23265	0.01388	6.24565
H	10	0.24404	0	0.75471	0.00125	0.75596
C	11	-0.21197	1.99912	4.20008	0.01277	6.21197
H	12	0.24558	0	0.75278	0.00164	0.75442
C	13	0.14963	1.99883	3.83396	0.01759	5.85037
C	14	-0.02084	1.99898	4.00739	0.01447	6.02084
C	15	-0.70968	1.99941	4.70289	0.00737	6.70968
H	16	0.26567	0	0.73295	0.00138	0.73433
H	17	0.24471	0	0.75418	0.00111	0.75529
H	18	0.24615	0	0.75263	0.00123	0.75385
C	19	-0.24127	1.99898	4.23038	0.0119	6.24127
H	20	0.23829	0	0.76024	0.00147	0.76171
C	21	-0.02353	1.99905	4.01025	0.01423	6.02353
C	22	-0.70792	1.99942	4.70146	0.00704	6.70792
H	23	0.24822	0	0.75059	0.00118	0.75178
H	24	0.24428	0	0.75456	0.00116	0.75572
H	25	0.24872	0	0.74991	0.00137	0.75128
C	26	-0.23527	1.99898	4.22388	0.01241	6.23527
H	27	0.23868	0	0.75984	0.00148	0.76132
C	28	-0.01681	1.99898	4.00342	0.01441	6.01681
C	29	-0.70446	1.99941	4.69738	0.00767	6.70446
H	30	0.24746	0	0.75133	0.00121	0.75254
H	31	0.25538	0	0.74339	0.00123	0.74462
H	32	0.24708	0	0.75149	0.00144	0.75292
C	33	0.29658	1.99859	3.68616	0.01866	5.70342
C	34	0.30764	1.99863	3.67435	0.01937	5.69236
C	35	0.1509	1.99882	3.83219	0.01809	5.8491
C	36	-0.02944	1.99898	4.01696	0.0135	6.02944
C	37	-0.71556	1.99942	4.70842	0.00773	6.71556
H	38	0.25482	0	0.74375	0.00143	0.74518

H	39	0.24723	0	0.75174	0.00103	0.75277
H	40	0.25608	0	0.74259	0.00134	0.74392
C	41	-0.24355	1.99898	4.23248	0.01209	6.24355
H	42	0.23909	0	0.75944	0.00147	0.76091
C	43	-0.02227	1.99906	4.00904	0.01417	6.02227
C	44	-0.708	1.99942	4.70159	0.00698	6.708
H	45	0.25074	0	0.7479	0.00136	0.74926
H	46	0.24565	0	0.75318	0.00116	0.75435
H	47	0.2462	0	0.75263	0.00117	0.7538
C	48	-0.23489	1.99899	4.22405	0.01185	6.23489
H	49	0.23789	0	0.76064	0.00147	0.76211
C	50	-0.00839	1.99897	3.99509	0.01434	6.00839
C	51	-0.70393	1.99941	4.69726	0.00726	6.70393
H	52	0.24807	0	0.75001	0.00192	0.75193
H	53	0.25964	0	0.73914	0.00123	0.74036
H	54	0.24522	0	0.75369	0.00109	0.75478
N	55	-0.6764	1.99919	5.66549	0.01172	7.6764
N	56	-0.67926	1.9992	5.66805	0.01201	7.67926
B	57	0.94546	1.99892	2.0384	0.01723	4.05454
C	58	-0.38885	1.99892	4.37436	0.01558	6.38885
C	59	-0.2104	1.99913	4.19825	0.01302	6.2104
H	60	0.24516	0	0.7533	0.00154	0.75484
C	61	-0.24615	1.99912	4.23318	0.01385	6.24615
H	62	0.24426	0	0.75448	0.00125	0.75574
C	63	-0.24081	1.99912	4.22829	0.01339	6.24081
H	64	0.24293	0	0.75585	0.00122	0.75707
C	65	-0.24566	1.99912	4.23266	0.01388	6.24566
H	66	0.24403	0	0.75471	0.00125	0.75597
C	67	-0.21196	1.99912	4.20007	0.01277	6.21196
H	68	0.24554	0	0.75281	0.00164	0.75446
C	69	0.1496	1.99883	3.83398	0.01759	5.8504
C	70	-0.02089	1.99898	4.00744	0.01447	6.02089
C	71	-0.70971	1.99941	4.70292	0.00738	6.70971
H	72	0.26567	0	0.73295	0.00138	0.73433
H	73	0.2447	0	0.75419	0.00111	0.7553
H	74	0.24617	0	0.7526	0.00123	0.75383
C	75	-0.24139	1.99898	4.2305	0.0119	6.24139
H	76	0.2383	0	0.76023	0.00147	0.7617
C	77	-0.02347	1.99905	4.01018	0.01423	6.02347
C	78	-0.70766	1.99942	4.7012	0.00704	6.70766
H	79	0.24759	0	0.75123	0.00117	0.75241
H	80	0.24483	0	0.75401	0.00117	0.75517
H	81	0.2486	0	0.75001	0.00138	0.7514

C	82	-0.23514	1.99898	4.22375	0.01241	6.23514
H	83	0.23859	0	0.75992	0.00149	0.76141
C	84	-0.01687	1.99898	4.00348	0.01441	6.01687
C	85	-0.70446	1.99941	4.69739	0.00767	6.70446
H	86	0.24744	0	0.75134	0.00121	0.75256
H	87	0.25538	0	0.74339	0.00123	0.74462
H	88	0.24706	0	0.75151	0.00144	0.75294
C	89	0.1509	1.99882	3.83219	0.01809	5.8491
C	90	-0.02945	1.99898	4.01696	0.0135	6.02945
C	91	-0.71557	1.99942	4.70843	0.00773	6.71557
H	92	0.25481	0	0.74376	0.00143	0.74519
H	93	0.24724	0	0.75173	0.00103	0.75276
H	94	0.2561	0	0.74256	0.00134	0.7439
C	95	-0.24353	1.99898	4.23246	0.01209	6.24353
H	96	0.2391	0	0.75944	0.00147	0.7609
C	97	-0.02225	1.99906	4.00902	0.01417	6.02225
C	98	-0.708	1.99942	4.70159	0.00698	6.708
H	99	0.25074	0	0.7479	0.00136	0.74926
H	100	0.24565	0	0.75318	0.00116	0.75435
H	101	0.2462	0	0.75262	0.00117	0.7538
C	102	-0.23488	1.99899	4.22404	0.01185	6.23488
H	103	0.23789	0	0.76065	0.00147	0.76211
C	104	-0.00839	1.99897	3.99508	0.01434	6.00839
C	105	-0.70396	1.99941	4.69729	0.00726	6.70396
H	106	0.24807	0	0.75001	0.00192	0.75193
H	107	0.25963	0	0.73914	0.00123	0.74037
H	108	0.24525	0	0.75366	0.00109	0.75475
N	109	-0.67636	1.99919	5.66545	0.01171	7.67636
N	110	-0.67927	1.9992	5.66805	0.01201	7.67927
* Total *		0	111.94908	279.26812	0.78280	392.00000

Table S6. The NPA charges of **4a**$^{\bullet+}$ calculated at (U)M062X/6-31G(d,p) level of theory.

Atom	No	Natural Charge	Natural Population			
			Core	Valence	Rydberg	Total
B	1	1.06023	1.99895	1.9229	0.01792	3.93977
B	2	1.06022	1.99895	1.9229	0.01792	3.93978
C	3	-0.45293	1.99884	4.43912	0.01496	6.45293
C	4	-0.19302	1.99913	4.18071	0.01318	6.19302
H	5	0.24943	0	0.74901	0.00156	0.75057
C	6	-0.24128	1.99912	4.22803	0.01413	6.24128

H	7	0.25864	0	0.74019	0.00117	0.74136
C	8	-0.20985	1.99913	4.19723	0.01349	6.20985
H	9	0.25883	0	0.74002	0.00114	0.74117
C	10	-0.24131	1.99912	4.22806	0.01412	6.24131
H	11	0.25858	0	0.74025	0.00117	0.74142
C	12	-0.19277	1.99913	4.18046	0.01319	6.19277
H	13	0.24963	0	0.74882	0.00156	0.75037
C	14	0.11796	1.99879	3.86541	0.01784	5.88204
C	15	-0.01982	1.99898	4.00647	0.01437	6.01982
C	16	-0.71555	1.99941	4.70839	0.00775	6.71555
H	17	0.27238	0	0.72634	0.00128	0.72762
H	18	0.23779	0	0.76104	0.00117	0.76221
H	19	0.26086	0	0.73811	0.00103	0.73914
C	20	-0.2353	1.99898	4.22419	0.01213	6.2353
H	21	0.25077	0	0.74784	0.00139	0.74923
C	22	-0.00438	1.99906	3.99115	0.01416	6.00438
C	23	-0.71454	1.99942	4.70767	0.00745	6.71454
H	24	0.25555	0	0.74335	0.00109	0.74445
H	25	0.25626	0	0.74267	0.00107	0.74374
H	26	0.25633	0	0.74243	0.00124	0.74367
C	27	-0.22951	1.99898	4.21809	0.01244	6.22951
H	28	0.24984	0	0.74876	0.0014	0.75016
C	29	-0.01839	1.99898	4.00506	0.01436	6.01839
C	30	-0.70611	1.99941	4.69889	0.00782	6.70611
H	31	0.26223	0	0.73655	0.00121	0.73777
H	32	0.26123	0	0.73767	0.0011	0.73877
H	33	0.23276	0	0.76569	0.00154	0.76724
C	34	0.45632	1.99887	3.52502	0.01979	5.54368
C	35	0.45633	1.99887	3.52501	0.01979	5.54367
C	36	0.11769	1.99879	3.8657	0.01782	5.88231
C	37	-0.0203	1.99898	4.00709	0.01424	6.0203
C	38	-0.71571	1.99941	4.70855	0.00775	6.71571
H	39	0.23721	0	0.76163	0.00116	0.76279
H	40	0.26128	0	0.73768	0.00103	0.73872
H	41	0.27252	0	0.7262	0.00128	0.72748
C	42	-0.23353	1.99898	4.2224	0.01214	6.23353
H	43	0.25071	0	0.7479	0.00139	0.74929
C	44	-0.00503	1.99906	3.99182	0.01415	6.00503
C	45	-0.71542	1.99942	4.70857	0.00743	6.71542
H	46	0.25892	0	0.73999	0.00109	0.74108
H	47	0.25676	0	0.74204	0.00119	0.74324
H	48	0.25305	0	0.74588	0.00107	0.74695
C	49	-0.23029	1.99898	4.21887	0.01244	6.23029

H	50	0.24989	0	0.74871	0.0014	0.75011
C	51	-0.01775	1.99898	4.00442	0.01435	6.01775
C	52	-0.70686	1.99941	4.69959	0.00786	6.70686
H	53	0.23241	0	0.76613	0.00146	0.76759
H	54	0.26421	0	0.73461	0.00118	0.73579
H	55	0.26098	0	0.73786	0.00116	0.73902
C	56	0.11768	1.99879	3.8657	0.01782	5.88232
C	57	-0.01774	1.99898	4.00441	0.01435	6.01774
C	58	-0.70687	1.99941	4.69959	0.00786	6.70687
H	59	0.26098	0	0.73786	0.00116	0.73902
H	60	0.23241	0	0.76613	0.00146	0.76759
H	61	0.26422	0	0.7346	0.00118	0.73578
C	62	-0.2303	1.99898	4.21888	0.01244	6.2303
H	63	0.2499	0	0.7487	0.0014	0.7501
C	64	-0.00504	1.99906	3.99183	0.01415	6.00504
C	65	-0.71544	1.99942	4.70859	0.00743	6.71544
H	66	0.25896	0	0.73995	0.00109	0.74104
H	67	0.25677	0	0.74204	0.00119	0.74323
H	68	0.25302	0	0.74591	0.00107	0.74698
C	69	-0.23352	1.99898	4.2224	0.01214	6.23352
H	70	0.25071	0	0.7479	0.00139	0.74929
C	71	-0.02031	1.99898	4.00709	0.01424	6.02031
C	72	-0.7157	1.99941	4.70854	0.00775	6.7157
H	73	0.23721	0	0.76162	0.00116	0.76279
H	74	0.26128	0	0.73769	0.00103	0.73872
H	75	0.27251	0	0.72621	0.00128	0.72749
C	76	-0.45293	1.99884	4.43912	0.01496	6.45293
C	77	-0.19277	1.99913	4.18046	0.01319	6.19277
H	78	0.24963	0	0.74881	0.00156	0.75037
C	79	-0.2413	1.99912	4.22806	0.01412	6.2413
H	80	0.25858	0	0.74025	0.00117	0.74142
C	81	-0.20985	1.99913	4.19723	0.01349	6.20985
H	82	0.25883	0	0.74002	0.00114	0.74117
C	83	-0.24128	1.99912	4.22803	0.01413	6.24128
H	84	0.25864	0	0.74019	0.00117	0.74136
C	85	-0.19303	1.99913	4.18073	0.01318	6.19303
H	86	0.24943	0	0.74901	0.00156	0.75057
C	87	0.11797	1.99879	3.8654	0.01784	5.88203
C	88	-0.01839	1.99898	4.00505	0.01436	6.01839
C	89	-0.7061	1.99941	4.69888	0.00782	6.7061
H	90	0.23277	0	0.76568	0.00154	0.76723
H	91	0.26221	0	0.73657	0.00121	0.73779
H	92	0.26123	0	0.73767	0.0011	0.73877

C	93	-0.22953	1.99898	4.21811	0.01244	6.22953
H	94	0.24984	0	0.74876	0.0014	0.75016
C	95	-0.00438	1.99906	3.99116	0.01416	6.00438
C	96	-0.71455	1.99942	4.70768	0.00745	6.71455
H	97	0.2555	0	0.74341	0.00109	0.7445
H	98	0.25631	0	0.74262	0.00107	0.74369
H	99	0.25634	0	0.74242	0.00124	0.74366
C	100	-0.23529	1.99898	4.22418	0.01213	6.23529
H	101	0.25078	0	0.74784	0.00139	0.74922
C	102	-0.01982	1.99898	4.00647	0.01437	6.01982
C	103	-0.71555	1.99941	4.70838	0.00775	6.71555
H	104	0.2378	0	0.76103	0.00117	0.7622
H	105	0.26086	0	0.73811	0.00103	0.73914
H	106	0.27238	0	0.72634	0.00128	0.72762
N	107	-0.65087	1.99917	5.63853	0.01317	7.65087
N	108	-0.65074	1.99917	5.63843	0.01313	7.65074
N	109	-0.65073	1.99917	5.63843	0.01313	7.65073
N	110	-0.65087	1.99917	5.63853	0.01317	7.65087
* Total *		0	111.94933	278.25637	0.79431	391.00000

Table S7: Optimized structures of **4'** and related compounds **(**atom, x-, y-, z- positions in Å).

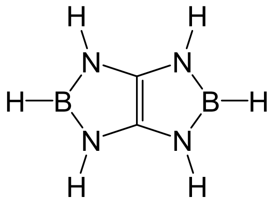

4'

N	0.35085800	1.70484900	-0.00000900
B	1.73576900	1.31084800	0.00002100
N	1.73576900	-0.12919800	-0.00001000
C	0.40565700	-0.53735100	-0.00006700
C	-0.40565700	0.53735100	-0.00006700
N	-0.35085800	-1.70484900	-0.00000900
B	-1.73576900	-1.31084800	0.00002100

N	-1.73576900	0.12919800	-0.00001000
H	-0.04373000	2.62868900	0.00018900
H	2.67852100	2.02326300	0.00005200
H	2.51646800	-0.76133900	0.00018700
H	0.04373000	-2.62868900	0.00018900
H	-2.67852100	-2.02326300	0.00005200
H	-2.51646800	0.76133900	0.00018700

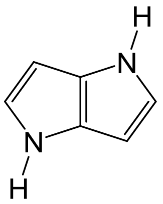

N	1.32578800	1.08653600	0.00000000
C	2.11742500	-0.04326800	0.00000000
C	1.33213500	-1.17370300	0.00000000
C	-0.00764400	-0.69420400	0.00000000
C	0.00764400	0.69420400	0.00000000
N	-1.32578800	-1.08653600	0.00000000
C	-2.11742500	0.04326800	0.00000000

C	-1.33213500	1.17370300	0.00000000
H	1.66926000	2.03031100	0.00000100
H	3.19182200	0.04167900	0.00000000
H	1.69377400	-2.18843700	-0.00000100
H	-1.66926000	-2.03031100	0.00000000
H	-3.19182200	-0.04167900	0.00000000
H	-1.69377400	2.18843700	0.00000000

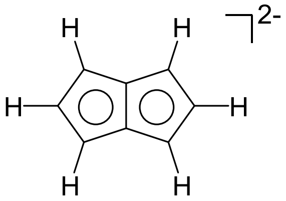

C	-1.36543500	1.16368500	0.00000500
C	-2.17991000	0.00000000	0.00000500
C	-1.36543500	-1.16368500	-0.00000300
C	0.00000000	-0.72092000	-0.00000300
C	0.00000000	0.72092000	-0.00000300
C	1.36543500	-1.16368500	-0.00000400
C	2.17991000	0.00000000	0.00001400

C	1.36543500	1.16368600	-0.00001200
H	-1.72946900	2.18823100	-0.00000900
H	-3.26978700	0.00000000	0.00000600
H	-1.72946800	-2.18823100	0.00000900
H	1.72946900	-2.18823100	-0.00002200
H	3.26978700	0.00000000	0.00002600
H	1.72946800	2.18823100	-0.00000200

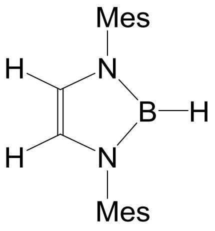

N	-1.13458000	-0.18693100	0.39155600
N	1.13461400	-0.18646000	0.39185300
B	0.00005200	0.15103500	-0.41405900
H	0.00014200	0.60484400	-1.51049800
C	-0.67551700	-0.69441900	1.61178800
H	-1.36208600	-0.99511600	2.38961200
C	0.67545900	-0.69418500	1.61194000
H	1.36195700	-0.99463400	2.38992200

C	-2.52154800	-0.05150700	0.09668600
C	-3.29149800	-1.19606600	-0.14573100
C	-4.64889500	-1.03683700	-0.42190200
H	-5.25326200	-1.92093700	-0.61385900
C	-5.24529900	0.22206000	-0.46904600
C	-4.45008000	1.34145100	-0.22608700
H	-4.90117100	2.33119000	-0.25271900
C	-3.09034900	1.22811900	0.05856000

C	-2.24913500	2.44870200	0.32744100		C	2.65827000	-2.56280000	-0.12699000
H	-1.65486600	2.32303900	1.23752600		H	2.37316600	-2.86294800	0.88612200
H	-2.87882600	3.33404100	0.43818200		H	3.34747400	-3.31199600	-0.52166900
H	-1.54459500	2.63208600	-0.49015100		H	1.74386100	-2.57381100	-0.72753600
C	-2.65891100	-2.56302000	-0.12624400		C	2.24968300	2.44876600	0.32850600
H	-2.37401900	-2.86301200	0.88696400		H	2.87934600	3.33436500	0.43725400
H	-1.74443300	-2.57443800	-0.72667400		H	1.65726700	2.32370200	1.23988200
H	-3.34827400	-3.31211400	-0.52084700		H	1.54340800	2.63120000	-0.48779800
C	2.52158700	-0.05135200	0.09684700		C	6.70673600	0.37287400	-0.80605000
C	3.29120000	-1.19600100	-0.14607200		H	6.84639700	0.51835700	-1.88245000
C	4.64862500	-1.03700000	-0.42237400		H	7.14267500	1.23721700	-0.29880100
H	5.25273100	-1.92117500	-0.61477100		H	7.27415100	-0.51503700	-0.51715600
C	5.24530600	0.22174500	-0.46915100		C	-6.70670300	0.37342600	-0.80598400
C	4.45036800	1.34128400	-0.22575400		H	-6.84631000	0.51805800	-1.88250100
H	4.90168300	2.33092900	-0.25224400		H	-7.27441300	-0.51404400	-0.51631400
C	3.09067100	1.22816600	0.05902200		H	-7.14231500	1.23836500	-0.29947400

7. References

S1 Kaufmann, D. *Chem. Ber.* 1987, **120**, 853.

S2 Shuji Yasuike, S.;Nakata, K.; Qin, W.; Matsumura, M.; Kakusawa, N.; Kurita, J. *J. Organometal. Chem.* 2015, **788**, 9.

S3 Qin, Y.; Kiburu, I.; Shah, S.; Jäkle, F. *Org. Lett.* 2006, **8**, 5227.

S4 Lindauer, D.; Beckert, R.; Doring, M.; Fehling, P.; Görls, H. *J. Prakt. Chem.* 1995, **337**, 143.

S5 Bruker AXS SHELXTL, Madison, WI; *SHELX-97G*. M. Sheldrick, *Acta Crystallogr. A*, 2008, **64**, 112–122, *SHELX-2013*, http://shelx.uni-ac. gwdg.de/SHELX/index.php.

S6 Gaussian 09, Revision B.01, M. J. Frisch, G. W. Trucks, H. B. Schlegel, G. E. Scuseria, M. A. Robb, J. R. Cheeseman, G. Scalmani, V. Barone, B. Mennucci, G. A. Petersson, H. Nakatsuji, M. Caricato, X. Li, H. P. Hratchian, A. F. Izmaylov, J. Bloino, G. Zheng, J. L. Sonnenberg, M. Hada, M. Ehara, K. Toyota, R. Fukuda, J. Hasegawa, M. Ishida, T. Nakajima, Y. Honda, O. Kitao, H. Nakai, T. Vreven, J. A. Montgomery, Jr., J. E. Peralta, F. Ogliaro, M. Bearpark, J. J. Heyd, E. Brothers, K. N. Kudin, V. N. Staroverov, T. Keith, R. Kobayashi, J. Normand, K. Raghavachari, A. Rendell, J. C. Burant, S. S. Iyengar, J. Tomasi, M. Cossi, N. Rega, J. M. Millam, M. Klene, J. E. Knox, J. B. Cross, V. Bakken, C. Adamo, J. Jaramillo, R. Gomperts, R. E. Stratmann, O. Yazyev, A. J. Austin, R. Cammi, C. Pomelli, J. W. Ochterski, R. L. Martin, K. Morokuma, V. G. Zakrzewski, G. A. Voth, P. Salvador, J. J. Dannenberg, S. Dapprich, A. D. Daniels, O. Farkas, J. B. Foresman, J. V. Ortiz, J. Cioslowski, D. J. Fox, Gaussian, Inc., Wallingford CT, 2010.
